# Depressive patient‐derived GABA interneurons reveal abnormal neural activity associated with HTR2C

**DOI:** 10.15252/emmm.202216364

**Published:** 2022-11-14

**Authors:** Kaiqin Lu, Yuan Hong, Mengdan Tao, Luping Shen, Zhilong Zheng, Kaiheng Fang, Fang Yuan, Min Xu, Chun Wang, Dongya Zhu, Xing Guo, Yan Liu

**Affiliations:** ^1^ Institute for Stem Cell and Neural Regeneration, State Key Laboratory of Reproductive Medicine School of Pharmacy Nanjing Medical University Nanjing China; ^2^ Department of Neurobiology Key Laboratory of Human Functional Genomics of Jiangsu Province Nanjing Medical University Nanjing China; ^3^ Nanjing Brain Hospital Affiliated to Nanjing Medical University Nanjing China; ^4^ Co‐innovation Center of Neuroregeneration Nantong University Jiangsu China

**Keywords:** disease modeling, GABAergic interneuron, iPSCs, major depressive disorder, organoids, Neuroscience

## Abstract

Major depressive disorder with suicide behavior (sMDD) is a server mood disorder, bringing tremendous burden to family and society. Although reduced gamma amino butyric acid (GABA) level has been observed in postmortem tissues of sMDD patients, the molecular mechanism by which GABA levels are altered remains elusive. In this study, we generated induced pluripotent stem cells (iPSC) from five sMDD patients and differentiated the iPSCs to GABAergic interneurons (GINs) and ventral forebrain organoids. sMDD GINs exhibited altered neuronal morphology and increased neural firing, as well as weakened calcium signaling propagation, compared with controls. Transcriptomic sequencing revealed that a decreased expression of serotoninergic receptor 2C (5‐HT2C) may cause the defected neuronal activity in sMDD. Furthermore, targeting 5‐HT2C receptor, using a small molecule agonist or genetic approach, restored neuronal activity deficits in sMDD GINs. Our findings provide a human cellular model for studying the molecular mechanisms and drug discoveries for sMDD.

## Graphical abstract

iPSC‐derived GABAergic interneurons generated from major depressive disorders with suicide behavior (sMDD) patients exhibit decreased 5‐HT2C expression. Trazodone hydrochloride (Trzd) that targets 5‐HT2C restores neuronal deficits, indicating that 5‐HT2C could be a therapeutic target for sMDD.

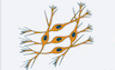

## Introduction

Major depressive disorder (MDD) is a leading cause of disability, with a global prevalence of 4.4–5.0% (WHO, 2017). Individuals with MDD often display loss of pleasure, sleep disorders, and mental disturbances (First *et al*, 2015). So far, the pathogenesis of MDD is elusive, at least partly due to the highly heterogeneity in symptoms and etiology. Thus, studying one subgroup of MDD, such as MDD with suicide behavior (sMDD), which shares a common symptom, may open a window to revealing the pathogenesis of MDD.

Severe MDD patients who have committed suicide pose a serious burden for families and communities (Ferrari *et al*, 2013). Neuroimaging and postmortem studies have reported altered subcortical brain structures (Krishnan & Nestler, 2008; Murrough *et al*, 2016; Belleau *et al*, 2019) in sMDD, including a decreased volume of the dentate gyrus (DG) (MacQueen *et al*, 2008) and reduced nerve growth factor levels (Wohleb *et al*, 2016). Immunohistochemical evidence from postmortem brain slices showed GABA_B_ receptor‐mediated inhibition is dysregulated, suggesting that GABAergic interneurons (GINs) were involved in regulation of glutamate‐glutamine‐GABA cycle in suicidal individuals (Bernstein *et al*, 2013; Lewis *et al*, 2018). An integrative genomics study showed that GABA_A_ receptor, gamma 2 (GABRG2), exhibited lower expression in postmortem suicidal individuals (Yin *et al*, 2016). Also, in the female veterans with suicidal behavior, the ratio of GABA over creatine^+^ phosphocreatine in anterior cingulate cortex was significantly reduced (Prescot *et al*, 2018). Some of these cellular and biochemical changes, including reduction of GABA transmission and GABA‐synthesizing enzyme 67 (GAD67) protein levels, as well as GABAA/B receptor deficits, are observed in animal models of MDD (Luscher *et al*, 2011; Banasr *et al*, 2017; Jacobson *et al*, 2018; Duman *et al*, 2019), leading to the hypothesis of disrupted structural and functional integrity of prefrontal GABAergic networks as a pathophysiological basis of MDD. Clinically, elevating GABA levels in MDD patients, by such as Brexanolone (SAGE‐547) or transcranial stimulation, has been developed as antidepressant treatments (Kanes *et al*, 2017; Heimrath *et al*, 2020). However, whether the GABA system could be a therapeutic target for sMDD patients is still unclear. Also, it remains a big challenge to study the etiology and potential therapeutic drugs for sMDD in animal models (Preti, 2011; Locci & Pinna, 2019).

Induced pluripotent stem cells (iPSCs) derived from neurological patients offer a potential model for studying pathogenesis and drug targets (Fujimori *et al*, 2018). Differentiation of iPSCs‐derived from bipolar disorder (BD) patients into hippocampal dentate gyrus‐like neurons showed mitochondrial abnormalities and altered neuronal excitation in BD patients (Mertens *et al*, 2015). Furthermore, two studies exhibited abnormal disease pathophysiology with iPSCs derived forebrain neurons and organoids in psychiatric disorders and Fragile X syndrome (Wen *et al*, 2014; Kang *et al*, 2021). Two recent studies reported longer neurites and serotonin‐induced hyperactivity downstream of upregulated excitatory serotonergic receptors respectively in SSRI‐resistant MDD iPSC‐derived serotoninergic neurons and default differentiated forebrain neurons (Vadodaria *et al*, 2019a,b). These iPSC‐derived cells provided human cellular disease models for mechanism studies and drug discoveries. However, a human iPSC model for sMDD has not been reported.

Here, we generated iPSCs from five sMDD patients who attempted suicide at least once and differentiated patient‐specific iPSCs to GINs and ventral forebrain organoids that included GINs. We found that the serotoninergic receptor 2C subtype (5‐HT2C) decreased in sMDD GINs, which was reversed by an FDA‐approved small molecule that targeted 5‐HT2C receptors. Taken together, our current study revealed important cellular phenotypes for sMDD in human neurons and ventral forebrain organoids, offering the potential tool for identifying therapeutic approaches.

## Results

### sMDD iPSC‐derived GINs display increased neurite branches and abnormal GIN subtypes

**Figure 1:**
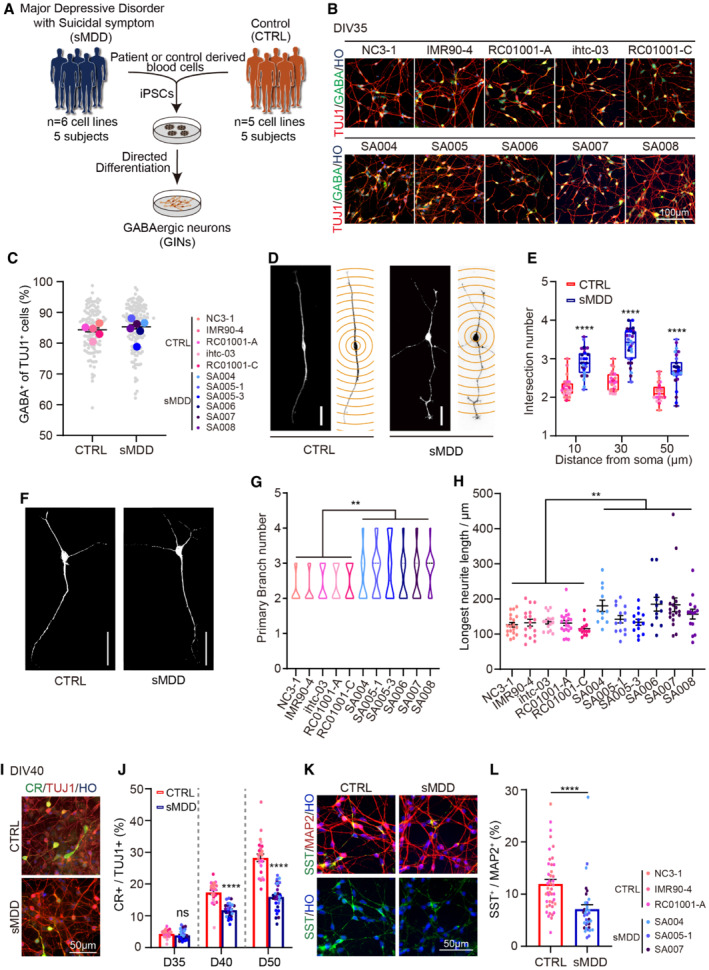
Differentiation of GINs and abnormal neural morphology and GINs subtypes expression in sMDD GINs Scheme illustrating the generation of GINs from CTRL and sMDD patient‐derived iPSCs (five cell lines from five CTRLs and six cell lines from five patients).Representative images of GINs cultures showing GABA and TUJ1 expression. Scale bar = 100 μm.The proportion of GABA^+^ cells of TUJ1^+^ neurons from both CTRL and sMDD iPSCs derived GINs at day 35. (CTRL, *n* = 120 fields were counted from five cell lines; sMDD, *n* = 134 fields were counted from 6 cell lines). Mean ratio ± SEM.Representative images and schematic morphometric Sholl analysis from CTRL and sMDD GINs at day 35. Scale bar = 50 μm.Sholl intersection number of CTRL and sMDD GINs at day 35 (Each red box is from five CTRL cell lines, *n* = 25 independently differentiations from five cell lines, each point represents mean value of neurons from five independent experiments, *n* = 226 neurons from CTRL groups. Each blue box is from six sMDD cell lines, *n* = 30 differentiations independently from six cell lines, each point represents mean value of neurons from five independent experiments, *n* = 260 neurons from sMDD groups). Two‐way ANOVA for different distance from soma, ****P < 0.0001 for 10, 30 and 50 μm. The center line shows the median the box shows SEM and the whiskers represent the maximum and minimum.Representative images of GINs from CTRL and sMDD at day 65. Scale bar = 50 μm.Quantification of primary branch numbers of GINs shown in five CTRL cell lines and six sMDD cell lines at day 65, *n* ≥ 11, Nested *t*‐test, **P = 0.0016 for CTRL versus sMDD. The center line shows the median. Mean ratio ± SEM.Quantification of longest neurite length of GINs shown in five CTRL cell lines and six sMDD cell lines at day 65, *n* ≥ 11, Nested *t*‐test, **P = 0.0075 for CTRL versus sMDD. Mean ratio ± SEM.Representative images of GINs subtype calretinin (CR) expression. Scale bar = 50 μm.The proportion of CR^+^ cells of TUJ1^+^ neurons from both CTRL and sMDD iPSCs derived GINs at days 35, 40 and 50, respectively. (Red bar from five CTRL cell lines, blue bar from six sMDD cell lines). *n* = 5 in each cell line. Two‐way ANOVA for timepoints, ns: no significant at day 35, ****P < 0.0001 at days 40 and 50. Mean ratio ± SEM.Representative images of GINs subtype somatostatin (SST) expression co‐staining with mature neuron marker MAP2 in CTRL and sMDD GINs at day 65. Scale bar = 50 μm.The proportion of SST^+^ neurons of MAP2^+^ cells from CTRL and sMDD iPSCs derived GINs at day 65. *n* = 43 in CTRL groups and *n* = 37 in sMDD groups. *T*‐test, ****P < 0.0001. Mean ratio ± SEM. Source data are available online for this figure.

**Figure EV1:**
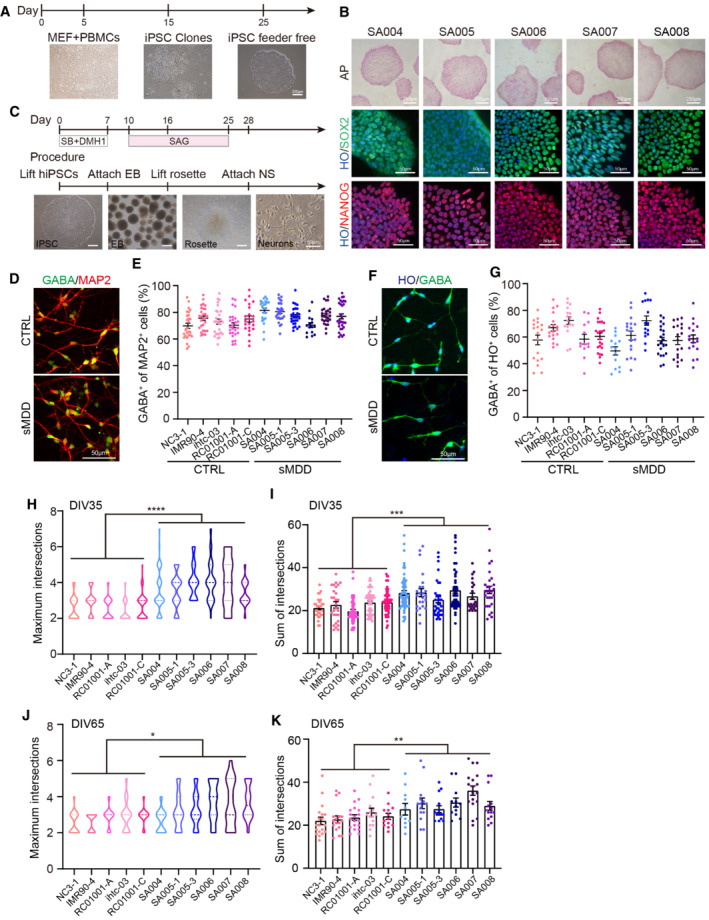
Generation of iPSCs and GABA interneurons from sMDD patients. *Related to* Fig 1 Scheme illustrating the generation of iPSCs from CTRL and sMDD patients' peripheral blood mononuclear cell (PBMC). Scale bar = 250 μm. (MEF, Mouse Embryonic Fibroblast).Alkaline phosphatase staining and SOX2, NANOG immunostaining in undifferentiated iPSCs from 5 sMDD patients. Scale bar shown in the images.Schematic diagram of iPSC differentiation to GINs. The left three pannels: scale bar = 250 μm. The right pannel: scale bar = 50 μm.Representative images of GABAergic interneurons with the staining of GABA and MAP2 from CTRL and sMDD groups. Scale bar = 50 μm.The proportion of GABA^+^ cells of MAP2^+^ neurons from five CTRL cell lines and six sMDD cell lines at day 35. *n* ≥ 15. Mean ratio ± SEM.Representative images of GABAergic interneurons with the staining of HO and GABA from CTRL and sMDD cell lines. Scale bar = 50 μm.The proportion of GABA^+^ neurons of HO^+^ cells from five CTRL cell lines and six sMDD cell lines at day 35. *n* ≥ 13.Mean ratio ± SEM.Quantification of maximum of intersections shown in five CTRL cell lines and six sMDD cell lines at day 35, *n* = 226 neurons from CTRL groups, *n* = 260 neurons from sMDD groups. Nested *t*‐test, ****P < 0.0001 for CTRL versus sMDD. The center line represents the median. Mean ratio ± SEM.Quantification of sum of intersections shown in five CTRL cell lines and six sMDD cell lines at day 35, *n* = 226 neurons from CTRL groups, *n* = 260 neurons from sMDD groups. Nested *t*‐test, ***P = 0.0006 for CTRL versus sMDD. Mean ratio ± SEM.Quantification of maximum of intersections by Sholl analysis shown in five CTRL cell lines and six sMDD cell lines at day 65, *n* = 83 neurons from both CTRL groups and sMDD groups. Nested *t*‐test, *P = 0.0118 for CTRL versus sMDD. The center line represents the median. Mean ratio ± SEM.Quantification of sum of intersections by Sholl analysis shown in five CTRL cell lines and six sMDD cell lines at day 65, *n* = 83 neurons from both CTRL groups and sMDD groups. Nested *t*‐test, **P = 0.0027 for CTRL versus sMDD. Mean ratio ± SEM. Source data are available online for this figure.

We reprogrammed peripheral blood cells to iPSCs from five sMDD patients who attempted suicide (SA004, SA005‐1, SA005‐3, SA006, SA007, and SA008. Notably, SA005‐1 and SA005‐3 are from the same subject). In parallel, we used NC3‐1, IMR90‐4, RC01001‐A, ihtc‐03, and RC01001‐C iPSC lines as controls (CTRLs, Figs 1A and EV1A, Table EV1). The established sMDD iPSC lines expressed pluripotency markers, including alkaline phosphatase (AP), SOX2, and NANOG (Fig EV1B). Following differentiation to GABAergic interneurons (Stefansson *et al*, 2003) using our established protocol (Liu *et al*, 2013; Shen *et al*, 2020) (Fig EV1C), both sMDD and CTRL iPSC lines generated > 80% GABA^+^ cells among TUJ1^+^ neurons at day 35 (Fig 1B and C). Also, we observed the proportion of GABA^+^ cells in MAP2^+^ mature neurons (ranging from 69.91 to 81.54%) and HO^+^ total cells (ranging from 49.63 to 72.43%) (Fig EV1D–G). There was no significant difference between different cell lines.

The stress‐induced depressive model showed a defect in neural morphology and dendritic density (Qiao *et al*, 2016; Zhao *et al*, 2018). We then examined the morphology of sMDD GINs at day 35 by using Sholl analysis (Fig 1D). The GINs in sMDD groups showed a significant increase in the intersection numbers with the concentric circles at the distance of 10, 30, and 50 μm from soma (Fig 1E), respectively. Also, the maximum intersections and the sum of interactions were elevated, compared with the CTRL groups (Fig EV1H and I), indicating increased neurite branches and neural complexity in the sMDD GINs. We next analyzed the morphology of mature GINs at day 65, which showed similar results in sMDD GINs (Fig 1F), including increased maximum intersections, and sum of intersections (Fig EV1J and K). Furthermore, we measured primary branch number and longest neurite length of GINs, which were both increased in sMDD groups (Fig 1G and H).

Previous studies suggested that the reduced density of GINs subtypes, such as calretinin (CR) and somatostatin (SST), may be related to MDD (Smiley *et al*, 2016; Song *et al*, 2021). To further analyze the GINs subtypes, we assessed CR expression by co‐staining for TUJ1 at days 35, 40, and 50, respectively. We found no significant difference between the sMDD and CTRL groups at day 35. However, at day 40, the number of CR neurons in the sMDD group (11.8 ± 0.4%) was significantly fewer than that in the CTRL groups (17.4 ± 0.7%). Furthermore, the decreased population of CR neurons was obtained at day 50 (sMDD: 16.0 ± 0.7%; CTRL: 28.3 ± 1.2%) (Fig 1I and J). At day 65, we found decreased population of SST^+^ cells in sMDD groups compared with CTRL groups (Fig 1K and L). Altogether, the results indicated that the proportion of GINs subtypes were altered in sMDD groups.

### sMDD GINs exhibit electrophysiological hyperexcitability

**Figure 2:**
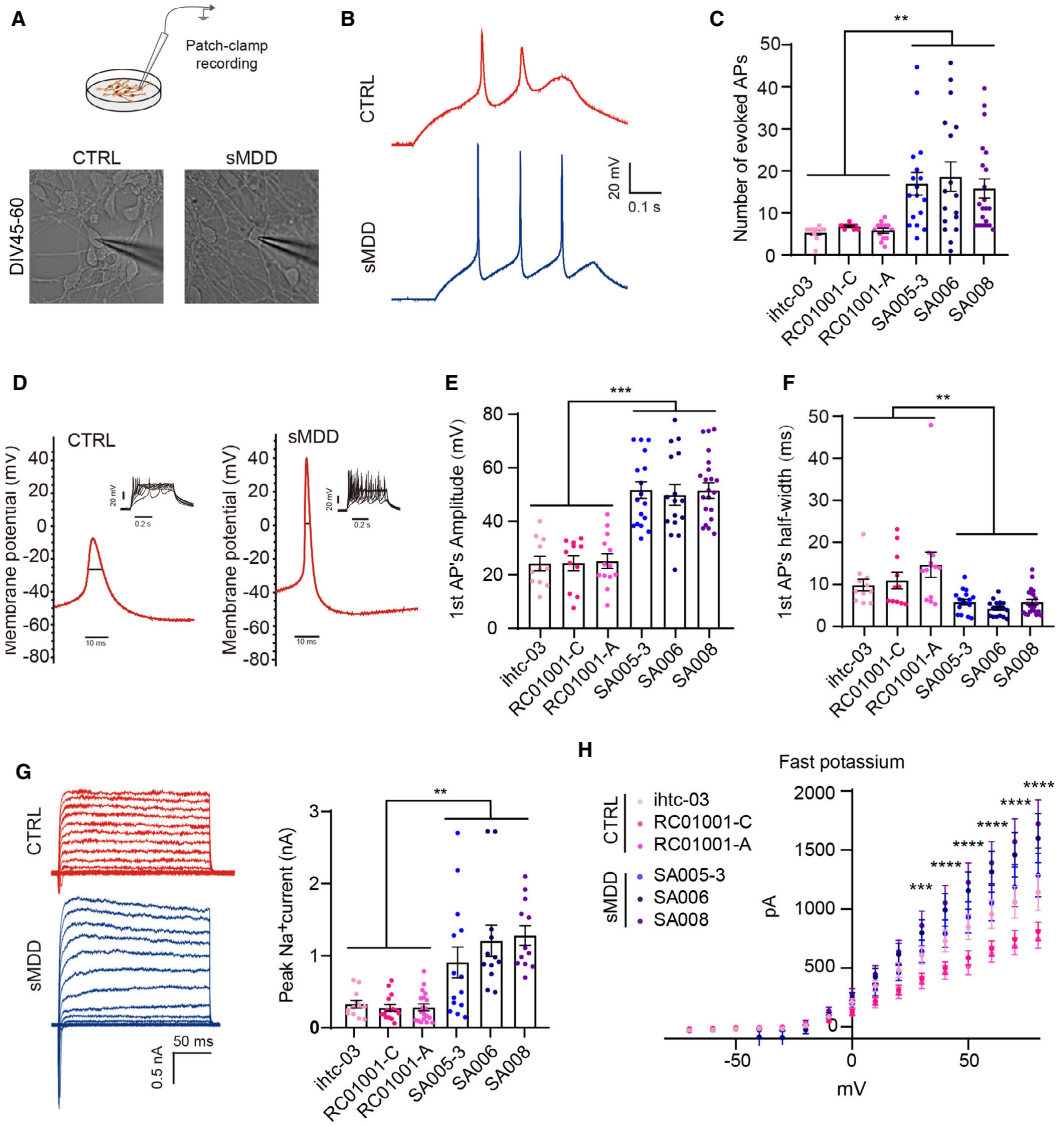
sMDD GINs show neural electrophysiological abnormalities at days 45–60 ASchematic diagram and representative images illustrating whole‐cell patch‐clamp recording.BRepresentative electrophysiological traces of AP at a holding potential of −70 mV from GINs in sMDD (blue) and CTRL (red) groups.CAverage number of APs evoked during 500 ms stepwise depolarization (CTRL, *n* = 35 neurons from three lines; sMDD, *n* = 55 neurons from three lines). Nested *t*‐test, **P < 0.01. Mean ratio ± SEM.D–FAmplitude and half width of the first AP generated in response to a 10‐pA injection (CTRL, *n* = 35 neurons from three lines; sMDD, *n* = 55 neurons from three lines). Nested *t*‐test, **P < 0.01; ***P < 0.001. Mean ratio ± SEM.GTraces of Na^+^/K^+^ currents recorded from GINs in sMDD and CTRL groups. Quantification showed average of peak values of Na^+^ currents (CTRL, *n* = 46 neurons from three lines; sMDD, *n* = 38 neurons from three lines). Nested *t*‐test, **P < 0.01.HAverage fast potassium currents of GINs in sMDD and CTRL groups (CTRL, *n* = 43 neurons from three lines; sMDD, *n* = 36 neurons from three lines). Two‐way ANOVA, ***P < 0.001; ****P < 0.0001 for CTRL versus sMDD. Mean ratio ± SEM. Source data are available online for this figure.

One of the hypothesized mechanisms of MDD is impaired neural circuits (Duman *et al*, 2019). We performed whole‐cell patch‐clamp recording on 6‐week‐old cultures to measure the intrinsic membrane properties of the GINs (Fig 2A). Under voltage clamp, APs were evoked by current injection at the range from 10 to 60 pA in GINs of both the CTRL and the sMDD groups. However, APs were more readily evoked in the sMDD GINs than the CTRLs. Also, the AP amplitude and frequency were higher in the sMDD GINs (Fig 2B–E). The half‐width of APs in sMDD GINs was ~56% narrower than that of CTRLs (Fig 2F), revealing excessive channel activation (Bean, 2007; Paşca *et al*, 2011).

The sodium and potassium currents correlate with APs. We found larger inward sodium currents in sMDD GINs (Fig 2G), which were activated in response to similar membrane depolarization as for CTRL. Furthermore, the amplitude of the fast potassium currents in the range of 30–80 mV was markedly elevated in the sMDD GINs (Fig 2H). Collectively, these data reveal the hyperexcitability of sMDD GINs.

**Figure EV2:**
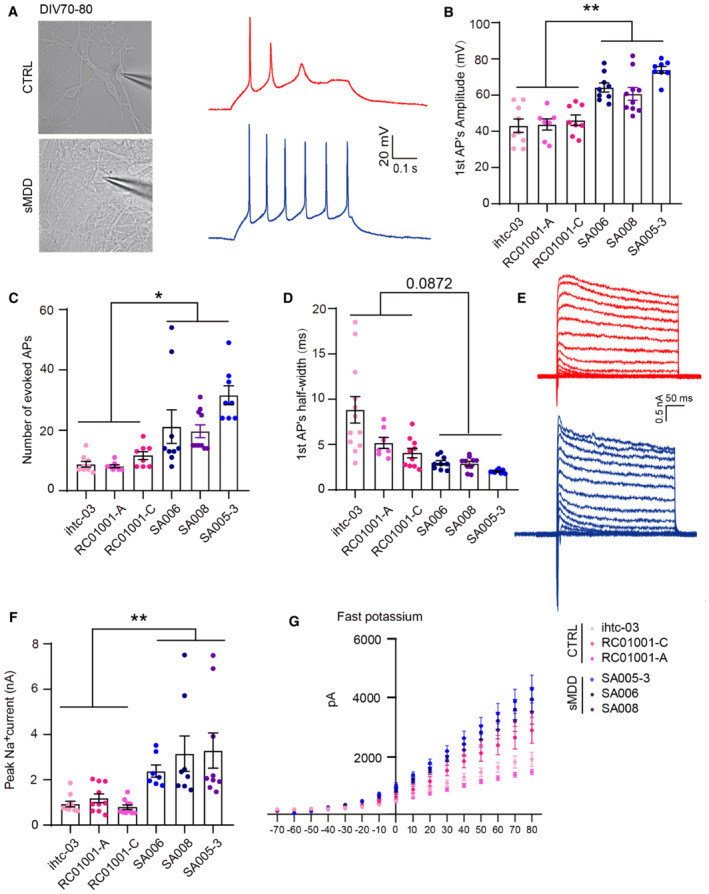
sMDD GINs show neural electrophysiological abnormalities at days 70–80. *Related to* Fig 2 ASchematic diagram and representative images illustrating the method for whole‐cell patch‐clamp recording. Representative electrophysiological traces of AP at a holding potential of −70 mV from GINs in sMDD and CTRL groups.BAmplitude of first AP generated in response to a 10‐pA injection (CTRL, *n* = 23 neurons from three lines; sMDD, *n* = 25 neurons from three lines). Nested *t*‐test, **P < 0.01. Mean ratio ± SEM.CAverage total number of APs evoked during 500 ms stepwise depolarization (CTRL, *n* = 23 neurons from three lines; sMDD, *n* = 25 neurons from three lines). Nested *t*‐test, *P < 0.05. Mean ratio ± SEM.DHalf width of first AP generated in response to a 10‐pA injection (CTRL, *n* = 23 neurons from three lines; sMDD, *n* = 25 neurons from three lines). Nested *t*‐test, *P* = 0.0872. Mean ratio ± SEM.E, FSample traces of Na^+^/K^+^ currents recorded from GINs in sMDD and CTRL groups. Average peak values of Na^+^ currents (CTRL, *n* = 30 neurons from three lines; sMDD, *n* = 24 neurons from three lines). Nested *t*‐test, **P < 0.01. Mean ratio ± SEM.GAverage fast potassium currents of GINs in sMDD and CTRL groups (CTRL, *n* = 30 neurons from three lines; sMDD, *n* = 24 neurons from three lines). Mean ratio ± SEM. Source data are available online for this figure.

To further determine the electrophysiological characteristics of sMDD GINs at later mature stage, we performed patch‐clamp recording at day 70. Consistent with our results at day 45, sMDD GINs showed increased amplitudes and evoked numbers of APs (Fig EV2A–D), along with larger potassium and sodium currents (Fig EV2E–G). These results demonstrated electrophysiological hyperexcitability in the sMDD GINs.

### sMDD GINs and ventral forebrain organoids display altered calcium signaling

**Figure 3:**
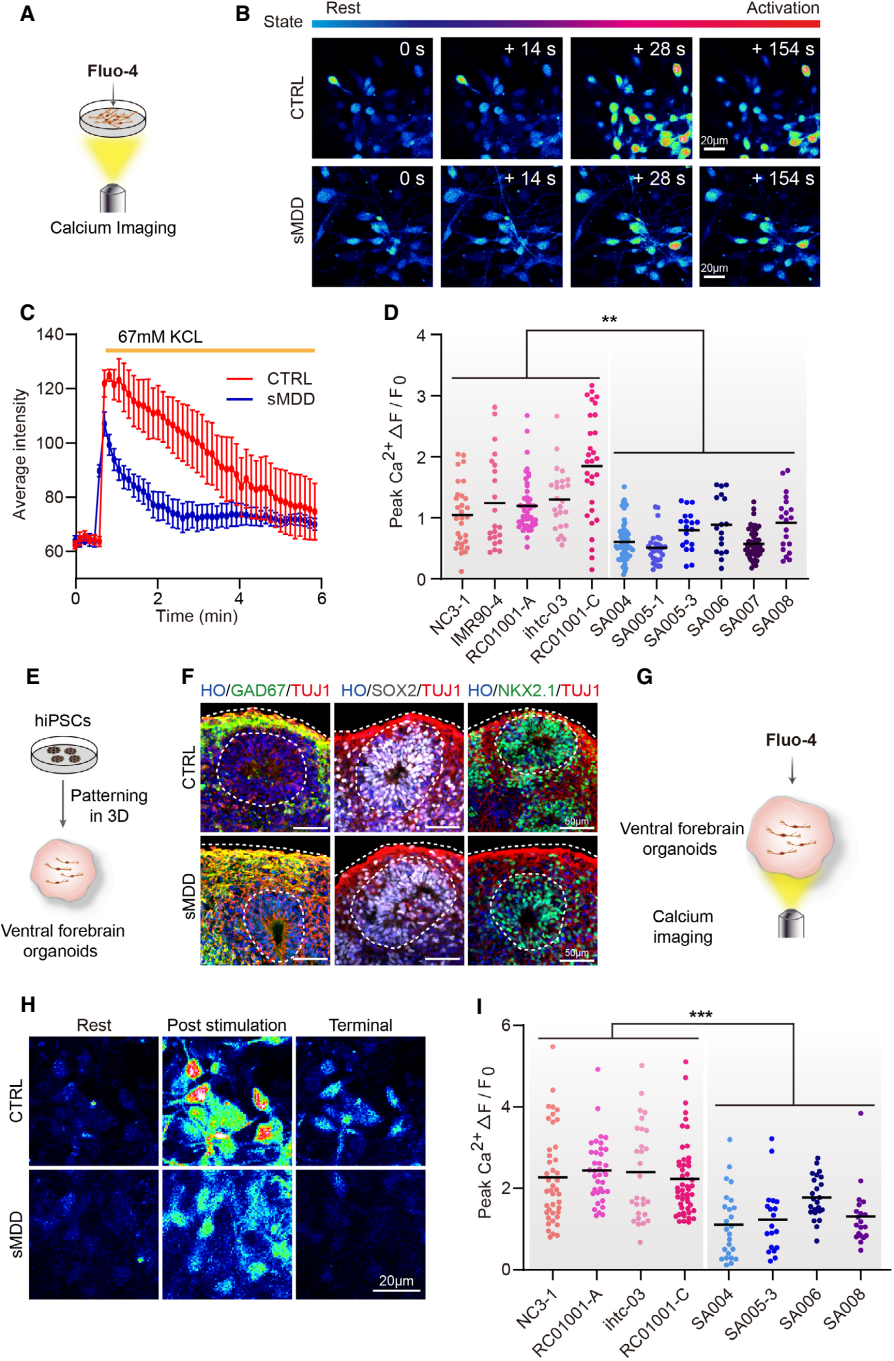
Defects in calcium signaling both in sMDD GINs and ventral forebrain organoids Schematic diagram of calcium imaging for neurons with Fluo‐4 indicator dyes.Representative images of calcium imaging in CTRL and sMDD GINs at each timepoint. Scale bar = 20 μm.The representative trajectory of average intensity changes over time from CTRL (red line) and sMDD (blue line) neurons in response to 67 mM KCL. CTRL, *n* = 6 neurons; sMDD, *n* = 6 neurons. Traces are from a representative experiment (the whole quantification result is shown in D). Mean ratio ± SEM.Quantification of peak [Ca^2+^] (*F*_max_–*F*_0_)/*F*_0_ shown per cell line (*n* = 155 neurons derived from five CTRL cell lines, *n* = 198 neurons derived from six sMDD cell lines). Nested *t*‐test, **P = 0.0022 for CTRL versus sMDD. Mean ratio ± SEM.Schematic illustrating the generation of human ventral forebrain organoids from hiPS cells.Immunostaining for the GABA interneuron marker GAD67, neural progenitor marker SOX2 and ventral prosencephalic progenitor marker NKX2.1 at day 30 in CTRL and sMDD ventral forebrain organoids. Scale bar = 50 μm.Schematic diagram of calcium imaging for ventral forebrain organoids with Fluo‐4 indicator dyes.Representative images of calcium imaging in CTRL and sMDD ventral forebrain organoids at different states. Scale bar = 20 μm.Quantification of peak [Ca^2+^] (*F*_max_–*F*_0_)/*F*_0_ shown per cell line (*n* = 156 neurons derived from four CTRL cell lines, *n* = 88 neurons derived from four sMDD cell lines). Nested *t*‐test, ***P = 0.0005 for CTRL versus sMDD. Mean ratio ± SEM. Source data are available online for this figure.

Calcium imaging has been reported as a reliable method of studying neural circuits (de Melo Reis *et al*, 2020). In rodent studies, disturbance of Ca^2+^ homeostasis or fewer calcium signals were observed in MDD group (Ma *et al*, 2019; Wang *et al*, 2021). Here, to investigate whether the abnormal neuronal hyperexcitability in sMDD GINs is coupled with changes in calcium signaling during depolarization, GINs were assessed at day 40 via fluorescence imaging with the calcium indicator dye Fluo‐4 AM and time‐series microscopy (Fig 3A). After Fluo‐4 AM was loaded onto the cells, intracellular calcium fluctuations were monitored via fluorescent intensity over time with 67 mM KCL stimulation (Fig 3B). Then we observed a decrease in the amplitude of [Ca^2+^]_i_ rise following KCL stimulation after 1‐min continuous imaging (Fig 3C). Next, we performed further statistical analysis on the average intensity curve. We found the value of Peak Ca^2+^(*F*_max_–*F*_0_)/*F*_0_, which indicated the amplitude of calcium signaling (Wen *et al*, 2013), was lower in sMDD GINs from five cell lines compared with six CTRL cell lines (Fig 3D).

Brain organoid is considered as an effective human model for studying neurological diseases (Amin & Pasca, 2018; Manley & Anderson, 2019; Xu *et al*, 2019; Kang *et al*, 2021). We generated the ventral forebrain organoids, which expressed GAD67, neural progenitor marker SOX2, and ventral prosencephalic progenitor marker NKX2.1 at day 30 (Fig 3E–G), using our established method (Yuan *et al*, 2015, 2020). Also, we performed calcium imaging in our ventral forebrain organoids. Series images of partial organoids showed that the amplitude of [Ca^2+^]_i_ rise in sMDD organoids significantly decreased compared with CTRL ventral organoids (Fig 3H and I). Taken together, these data from both 2D neurons and 3D organoids demonstrated the dysfunction of calcium signaling transmission in sMDD GINs.

### Transcriptional analysis of sMDD GINs reveals defected expression of *HTR2C*

**Figure EV3:**
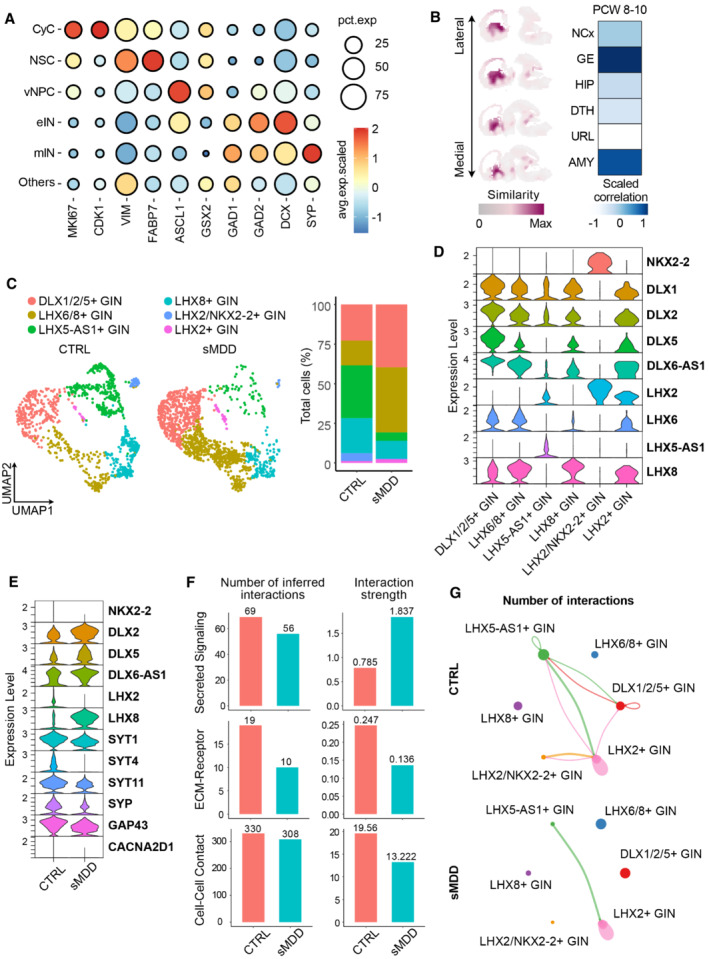
Analysis of RNA‐seq and DEGs. *Related to* Fig 4 Dot plot showing the expression level and percentage of representative marker genes across the six main cell types. The mRNA level of each gene is shown on the linear scale and normalized for different groups.VoxHunt spatial mapping of our single cell transcriptome onto data from E13.5 mouse brains, which is available in the Allen Brain database. Sagittal sections are shown and colored by scaled similarity scores.Left panel: UMAP visualization of GABAergic neuron subclusters, split by groups. Right panel: Stacked bar chart showing the composition of different GINs for each sample, after 35 days of differentiation.Stacked violin plot showing the expression level of ventral transcription factors across different types of GINs.Stacked violin plot showing the genes differentially expressed in CTRL and sMDD GINs.Bar plots showing the number of inferred interactions and interaction strength for ligand‐receptor pairs in CTRL and sMDD GINs, which could be classified into three categories, including Secreted Signaling, ECM‐Receptor, and Cell–Cell Contact.Cell communication networks showing the number of interactions related to ECM‐receptor in CTRL and sMDD GIN subclusters.

**Figure 4:**
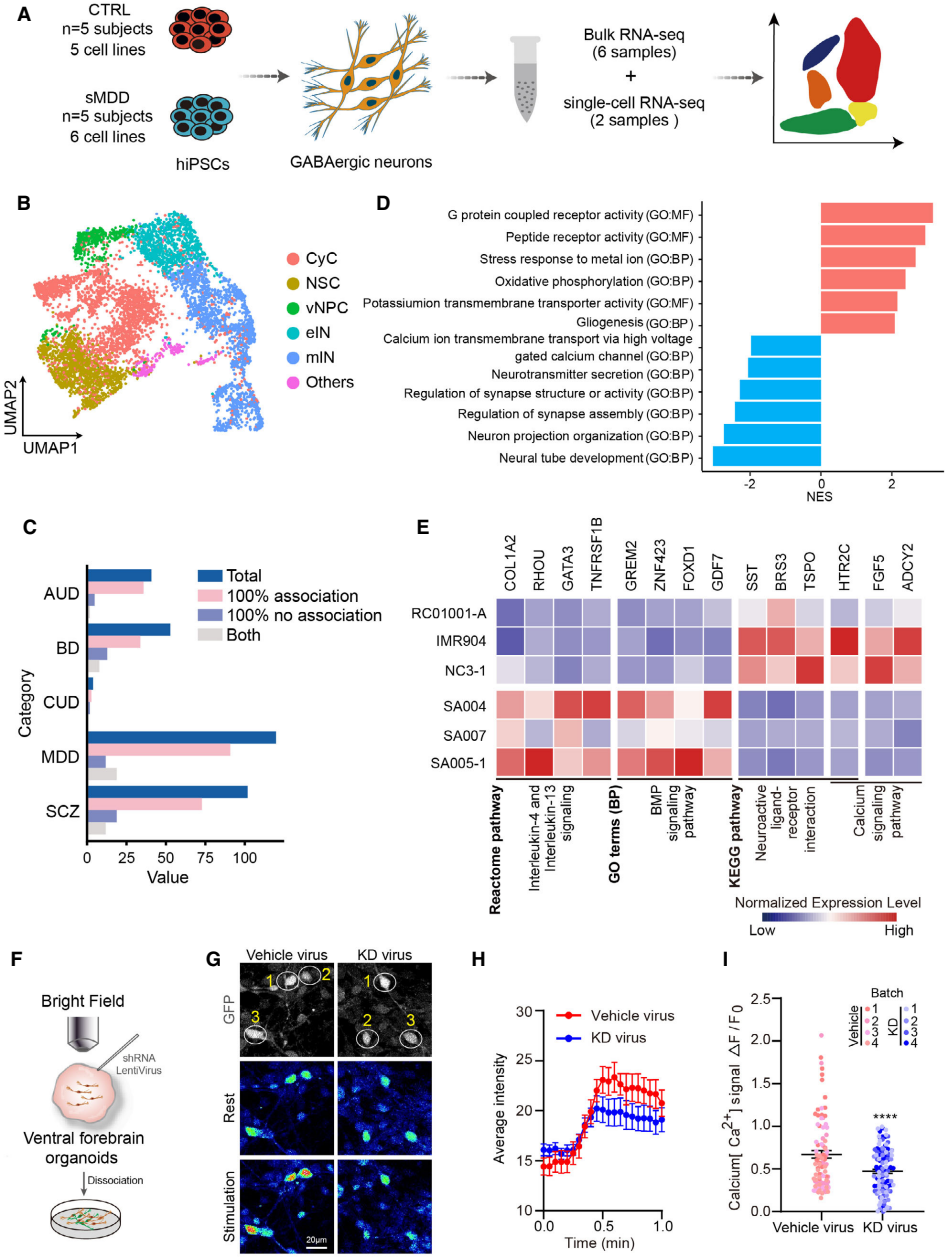
Analysis of scRNA‐seq and bulk RNA‐seq identified abnormal expression of *HTR2C* Schematic representation of transcriptomic sequencing experiments for GABAergic neurons derived from CTRL and sMDD iPSCs.UMAP plot showing the cell types detected in CTRL and sMDD samples. CyC, cycling cells; NSC, neuronal stem cells; vNPC, ventral neuronal progenitor cells; eIN, early interneurons; mIN, mature interneurons.Bar plot showing the number of genes associated with known mental illnesses, based on PsyGeNET. “Total” presents all of the genes related to a given disease. “100% association” presents the genes only positively correlated with the disease. “100% no association” presents the genes only negatively correlated with the disease. “Both” means genes that both positive and negative results were found. AUD, BD, CUD, MDD, and SCZ represent alcohol use disorder, bipolar disorder, cocaine use disorder, major depressive disorder, and schizophrenia, respectively.Gene set enrichment analysis (GSEA) for GIN clusters, which was based on Ontology gene sets of differentially expressed genes (DEGs). Presented GO terms are all significantly changed (adjusted *P*‐value < 0.05).Heatmap illustrating the expression level of DEGs enriched in selected Reactome pathways, gene ontology (GO) terms, and Kyoto Encyclopedia of Genes and Genomes (KEGG) pathways, which is normalized by row.Schematic diagram of injecting knockdown virus into ventral forebrain organoids followed by neurons adherent.Calcium imaging was performed on GFP^+^ neurons in the knockdown group. The representative image shows the rest state of GFP^+^ cells and the state of cells after KCl stimulation. The pseudo‐color showed Rhod‐4 calcium fluorescent dye, and the selected cells are GFP^+^ neurons. Scale bar = 20 μm.The representative trajectory of average intensity changes over time in Vehicle virus and KD virus groups. CTRL, *n* = 10 neurons; sMDD, *n* = 10 neurons. Traces are from a representative experiment (the whole quantification result is shown in I). Mean ratio ± SEM.Quantification of peak [Ca^2+^] (*F*_max_–*F*_0_)/*F*_0_ shown per virus group. *n* = 89 for Vehicle virus, *n* = 118 for KD virus, respectively from four batches. *T*‐test, ****P < 0.0001. Mean ratio ± SEM. Source data are available online for this figure.

To detect the changes in transcriptome profile, single‐cell RNA sequencing (scRNA‐seq) was performed on the NC3‐1 and SA005‐1 cell lines with over 3,000 cells from each sample (Fig 4A). Uniform Manifold Approximation and Projection (UMAP) analysis identified five main cell types according to the expression of known cell‐type markers, including cycling cells (CyC), neural stem cells (Rentería *et al*, 2017), ventral neuronal progenitor cells (vNPC), early interneurons (eIN), and mature interneurons (mIN) (Figs 4B and EV3A). Mapping of the scRNA‐seq transcriptome onto the Allen Brain Atlas showed similarity of our GINs to the ventral forebrain of the E13.5 mouse brain (Fig EV3B). Moreover, comparison to the BrainSpan database (postconceptional weeks (PCW) 8–12) revealed the highest correlation to the ganglionic eminences (GE) region (Fig EV3B). Hence, our *in vitro* generated cells are primarily MGE‐derived GINs and their progenitors.

We next performed differential expression analysis on CTRL and sMDD samples. The genes differentially expressed in sMDD group shared the most associations with MDD, showing our cellular model recapitulating the transcriptomic aspect of depressive disorders and schizophrenia based on the publicly available mental illness database PsyGeNET (Gutierrez‐Sacristan *et al*, 2015) (Fig 4C). Gene ontology analysis, which applied gene set enrichment analysis (GSEA) method, revealed altered biological processes and molecular functions in sMDD GIN clusters (Fig 4D). The terms of GABAergic neuron differentiation and potassium channel activity were upregulated, while the term of high voltage‐gated calcium channel activity was downregulated, suggesting the altered calcium channel function in sMDD GINs (Fig 4D).

To further detect subtype signatures of sMDD GINs, we especially extracted all GINs cells and repeated clustering procedure. UMAP visualization of subclusters in eIN and mIN cell types showed six clusters of GINs according to unbiased clustering algorithm, and relative proportions of subclusters across CTRL and sMDD samples were also illustrated (Fig EV3C and D). Subclusters were defined according to each highly expressed transcription factors related to GIN development (Fig EV3C and D). Although there were more neurons highly expressing both *LHX6* and *LHX8* in sMDD (Fig EV3C), the expression of genes reflecting synaptic maturation and plasticity, as well as response to calcium, such as *SYT1*, *SYT4*, *SYT11*, *SYP*, *GAP43*, was downregulated (Fig EV3E), which was consistent with our previous observations of impaired GINs function in sMDD group.

To predict how GINs work together to coordinate physiological activities both in CTRL and sMDD samples, we used CellChat (Jin *et al*, 2021) program to compare the number and strength of interactions among different GIN subclusters in these two groups. Notably, GINs in the sMDD group displayed decreased number of interaction and lower level of connectivity for almost each type of ligand–receptor interactions, especially in interactions related to ECM‐Receptor, which indicated that the ability of sMDD GINs to perceive and respond to extracellular matrix, such as calcium, growth factors, and bioactive molecules, was impaired (Fig EV3F and G).

**Figure EV4:**
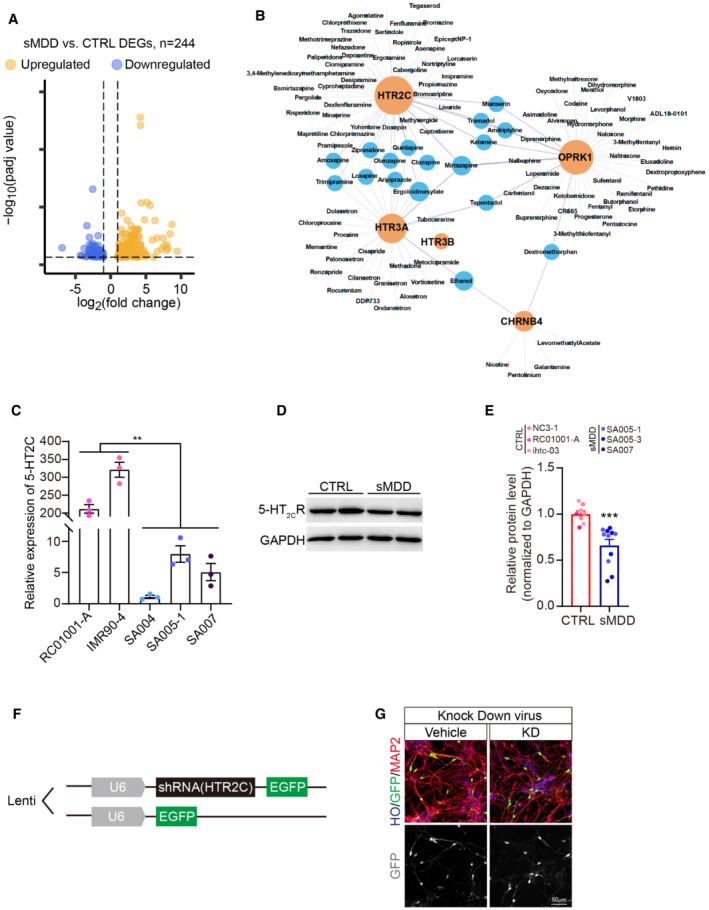
Validation of *HTR2C* downregulating in sMDD. *Related to* Fig 4 AVolcano plot showing all differentially expressed genes between three CTRL and three sMDD samples in bulk RNA‐seq.BNetwork maps highlighting genes from DEGs with the most targeted molecules according to the Drug Bank database. Yellow dots represent genes, and the blue dots are drugs related to two genes.CRelative expression levels of 5‐HT2CR at days 35–40 from two CTRL cell lines and three sMDD cell lines using qPCR. *n* = 3 from each cell line. Nested *t*‐test, **P = 0.0077. Mean ratio ± SEM.D, EWestern blotting analysis and quantification of 5‐HT2CR expression in GINs at days 35–40 from three CTRL cell lines and three sMDD cell lines. *T*‐test, ***P = 0.0007 for CTRL versus sMDD. CTRL, *n* = 8 from three cell lines; sMDD, *n* = 11 from three cell lines. Mean ratio ± SEM.FSchematic diagram of knockdown lentivirus sequence.GRepresentative images of shRNA knockdown virus‐infected neurons. Scale bar = 50 μm. Source data are available online for this figure.

To further clarify the accuracy of single‐cell analysis results and to better detect the expression of genes that encode low‐abundance receptors, we performed bulk‐seq analysis on three CTRL lines and three sMDD lines after 5 weeks of differentiation. We determined DEGs between the CTRL and sMDD groups by using the DESeq2 (Love *et al*, 2014) package. There were 244 DEGs between these two groups, among which 65 genes were downregulated, and 179 genes were upregulated (Fig EV4A). Gene ontology (GO) enrichment of biological process showed that the term of BMP signaling pathway was upregulated in sMDD samples. Interleukin‐4 and Interleukin‐13 signaling was also enhanced in sMDD according to the results of Reactome pathway analysis, which associated with neuronal hyperactivity (Sun *et al*, 2007; Thürmann *et al*, 2019). Kyoto Encyclopedia of Genes and Genomes (KEGG) pathway analysis indicated that pathways including neuroactive ligand–receptor interaction and calcium signaling pathway were decreased, which were consistent with scRNA‐seq results (Fig 4E). Among these gene enrichment analyses, we found that *HTR2C* was contained in many pathways. By combining the DEGs and their target drugs in DrugBank (Wishart *et al*, 2006) to form a gene–drug network and ranking the potential targets of the drug based on their proximity to the drug‐induced expression, we discovered that *HTR2C* was the most enriched gene in DEGs, which encodes 5‐HT2CR protein in human (Fig EV4B).

According to the transcriptome sequencing results showing that the expression of *HTR2C* was downregulated in the sMDD group. First, we verified the decreased expression of 5‐HT_2C_R at the RNA and protein levels in the sMDD group by quantitative PCR and Western blotting (Fig EV4C–E). Next, to further confirm the role of *HTR2C* in mediating calcium deficiency in sMDD and to assess whether the phenotype is recapitulative, we infected shRNA lentivirus into RC01001‐C (CTRL cell line) ventral forebrain organoids with U6 as the promoter (pCLenti:U6:shRNA(*HTR2C*)), which specifically knocked down *HTR2C* to mimic the disease phenotype (Fig EV4F). We injected virus to the cerebral organoids in the brightfield and digested them into single cells for adherent culture 7 days after infection (Figs 4F and EV4G). The adherent neurons were then analyzed for calcium imaging by using a Rhod‐4 calcium imaging kit, which allows us to observe the calcium signaling on GFP^+^ neurons in 546 channel. We first performed imaging analysis on the adherent neurons in the CTRL group and observed the calcium signaling over the GFP^+^ neurons (Fig 4G) and used KCl for depolarization stimulation. The results showed the calcium signaling of the neurons in the KD virus group was significantly lower than that of the vehicle virus group (Fig 4H and I), indicating that downregulation of *HTR2C* in CTRL group showed sMDD‐associated pathological phenotype with decreased calcium signaling.

### Upregulation of 5‐HT2C receptor restores abnormal GABAergic neuronal activity

**Figure 5:**
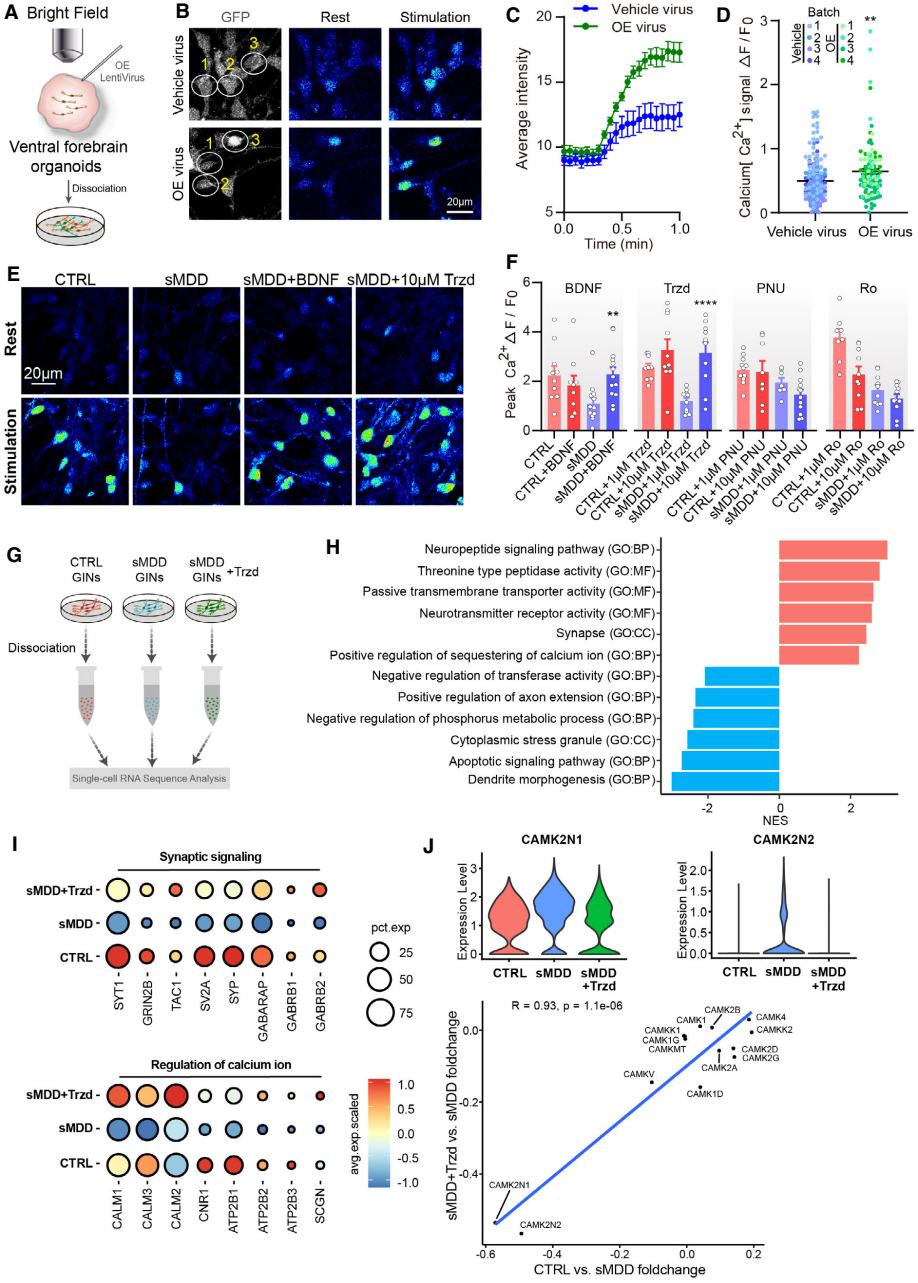
Overexpression of *HTR2C* and Trzd partially reverses defects of sMDD GINs in single‐cell transcriptome level Schematic diagram of injecting overexpression virus into ventral forebrain organoids followed by neurons adherent.Calcium imaging was performed on GFP^+^ neurons in the overexpression group. The representative image shows the rest state of GFP^+^ cells and the state of cells after KCl stimulation. The pseudo‐color showed Rhod‐4 calcium fluorescent dye, and the selected cells are GFP^+^ neurons. Scale bar = 20 μm.The representative trajectory of average intensity changes over time in Vehicle virus and OE virus groups. CTRL, *n* = 10 neurons; sMDD, *n* = 10 neurons. Traces are from a representative experiment (the whole quantification result is shown in D). Mean ratio ± SEM.Quantification of peak [Ca^2+^] (*F*_max_–*F*_0_)/*F*_0_ shown per virus group. *n* = 166 for Vehicle virus, *n* = 81 for OE virus, respectively from three batches. *T*‐test, **P = 0.0012. Mean ratio ± SEM.Representative images of calcium imaging in CTRL (from RC01001‐C) and sMDD (from SA004) GINs at resting and stimulus state with different treatments. Scale bar = 20 μm.Quantification of peak [Ca^2+^] (*F*_max_–*F*_0_)/*F*_0_ shown with different treatments including BDNF, Trzd, PNU, Ro at concentrations of 1 and 10 μM. *T*‐test, **P = 0.0042 for sMDD + BDNF versus sMDD; one‐way ANOVA, ****P < 0.0001 for sMDD + 10 μM Trzd versus sMDD. *n* ≥ 7 neurons from each group. Mean ratio ± SEM.Schematic diagram of single cell sequencing from CTRL, sMDD, and sMDD + 10 μM Trzd GINs followed by assaying.Gene set enrichment analysis (GSEA) results indicating altered GO terms after Trzd treatment (adjusted *P*‐value < 0.05). Upregulated terms are colored with red while downregulated terms are colored with blue.Dot plots showing the expression level and percentage of genes selected from functional gene sets across CTRL, sMDD, and sMDD with Trzd GINs clusters.Top: Violin plots showing the expression level of *CAMK2N1* and *CAMK2N2* in three groups. Bottom: Scatter plot showing the correlations of foldchanges according to the expression of genes encoding CaMK family proteins between CTRL GINs and sMDD GINs and between sMDD with Trzd GINs and sMDD GINs. A linear regression line is added to the plot based on these two variables. sMDD + Trzd vs. sMDD, *n* = 14 genes; CTRL vs. sMDD, *n* = 14 genes. Source data are available online for this figure.

**Figure EV5:**
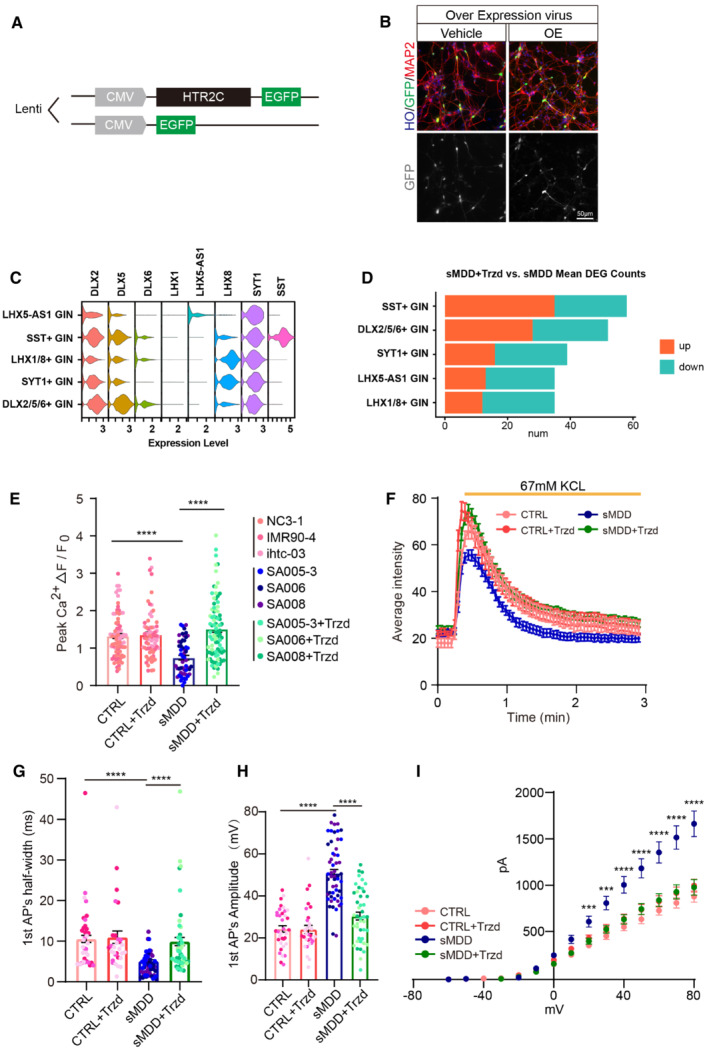
Restoration of neural function after overexpression of *HTR2C* and treatment of Trzd. *Related to* Fig 6 Schematic diagram of overexpression lentivirus sequence.Representative images of overexpression virus‐infected neurons. Scale bar = 50 μm.Stacked violin plot showing the expression of representative cell type makers across different GIN subclusters.Mean counts of differently expressed genes (DEGs), which are calculated from downsampled data of different GIN subclusters. Mean counts of upregulated genes and downregulated genes are shown in red orange and turquoise respectively.Quantification of peak [Ca^2+^] (*F*_max_–*F*_0_)/*F*_0_ shown per cell line (CTRL, *n* = 79 neurons; CTRL + Trzd, *n* = 70 neurons; sMDD, *n* = 54 neurons; sMDD + Trzd, *n* = 87 neurons). One‐way ANONA, ****P < 0.0001 for CTRL versus sMDD, ****P < 0.0001 for sMDD versus sMDD + Trzd. Mean ratio ± SEM.The representative trajectory of average intensity changes over time from CTRL, CTRL + Trzd, sMDD, and sMDD + Trzd ventral forebrain organoids in response to 67 mM KCL. CRTL, *n* = 29 neurons; CTRL + Trzd, *n* = 31 neurons; sMDD, *n* = 35 neurons; sMDD + Trzd, *n* = 43 neurons. Traces are from a representative experiment (the whole quantitative result is shown in Fig 6J). Mean ratio ± SEM.Average half width of first AP generated in response to a 10‐pA injection (CTRL, *n* = 35 neurons from three lines; CTRL + Trzd, *n* = 30 neurons from three lines; sMDD, *n* = 55 neurons from three lines; sMDD + Trzd, *n* = 44 neurons from three lines). One‐way ANONA, ****P < 0.0001. Mean ratio ± SEM.Amplitude first AP generated in response to a 10‐pA injection (CTRL, *n* = 35 neurons from three lines; CTRL + Trzd, *n* = 30 neurons from three lines; sMDD, *n* = 55 neurons from three lines; sMDD + Trzd, *n* = 44 neurons from three lines). One‐way ANONA, ****P < 0.00001. Mean ratio ± SEM.Average fast potassium currents of GINs in sMDD and CTRL groups (CTRL, *n* = 43 neurons from three lines; CTRL + Trzd, *n* = 34 neurons; sMDD, *n* = 36 neurons from three lines; sMDD + Trzd, *n* = 34 neurons from three lines). Two‐way ANONA, ***P < 0.001；****P < 0.0001, sMDD versus sMDD + Trzd. Mean ratio ± SEM. Source data are available online for this figure.

According to transcriptome sequencing results showing decreased expression of *HTR2C* in sMDD‐derived GINs, this led us to hypothesize the modulation of *HTR2C* in GINs, by genetic and pharmacological approaches. First, we injected *HTR2C* overexpression virus with CMV as the promoter (pSLenti:CMV:*HTR2C*) to SA004 (sMDD cell line) organoids to explore whether overexpression of *HTR2C* could rescue the sMDD calcium signaling defect phenotype (Fig EV5A). Likewise, we injected virus to ventral forebrain organoids in brightfield and adherent cultures for 7 days (Figs 5A and EV5B). Then, calcium imaging was performed on the adherent GINs to determine whether calcium signaling could be improved (Fig 5B). The results showed that after restoring the expression of *HTR2C* by lentivirus, the defected calcium signaling function in the sMDD group was significantly reversed (Fig 5C and D).

Next, we tried to rescue the phenotypes by pharmacological approaches. FDA‐approved drugs/small molecular 5‐HT_2C_R agonists, PNU‐22394 (PNU), Ro60‐0175 (Ro), and Trazodone hydrochloride (Trzd), were used to test their effects on calcium signaling and electrophysiological properties in sMDD GINs. Cells were treated with two concentrations (1 and 10 μM) of each drug, and BDNF was used as a positive CTRL. Calcium imaging experiments were carried out to monitor the change of calcium signal fluctuations. The results showed that 10 μM Trzd significantly increased the calcium signaling peak (Fig 5E and F). Therefore, we selected it for further validation.

To assess whether Trzd could reverse the transcriptomic impairment in sMDD GINs, we performed single‐cell RNA sequencing on sMDD GABAergic neuronal cultures with treatment of Trzd for 10 days (Fig 5G). Using GSEA method, we observed terms related to neurotransmitter receptor activity and positive regulation of sequestering of calcium ion were upregulated in GIN clusters. Meanwhile, we found enrichment for genes related to cytoplasmic stress granule and dendrite morphogenesis, which were downregulated after adding Trzd (Fig 5H). To explore the cell‐type‐specific effect of Trzd on GINs, we extracted all cells labeled as GIN from an integrated single‐cell transcriptome and classified them into five clusters according to their highly expressed gene markers (Fig EV5C). The classification of GINs prompted us to investigate which GIN subtype could be affected by the treatment of Trzd. After performing multiple differentially expressed gene (DEG) analyses on downsampled data from each GIN subclusters, we found that SST positive GINs had the maximum number of DEGs compared with other GIN subclusters (Fig EV5D), suggesting that SST‐positive GINs in sMDD show the highest drug response to Trzd. To further test the reversal effect of Trzd, we checked the expression of representative genes related to synaptic signaling and regulation of calcium ion (Fig 5I). The results indicated that the expression of genes involved in these terms was reversed to the similar level of CTRL GINs after the treatment of Trzd, which was downregulated in sMDD group. Previous data in GSEA (Fig 5H) suggested that genes related to negative regulation of transferase activity were downregulated with the treatment of Trzd, in which we found two calcium‐dependent protein kinase (CaMK) encoding genes, CAMK2N1 and CAMK2N2. Then we compared the expression of genes encoding CaMK proteins across GINs of three groups. Notably, expression levels of genes encoding CaMKII inhibitor were similar in CTRL GINs and Trzd‐treated sMDD GINs, which upregulated in sMDD GINs (Fig 5J). Moreover, linear regression result of transcriptomic changes among three groups showed there was a highly positive correlation in the changes of expression between CTRL and Trzd‐treated sMDD with respect to untreated sMDD, indicating successful restoration of the expression of CaMK encoding genes (Fig 5J). Taken together, these results indicated that the impairment of transcriptome in sMDD GINs could be partly rescued with the treatment of Trzd.

**Figure 6:**
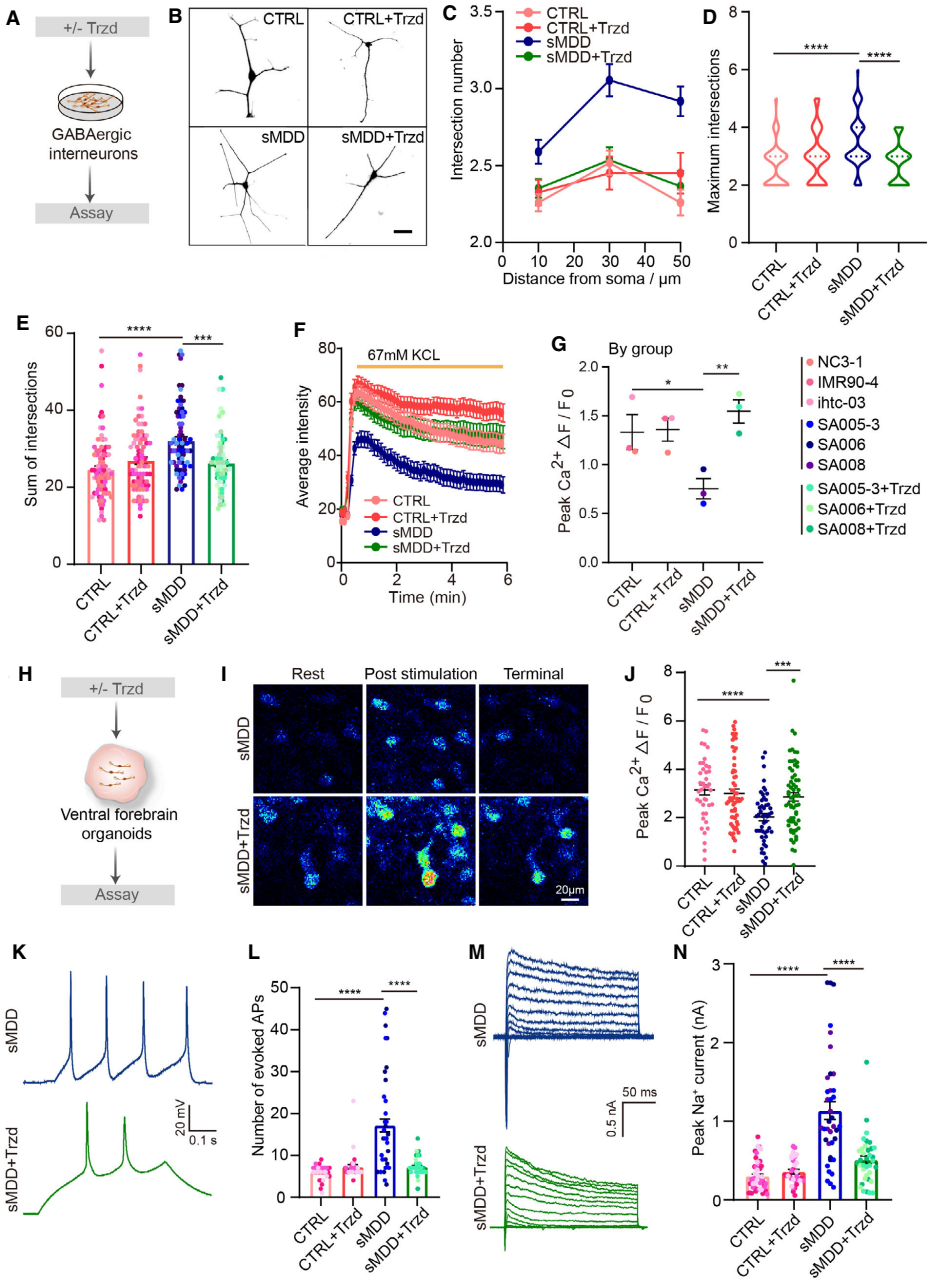
Trzd restores neuronal defects in sMDD GINs and ventral forebrain organoids Schematic diagram of treating GINs with 10 μM Trzd followed by assaying.Representative images of single neuron from CTRL, CTRL + Trzd, sMDD, and sMDD + Trzd GINs. Scale bar = 25 μm.Representative line chart of Sholl intersection number over distance from soma in CTRL, CTRL + Trzd, sMDD, and sMDD + Trzd GINs at day 45. CTRL, *n* = 81 neurons from five cell lines; CTRL + Trzd, *n* = 81 neurons from five cell lines; sMDD, *n* = 73 neurons from six cell lines; sMDD + Trzd, *n* = 71 neurons from six cell lines. Mean ratio ± SEM.Quantification of maximum intersections from CTRL, CTRL + Trzd, sMDD, and sMDD + Trzd GINs via Sholl analysis. CTRL, *n* = 81 neurons from 5 cell lines; CTRL + Trzd, *n* = 81 neurons from five cell lines; sMDD, *n* = 73 neurons from six cell lines; sMDD + Trzd, *n* = 71 neurons from six cell lines. One‐way ANONA, ****P < 0.0001 for CTRL versus sMDD and sMDD versus sMDD + Trzd.Quantification of sum of intersections from CTRL, CTRL + Trzd, sMDD, and sMDD + Trzd GINs via Sholl analysis. CTRL, *n* = 81 neurons from five cell lines; CTRL + Trzd, *n* = 81 neurons from five cell lines; sMDD, *n* = 73 neurons from six cell lines; sMDD + Trzd, *n* = 71 neurons from six cell lines. One‐way ANONA, ****P < 0.0001 for CTRL versus sMDD; ***P = 0.0002 for sMDD versus sMDD + Trzd. Mean ratio ± SEM.The representative trajectory of average intensity changes over time from CTRL, CTRL + Trzd, sMDD, and sMDD + Trzd GINs in response to 67 mM KCL. CTRL, *n* = 20 neurons; CTRL + Trzd, *n* = 20 neurons; sMDD, *n* = 20 neurons; sMDD + Trzd, *n* = 21 neurons. Tracs are from a representative experiment (the whole quantification result is shown in Fig EV5E). Mean ratio ± SEM.Quantification of peak [Ca^2+^] (*F*_max_–*F*_0_)/*F*_0_ shown per group, CTRL, CTRL + Trzd, sMDD, and sMDD + Trzd all from three cell lines. *T*‐test, *P = 0.0495 for CTRL versus sMDD, **P = 0.0075 for sMDD versus sMDD + Trzd. Mean ratio ± SEM.Schematic diagram of treating ventral forebrain organoids with 10 μM Trzd followed by assaying.Representative images of calcium imaging in sMDD and sMDD + Trzd ventral forebrain organoids at different states. Scale bar = 20 μm.Quantification of the effect of Trzd on peak [Ca^2+^] (*F*_max_–*F*_0_)/*F*_0_ in ventral forebrain organoids (CTRL from IMR90‐4, *n* = 40 cells; CTRL + Trzd from IMR90‐4, *n* = 58 cells; sMDD from SA007, *n* = 49 cells; sMDD + Trzd from SA007, *n* = 64 cells). Two‐tailed test, ****P < 0.0001 for CTRL versus sMDD; ***P = 0.0007 for sMDD versus sMDD + Trzd. Mean ratio ± SEM.Representative electrophysiological traces of AP at a holding potential of −70 mV from GINs in sMDD and sMDD + Trzd groups.Average number of APs evoked during 500 ms stepwise depolarization (CTRL, *n* = 35 neurons from three lines; CTRL + Trzd, *n* = 30 neurons from three lines; sMDD, *n* = 55 neurons from three lines; sMDD + Trzd, *n* = 44 neurons from three lines). One‐way ANONA, ****P < 0.0001. Mean ratio ± SEM.Traces of Na^+^/K^+^ currents were recorded from GINs in sMDD and sMDD + Trzd groups.Average peak values of Na^+^ currents (CTRL, *n* = 46 neurons from three lines; CTRL + Trzd, *n* = 32 neurons; sMDD, *n* = 38 neurons from three lines; sMDD + Trzd, *n* = 36 neurons from three lines). One‐way ANONA, ****P < 0.00001. Mean ratio ± SEM. Source data are available online for this figure.

To verify the efficacy of Trzd, we analyzed the morphology of GINs by performing Sholl analysis (Fig 6A and B). We found that the intersection number, maximum intersections, and sum of intersections were decreased with the treatment of Trzd (Fig 6C–E). Next, we examined whether Trzd could reverse the defects of neuronal activities in sMDD GINs. With the treatment of Trzd for 10 days, we found that the [Ca^2+^]_i_ rise significantly increased in sMDD + Trzd group compared with sMDD group (Fig 6F and G). The results were consistent across cell lines (Fig EV5E). Furthermore, the calcium signaling of sMDD organoids was also reversed in sMDD + Trzd organoid group compared with sMDD organoid group (Figs 6H–J and EV5F). To further verify the neuronal activity of sMDD GINs after Trzd treatment, we then performed electrophysiological experiments. We found that the amplitudes of APs and the evoked AP numbers were decreased with the treatment of Trzd (Figs 6K and L, and EV5G), as well as the half‐width of APs (Fig EV5H). Also, treatment with Trzd led to the fast potassium and inward sodium currents to be comparable with those of the CTRL group (Figs 6M and N, and EV5I).

Taken together, our results demonstrated that 10 μM Trzd treatment could reverse the abnormal calcium signaling and hyperactivity both in sMDD GINs and ventral forebrain organoids.

## Discussion

High heterogeneity of major depressive disorder in symptoms and etiology increases the difficulty of exploring the molecular mechanism during pathogenesis. Studying major depressive disorder with suicide behavior (sMDD), which shares a common symptom, may open a window to revealing the pathogenesis of MDD. So far, it is still a big challenge to model the pathology of sMDD in animal models for the studies of etiology and potential therapeutic drugs. Patient‐derived iPSCs might present a model to study the cellular aspects of sMDD pathogenesis. In this study, we found that GINs derived from patients with sMDD exhibited increased neurite arborization as well as increased neural firing but decreased calcium signaling. Transcriptomic profiling reveals the decreased expression of 5‐HT2C in GINs. Importantly, an FDA‐approved small molecule, Trzd, partially restored the GIN defects.

Previous studies have disclosed that neuronal hyperactivity may cause neuronal loss subsequently (Abiega *et al*, 2016; Fujimori *et al*, 2018), which hinted that increased electrophysiological activity might link to the decreased GABA levels in the end stage of sMDD. Also, dysregulated calcium signaling has been reported to be related to cell death (Ashpole *et al*, 2012). Alteration in the pathophysiology of GINs may play an important role in the pathogenesis of sMDD. Indeed, the reduced neuronal density of GIN subtypes was also found in postmortem tissue in sMDD patients(Otero Losada, 1988; Northoff & Sibille, 2014; Ma *et al*, 2016; Banasr *et al*, 2017). Of note, transcriptomic analysis reveals downregulation of SST in sMDD GINs in our study, suggesting such a possibility. Future work will need to look into the differentiation of GABA subtypes with a better system for long‐term differentiation of GIN subtypes. Despite the lack of obviously changed cell population, we did observe morphological changes, including increased neurite branches in sMDD GINs. A similar phenomenon was recently reported in MDD serotonergic neurons, which showed increased neural morphology (Vadodaria *et al*, 2019b). Thus, our current finding reflected that the hyperactivity and weakened calcium signaling could be the early pathology of sMDD, which might be foled by the GABA neuronal loss during the later pathogenesis, suggesting that GINs could be considered a potential neuronal type involved in the pathogenesis of sMDD. We chose Fluo‐4 AM to examine the calcium signaling of GINs whose differentiation ratio was about 80% in total neurons. Thus, the most neurons we detected were GINs.

The 5‐HT2C receptor is the first, and thus far, the only known G‐protein‐coupled receptor regulated by RNA editing, a post‐transcriptional event that changes the genetic code at the level of RNA (Chagraoui *et al*, 2016). The release of intracellular calcium is temporally delayed by reducing the expression of the 5‐HT2C receptor (Chagraoui *et al*, 2016), and the mRNA expression level of 5‐HT2C was decreased in SSRI‐resistant MDD patients‐derived forebrain neurons (Vadodaria *et al*, 2019a), which aligns with our results. According to a Poisson meta‐analysis, the rates of attempted and committed suicide incidences were usually high in treatment‐resistant depression (Bergfeld *et al*, 2018).

In this study, we found decreased ligand–receptor interactions from our scRNA sequencing data, especially in extracellular matrix (ECM) receptors. Hence, given the multitude of 5HT2C receptors in cell signaling and the regulation of pathophysiological processes, this receptor might be considered a potential drug target. The 5‐HT_2C_R agonist Trzd had restored decreased calcium signaling and abnormal electrophysiological properties in sMDD groups but not in CTRL groups. The possible reason is that the self‐negative feedback regulation of calcium channels in CTRL groups controls the balance of calcium influx (Lee *et al*, 1999).

Patient‐derived iPS cell lines have been used to study the pathogenesis and drug targets in many genetic diseases and degenerative disorders (Parent & Anderson, 2015; Li *et al*, 2019). Although the iPSC model for mood or psychiatric disorders research is still being debated, many researchers have established a study system using the iPSC model. Bipolar patients' iPSC‐derived neurons showed a defect in mitochondrial and calcium signaling, which could be addressed through treatment with lithium (Mertens *et al*, 2015). iPSC‐derived serotonergic neurons from MDD patients exhibited improper neurite length, and forebrain neurons demonstrated SSRI resistance (Vadodaria *et al*, 2019b). These phenomena are consistent with that reported in animal models and human samples, indicating that human iPSCs present a potential model for studying mood disorders (Licinio & Wong, 2016).

Taken together, our human cellular model for sMDD might provide an experimental system for etiology and drug studies. Moreover, drug tests have suggested that 5‐HT2C could be considered as a potential drug target for major depression.

## Materials and Methods

### Generation of induced pluripotent stem cells

Human peripheral blood mononuclear cells (PBMCs) were isolated using a previously described protocol (Seki *et al*, 2012). The cells were reprogrammed using Sendai virus vectors (CytoTune‐iPS 2.0 Sendai Reprogramming kit), following the manufacturer's instructions. After infection for 24 h, the infected cells were transferred to a six‐well plate containing mitomycin C‐inactivated mouse embryonic fibroblasts (MEF feeder cells) at a density of 1.25 × 10^5^ cells per dish and incubated for 24 h with mixed medium (MNC medium/hiPSC medium = 1/1). After infection for 48 h, the medium was replaced with hiPSC medium, and the culture medium was changed every other day. iPS colonies appeared after day 12. Once colonies showed, the culture medium was replaced with Essential 8 (E8) medium and passaged onto a feeder‐free culture system (Life Technology Inc.). All experimental procedures for iPSCs, including blood sampling from donors, were approved by the Nanjing Medical University Medicine Ethics Committee (approval no. (2016)330).

### Differentiation of iPSCs into GINs and ventral forebrain organoids

sMDD iPSCs (SA004, SA005‐1, SA005‐3, SA006, SA007, and SA008) and CTRL iPSCs (NC3‐1, IMR90‐4: WiCell Agreement No. 17‐W0063, RC01001‐A, ihtc‐03, and RC01001‐C) lines were maintained on Vitronectin‐coated (Life Technology Inc) plates with E8 medium (Life Technology Inc). The iPSCs passages followed our previously described methods (Liu *et al*, 2013; Yuan *et al*, 2018; Tang *et al*, 2021). For cells passage, we used Gentle Cell Dissociation Reagent (Stemcell, 1 ml/well) to digest colonies for 1 min when iPSCs colonies became 70% confluent and reseeded pieces in a six‐well plate. The medium was half changed every day.

Neural differentiation was performed according to our previously described methods (Liu *et al*, 2013; Yuan *et al*, 2015, 2018). For neural differentiation, iPSCs colonies were incubated in 1 U/mL dispase (Life Technologies) for 2 min and detached to form the embryonic bodies (EBs). The EBs were suspended in neural induction medium for 7 days. At day 7, the EBs were attached to six‐well plates with 8–10% fetal bovine serum (FBS) in neural induced medium (NIM: 500 ml F12 + 5 ml N2 + 5 ml NEAA) for 8–10 h and changed the medium with pure NIM every other day. The rosette structures could be observed at day 10. At day 16, the rosette‐containing colonies were gently detached by 1 mL pipette, and the fragments were suspended in NIM containing B27 (Life Technologies). To differentiate toward GABA progenitors, 1.0 μM SAG (sonic hedgehog agonist) was used from day 10 to day 25 (Shen *et al*, 2020). At day 28, we dissociated the neurospheres to single cell with TrypLE (Life Technologies) incubated for 6–8 min. Then the single cell suspension was plated at the density of 30,000–50,000 cells/cm^2^ on Matrigel (BD Biosciences) and poly‐l‐ornithine (Sigma) pre‐coated coverslips for further neuronal differentiation.

We generated ventral forebrain organoids by adapting our previously established method (Yuan *et al*, 2020). In total, 1.0 μM SAG was added from day 10 to day 25 in the suspension culture. After day 25, NIM medium was changed every other day. At day 30, these organoids were used for immunostaining or calcium imaging.

### Immunocytochemistry

Cells cultured on coverslips were fixed in phosphate‐buffered saline (PBS) containing 4% paraformaldehyde (PFA) for 30 min at room temperature and washed with PBS for 10 min three times. Then all cells were permeabilized in 0.2% TritonX‐100 for 10 min and blocked in 10% donkey serum for 1 h. All coverslips were incubated at 4°C overnight protected from light in primary antibody, which was diluted in 0.1% TritonX‐100 and 5% donkey serum with PBS. On the second day, the cells were rinsed with PBS and incubated with species‐specific Alexa Fluor 488‐, Alexa Fluor 546‐, or Alexa Fluor 647‐conjugated secondary antibodies (1/2,000; Thermo Fisher), followed by staining with Hoechst 33258 to counterstain the nucleus. Images were obtained using a Nikon 80i or LSM‐800 (Zeiss). The primary and secondary antibodies are listed in Table EV2.

### Quantification of fluorescent images

For analyzing the differentiation rate of GABAergic interneurons, we selected at least nine random fields of each coverslip to count the GABA and TUJ1 positive cells by using Image J. The statistical differences between sMDD and CTRL groups were analyzed by the paired Student's *t*‐test. For analyzing single GIN morphology, we performed Sholl analysis with Image J. The nucleus of GIN was started as the first concentric circle and then drew several concentric circles with a radius difference of 10 μm. We counted the number of intersections between each concentric circle and GIN branches and calculated maximum and sum of intersections. The statistical differences between sMDD and CTRL groups were analyzed by one‐way ANOVA and Nested *t*‐test. All analyses were performed with more than three independent experiments.

### Whole‐cell patch‐clamp recordings

Coverslips were placed in a bath solution containing the following: 145 mM NaCl, 1 mM CaCl_2_, 5 mM KCl, 1 mM MgCl_2_, 5 mM HEPES, and 5 mM glucose (280 mosM, pH 7.3). Neurons were viewed through a 60× water‐immersion objective of an upright microscope (BX51WI, Olympus). Recordings were digitized with a Digidata 1550B (Molecular Devices, Sunnyvale, CA), collected using an Axon MultiClamp 700B differential amplifier (Molecular Devices), and analyzed using Axon Clampex software (Molecular Devices). Recording pipettes with resistances of 6–10 MΩ were filled with an intracellular recording solution containing the following: 130 mM K‐gluconate, 10 mM KCl, 10 mM EGTA, 2 mM MgCl_2_, 0.3 mM Na‐GTP, 2 mM Na‐ATP, and 10 mM HEPES (280 mosM, pH 7.3). Whole‐cell patch‐clamp recordings were performed in GINs at 6–8 weeks.

Briefly, sodium and potassium currents were recorded in voltage‐clamp mode at a holding potential of −70 mV with step‐voltage changes (16 steps from −80 to 60 mV). For eliciting action potentials, neurons were held at −70 mV in a current‐clamp mode, and a series of currents steps (10 steps of 10 pA increments) were then injected. Electrodes (Sutter instrument) were pulled from borosilicate glass using a Sutter instrument puller (model P‐1000), and the electrodes had a resistance of 9–15 MΩ when filled with internal solution. Voltage and current signals were recorded with an Axopatch 700B amplifier (Axon) connected to a Digidata1322A interface (Axon). The data were digitized and stored on disks using pClamp (version 9; Axon). All recordings were performed at room temperature.

### Calcium imaging

We plated GINs and ventral forebrain organoids at day 28 on confocal dishes coated with Matrigel (Corning). Calcium imaging was performed ~2 weeks after plating GINs and 2–3 days after attaching ventral forebrain organoids. We loaded GINs or ventral forebrain organoids with 1 μM Fluo‐4 AM (Life Technologies) loading solution for 15–30 min at 37°C and then followed by 15–30 min at room temperature and washed cells with DPBS (Life Technologies). Finally, cells were placed in live‐cell imaging solution prepared for imaging. Calcium imaging was performed using a confocal fluorescence microscope (Zeiss 800) with 20x objective (single cell scale) at 488 nm excitation.

Time series images were captured at a speed of 1 frame/3.2 s. We stimulated cells with high‐KCL solution (67 mM) about 1 min after starting imaging. Tracing of single cell calcium activities was analyzed using Image J software (http://imagej.nih.gov/ij/) and Prism (v8, GraphPad). The value of peak [Ca^2+^] (*F*_max_–*F*_0_)/*F*_0_ for single cell was obtained by (maximum fluorescence intensity minus initial fluorescence intensity) divided by initial fluorescence intensity. For drug testing experiments, GINs were incubated with drugs diluted in NIM for 5 days, then incubated with Fluo‐4 AM loading solution, washed, and live‐cell calcium imaging performed at day 40 as described above. For drug treatment experiments, GINs were treated with 10 μM Trzd for 5 days, and ventral forebrain organoids were treated for 7 days. The analysis was the same as described above.

### RNA preparation and real‐time PCR

RNA samples were isolated using a Trizol reagent kit (Life Technology Inc), and 1 μg of total RNA from each sample was reverse transcribed into cDNA using the Goldenstar RT6 cDNA Synthesis Kit (Beijing TsingKe). The real‐time PCR was performed in a 20‐μl reaction system, which included 2 μl cDNA, 1 μl forward and reversed primers, 7 μl ddH_2_O, and 10 μl 2X SYBR Green RCR Master Mix (Vazyme). The primer sequences used for qPCR are summarized in Table EV3. We used Glyceraldehyde‐3‐phosphate dehydrogenase (GAPDH) as a housekeeping gene, with 40 cycles of denaturation (98°C for 10 s), annealing (56°C for 10 s), and extension (72°C for 10 s).

### Western blotting

GINs were lysed in RIPA buffer containing cocktail (Roche) for 20 min on ice and then 20–30 μg proteins were obtained and diluted in loading buffer, which contained SDS and β mercaptoethanol. We heated the proteins at 100°C for 8 min to denature the proteins. Then, the proteins were loaded into 10% separation gel at 100 V voltage for about 70–90 min. Next, proteins were transferred to polyvinylidene fluoride membranes at 300 mA for 100 min and blocked in 5% milk dissolved in TBST for 1 h at room temperature. We incubated protein membranes in primary antibodies diluted in 5% milk overnight at 4°C. On the second day, we decanted primary antibodies (listed in Table EV2) and washed the membranes for 5 min three times with TBST. Then the secondary antibodies were incubated on a shaker for 1 h at room temperature. After completing incubation, the membranes were rewashed for 5 min three times with TBST. Finally, the ECL system was used to detect the protein bands. We mixed the luminol substrate solutions A and B at a volume ratio of 1:1 and added onto the surface of membranes in the darkroom. After exposure, the expression of proteins could be obtained.

### Bulk RNA sequencing analysis

Total RNA was extracted using the Trizol reagent kit (Life Technology Inc), and mRNA was enriched by Oligo (dT) beads and then transcripted into cDNA. Library construction and RNA sequencing were completed using the Illumina HiSeq™ 2500 platform. RNA‐seq reads were aligned to the human reference GRCh38 genome and transcriptome using HISAT2 (v.2.1.0) (Kim *et al*, 2019). RNA differential expression analysis was performed using the DESeq2 package (v.1.30.0) (Love *et al*, 2014). The cutoff criterion was set as adjusted *P* value < 0.05, and absolute log2(fold change) ≥ 1. Number of genes upregulated or downregulated were shown by volcano plot using EnhancedVolcano package (v.1.10.0) (Blighe *et al*, 2019). Reactome pathway analysis was conducted on the Reactome online platform (Jassal *et al*, 2020). Gene ontology (GO) analysis and Kyoto Encyclopedia of Genes and Genomes (KEGG) pathway analysis were performed using the clusterProfiler package (v.4.0.2) (Wu *et al*, 2021). Terms with *P*‐value < 0.05 were considered as significant, and expression of genes included in selected terms was exhibited by heat map using the pheatmap package (v.1.0.12) (Kolde, 2015). Interactions of drug and protein targets were analyzed on NetworkAnalyst (Zhou *et al*, 2019), a comprehensive network visual analytics platform, based on DrugBank database (Wishart *et al*, 2006).

### Single‐cell RNA sequencing analysis

For scRNA‐seq experiments, adherent GABAergic neurons were dissociated by 1 ml of trypLE (Life Technology Inc) for 30 min in the condition of 37°C and 5% CO_2_ into single cell when differentiated *in vitro* for 35 days. The cell debris and other aggregates were removed by additional low‐speed centrifugation. Droplet‐based single‐cell RNA sequencing was performed using the 10× Genomics Chromium Single Cell platform, version 3. Approximately 5,000 cells were captured for each sample onto a Chromium Single Cell 3′ Chip (10X Genomics, PN‐120236), and the released RNA in each cell was barcoded through reverse transcription in individual gel beads in emulsions (GEMs). S1000TM Touch Thermal Cycler (Bio Rad) was used for reverse transcription at 53°C for 45 min, followed by 85°C for 5 min and final hold at 4°C. The quality of cDNA was accessed on Agilent 4200 (Agilent Technologies). Libraries of scRNA‐seq were established by the Chromium Single Cell 3′ Library & Gel Bead Kit V3 (10X Genomics, 1000075) and then sequenced on an Illumina NovaSeq6000 with a sequencing depth of at least 750,000 reads per cell.

Raw sequencing data were processed with Cell Ranger software version 3.1 (10X Genomics) for FASTQ generation, and GRCh38 human reference genome was used for alignments. For filtering and unsupervised clustering, the gene expression matrix from Cell Ranger results was used for downstream analysis, using R 4.1.1. Normalization, dimensionality reduction, and cell clustering were performed by Seurat (v.4.0.4) (Hao *et al*, 2021). The filtering criteria for further analysis were setting as: 1000 < = number of UMIs each cell <= 25,000, 1,000 < = number of genes each cell <= 5,000, log10(number of genes each UMI) > 0.8, and mitochondrial transcript proportion < 0.1. Sctransform method was performed for normalization and removing variation of mitochondrial mapping percentage of each Seurat object. The Integration of datasets was also applied to correct batch effects by using integration functions in Seurat. Dimensionality reduction was performed using PCA, and the first 40 principal components were used to generate clusters using the K‐means algorithm and graph‐based algorithm. UMAP plot was shown for visualization of single cell transcriptomic profiles, and similar cells were grouped into clusters with the application of Louvian modularity optimization algorithm. The resolution of FindClusters function was set as 0.6. Known cell marker genes were used to annotate different biological cell types. FindMarkers function in Seurat was applied to find DEGs between different samples with default parameters. PsyGeNET database (Gutierrez‐Sacristan *et al*, 2015) was used to evaluate the associatons of identified DEGs to known mental illnesses. Gene set enrichment analysis (GSEA) based on Ontology gene sets was performed using fgsea (v.1.18.0) (preprint: Korotkevich *et al*, 2021) with nperm parameter set as 1,000. Gene sets with adjusted *P*‐value < 0.05 were considered significant. Related biological and molecular functional terms were plotted and annotated with cell types, which contained the most DEGs overlapped with genes enriched in each term. VoxHunt (v.1.0.0) (Fleck *et al*, 2021) was used for unbiased spatial mapping of single‐cell transcriptome. For assessment of putative cell–cell communication, CellChat (v.1.1.3) (Jin *et al*, 2021) was used to evaluate cell–cell communication by displaying all known pairs of ligands and receptors within and between cell populations across different GABAergic neuron subclusters. The cell‐to‐cell interactions in which number of cells less than 10 were filtered out.

### Injection of virus into organoids

Use a 1 ml pipette tip or a notch pipette to transfer organoids into a medium dish, add 50 μl of NIM medium to each organoid to prevent drying. Place virus on ice immediately after removing from −80°C freezer. Place the organoids under a microscope (Nikon SMZ800N). First, use a 4× microscope to find the location of the organoids, adjust the objective magnification according to the size of the organoids, and then use a micro syringe to start the injection. Each organoid was injected twice, 0.5 μl virus each time. After finishing the injection, add 50 μl of virus dilution solution (preparation of virus dilution solution: 500 μl NIM + 1:50 B27 + 1:2,000 Polybrene + 1:100 PS + 1:100 virus stock solution). After incubation for 24 h, the cells were transferred to T12.5 cell culture flasks, supplemented with 3 ml of NIM medium.

### Ethics statement

Informed consent was obtained from all subjects and that the experiments conformed to the principles set out in the WMA Declaration of Helsinki and the Department of Health and Human Services Belmont Report.

### Statistical analysis

All data were obtained from independent coverslip cultures. Offline data analysis was performed using MiniAnalysis software (Synaptosoft), Clampfit 10.6 (Axon), and Prism 8. SPSS software (version 20.0, SPSS, Inc., Chicago, IL, USA) was used for statistical analysis. All data are presented as the mean ± SEM. Significance was determined using the paired Student's *t*‐test, one‐way ANOVA, two‐way ANOVA, or Nested *t*‐test. A *P*‐value of < 0.05 was considered statistically significant.

**The paper explained**

**Problem**

Major depressive disorder with suicide behavior (sMDD) is a server mood disorder, causing tremendous burden to family and society. Although reduced gamma amino butyric acid (GABA) level has been observed in postmortem tissues of sMDD patients, the molecular mechanism by which GABA levels are altered remains elusive.

**Results**

We generated induced pluripotent stem cells (iPSC) from five sMDD patients and differentiated the iPSCs to GABAergic interneurons (GINs) and ventral forebrain organoids. sMDD GINs exhibited altered neuronal morphology and increased neural firing, as well as weakened calcium signaling propagation, compared with controls. Transcriptomic sequencing revealed that a decreased expression of serotoninergic receptor 2C (5‐HT2C) may be involved in the defected neuronal activity in sMDD. Furthermore, targeting 5‐HT2C receptor, using a small molecule agonist or genetic approach, restored neuronal activity deficits in sMDD GINs.

**Impact**

Our study reported a human cellular model for studying server major depressive disorder, and revealed cellular phenotypes for sMDD in human neurons and ventral forebrain organoids, offering a potential tool for identifying therapeutic approaches for sMDD.

## Author contributions

**Kaiqin Lu:** Data curation; formal analysis; funding acquisition; investigation; writing – original draft; project administration; writing – review and editing. **Yuan Hong:** Data curation; software; formal analysis; investigation. **Mengdan Tao:** Data curation; software; formal analysis; investigation. **Luping Shen:** Data curation; formal analysis; investigation. **Zhilong Zheng:** Investigation; methodology. **Kaiheng Fang:** Investigation; methodology. **Fang Yuan:** Investigation. **Min Xu:** Resources; investigation; methodology; project administration. **Chun Wang:** Resources; methodology; project administration. **Dongya Zhu:** Resources; investigation. **Xing Guo:** Funding acquisition; investigation; writing – original draft; project administration; writing – review and editing. **Yan Liu:** Supervision; funding acquisition; investigation; writing – original draft; project administration; writing – review and editing.

## Disclosure and competing interests statement

The authors declare that they have no conflict of interest.

## Supporting information



Expanded View Figures PDF

Table EV1

Table EV2

Table EV3

Source Data for Expanded View

PDF+

Source Data for Figure 1

Source Data for Figure 2

Source Data for Figure 3

Source Data for Figure 4

Source Data for Figure 5

Source Data for Figure 6

## Data Availability

Raw and processed data of single‐cell RNA‐seq and bulk RNA‐seq used in this study are available in the Gene Expression Omnibus (GEO) under accession GSE208438 (https://www.ncbi.nlm.nih.gov/geo/query/acc.cgi?acc=GSE208438).
