# Supplementary material for: Depressive patient‐derived GABA interneurons reveal abnormal neural activity associated with HTR2C
Source: EMBO Mol Med. 2022 Nov 14;15(1):e16364. doi: 10.15252/emmm.202216364 (PMC9832822; doi:10.15252/emmm.202216364)
Supplement: Supplementary file 1 — Expanded View Figures PDF [file EMMM-15-e16364-s007.pdf]

Expanded View Figures

Figure EV1. Generation of iPSCs and GABA interneurons from sMDD patients. ***Related to*** **Fig 1.**

A Scheme illustrating the generation of iPSCs from CTRL and sMDD patients' peripheral blood mononuclear cell (PBMC). Scale bar = 250 μm. (MEF, Mouse Embryonic Fibroblast).
B Alkaline phosphatase staining and SOX2, NANOG immunostaining in undifferentiated iPSCs from 5 sMDD patients. Scale bar shown in the images.
C Schematic diagram of iPSC differentiation to GINs. The left three pannels: scale bar = 250 μm. The right pannel: scale bar = 50 μm.
D Representative images of GABAergic interneurons with the staining of GABA and MAP2 from CTRL and sMDD groups. Scale bar = 50 μm.
E The proportion of $GABA^+$ cells of $MAP2^+$ neurons from five CTRL cell lines and six sMDD cell lines at day 35. $n \geq 15$. Mean ratio ± SEM.
F Representative images of GABAergic interneurons with the staining of HO and GABA from CTRL and sMDD cell lines. Scale bar = 50 μm.
G The proportion of $GABA^+$ neurons of HO^+ cells from five CTRL cell lines and six sMDD cell lines at day 35. $n \geq 13$.Mean ratio ± SEM.
H Quantification of maximum of intersections shown in five CTRL cell lines and six sMDD cell lines at day 35, $n = 226$ neurons from CTRL groups, $n = 260$ neurons from sMDD groups. Nested t-test, ****$P < 0.0001$ for CTRL versus sMDD. The center line represents the median. Mean ratio ± SEM.
I Quantification of sum of intersections shown in five CTRL cell lines and six sMDD cell lines at day 35, $n = 226$ neurons from CTRL groups, $n = 260$ neurons from sMDD groups. Nested t-test, ***$P = 0.0006$ for CTRL versus sMDD. Mean ratio ± SEM.
J Quantification of maximum of intersections by Sholl analysis shown in five CTRL cell lines and six sMDD cell lines at day 65, $n = 83$ neurons from both CTRL groups and sMDD groups. Nested t-test, *$P = 0.0118$ for CTRL versus sMDD. The center line represents the median. Mean ratio ± SEM.
K Quantification of sum of intersections by Sholl analysis shown in five CTRL cell lines and six sMDD cell lines at day 65, $n = 83$ neurons from both CTRL groups and sMDD groups. Nested t-test, **$P = 0.0027$ for CTRL versus sMDD. Mean ratio ± SEM.

Source data are available online for this figure.

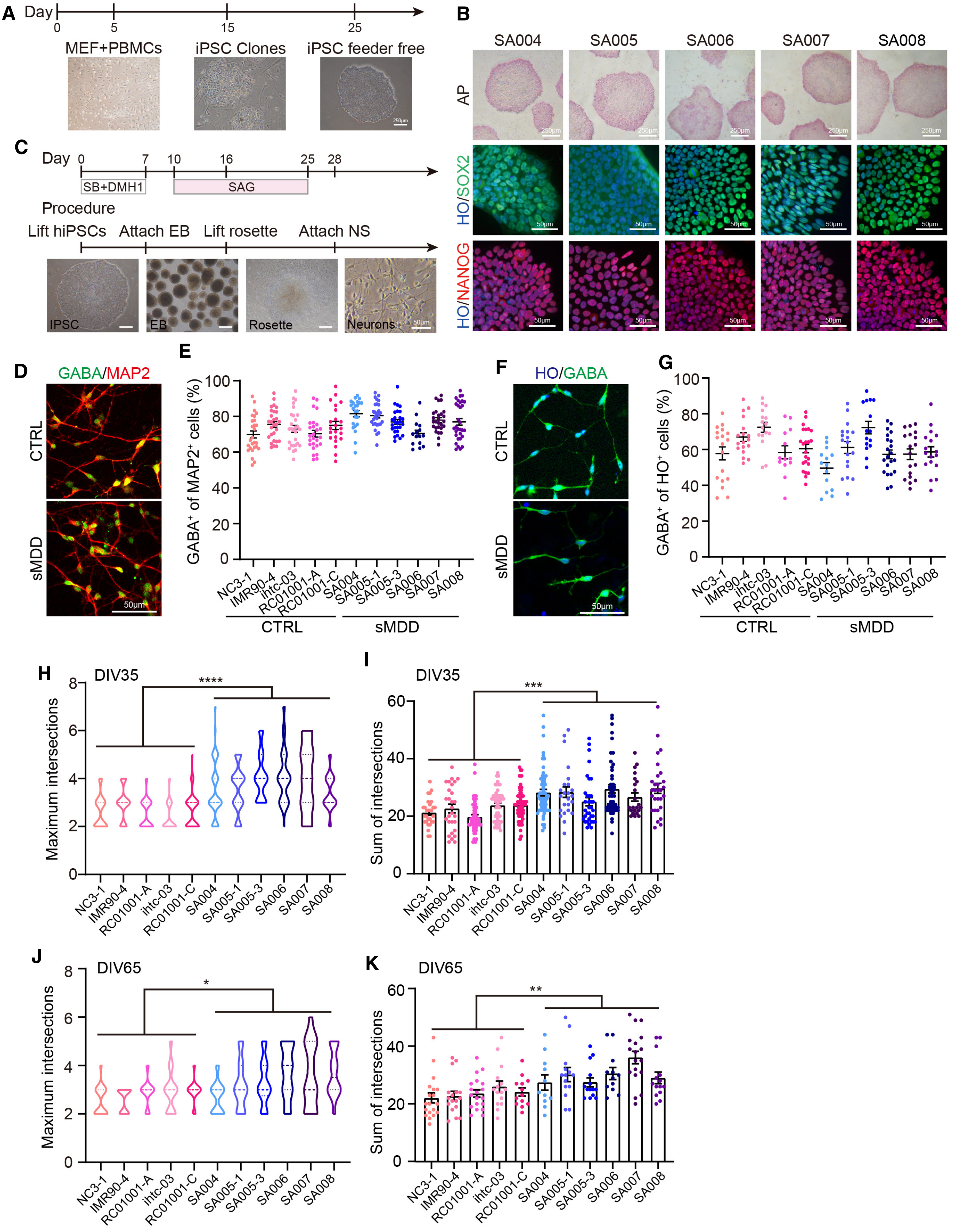

Figure EV1.

©2022 The Authors

Figure EV2. sMDD GINs show neural electrophysiological abnormalities at days 70–80. *Related to* Fig 2.

A Schematic diagram and representative images illustrating the method for whole-cell patch-clamp recording. Representative electrophysiological traces of AP at a holding potential of −70 mV from GINs in sMDD and CTRL groups.

B Amplitude of first AP generated in response to a 10-pA injection (CTRL, $n = 23$ neurons from three lines; sMDD, $n = 25$ neurons from three lines). Nested t-test, $^{**}P < 0.01$. Mean ratio ± SEM.

C Average total number of APs evoked during 500 ms stepwise depolarization (CTRL, $n = 23$ neurons from three lines; sMDD, $n = 25$ neurons from three lines). Nested t-test, $^{*}P < 0.05$. Mean ratio ± SEM.

D Half width of first AP generated in response to a 10-pA injection (CTRL, $n = 23$ neurons from three lines; sMDD, $n = 25$ neurons from three lines). Nested t-test, $P = 0.0872$. Mean ratio ± SEM.

E, F Sample traces of Na^+/K^+ currents recorded from GINs in sMDD and CTRL groups. Average peak values of Na^+ currents (CTRL, $n = 30$ neurons from three lines; sMDD, $n = 24$ neurons from three lines). Nested t-test, $^{**}P < 0.01$. Mean ratio ± SEM.

G Average fast potassium currents of GINs in sMDD and CTRL groups (CTRL, $n = 30$ neurons from three lines; sMDD, $n = 24$ neurons from three lines). Mean ratio ± SEM.

Source data are available online for this figure.

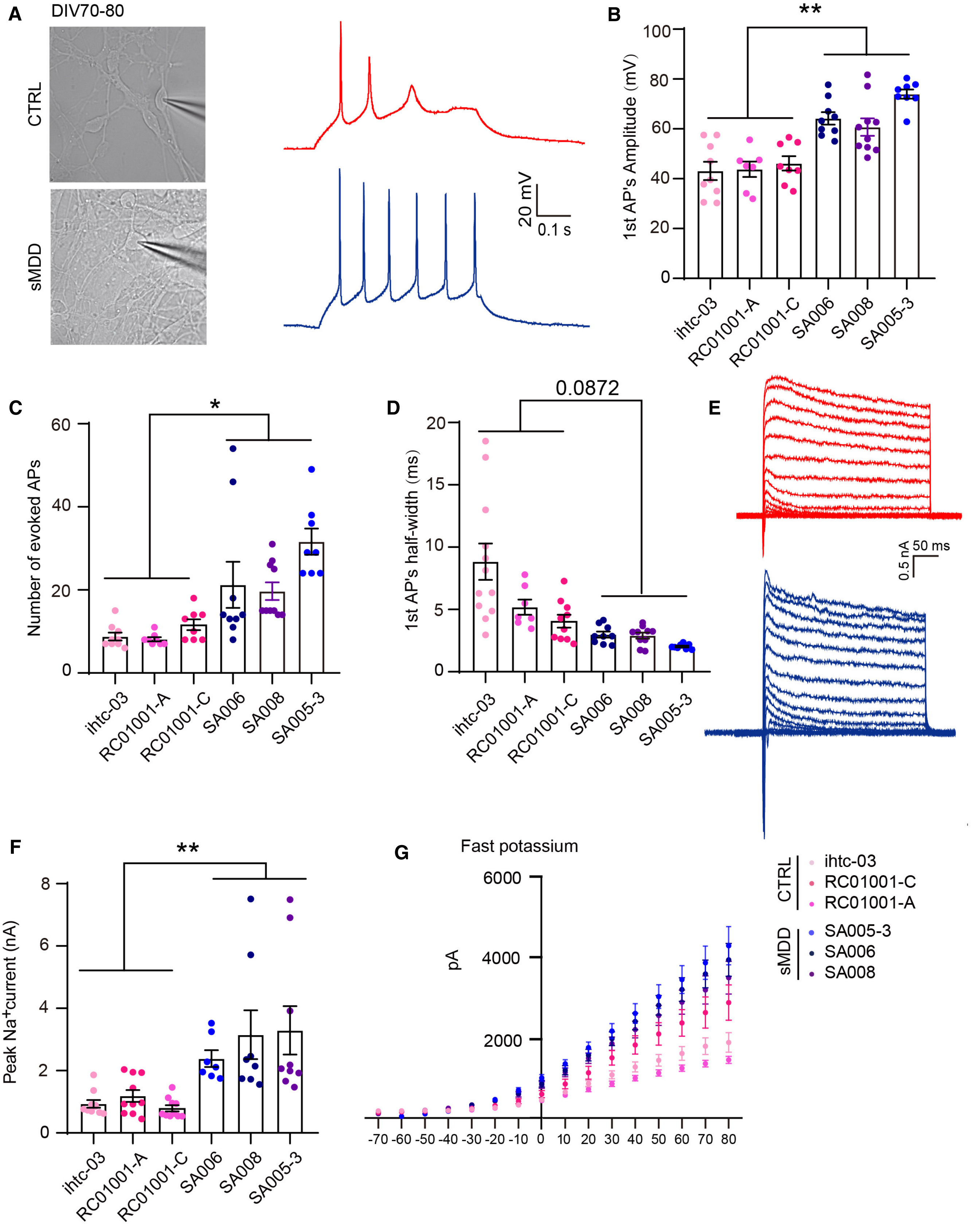

Figure EV2.

 ©2022 The Authors

Figure EV3. Analysis of RNA-seq and DEGs. *Related to* Fig 4.

A Dot plot showing the expression level and percentage of representative marker genes across the six main cell types. The mRNA level of each gene is shown on the linear scale and normalized for different groups.

B VoxHunt spatial mapping of our single cell transcriptome onto data from E13.5 mouse brains, which is available in the Allen Brain database. Sagittal sections are shown and colored by scaled similarity scores.

C Left panel: UMAP visualization of GABAergic neuron subclusters, split by groups. Right panel: Stacked bar chart showing the composition of different GINs for each sample, after 35 days of differentiation.

D Stacked violin plot showing the expression level of ventral transcription factors across different types of GINs.

E Stacked violin plot showing the genes differentially expressed in CTRL and sMDD GINs.

F Bar plots showing the number of inferred interactions and interaction strength for ligand-receptor pairs in CTRL and sMDD GINs, which could be classified into three categories, including Secreted Signaling, ECM-Receptor, and Cell–Cell Contact.

G Cell communication networks showing the number of interactions related to ECM-receptor in CTRL and sMDD GIN subclusters.

©2022 The Authors

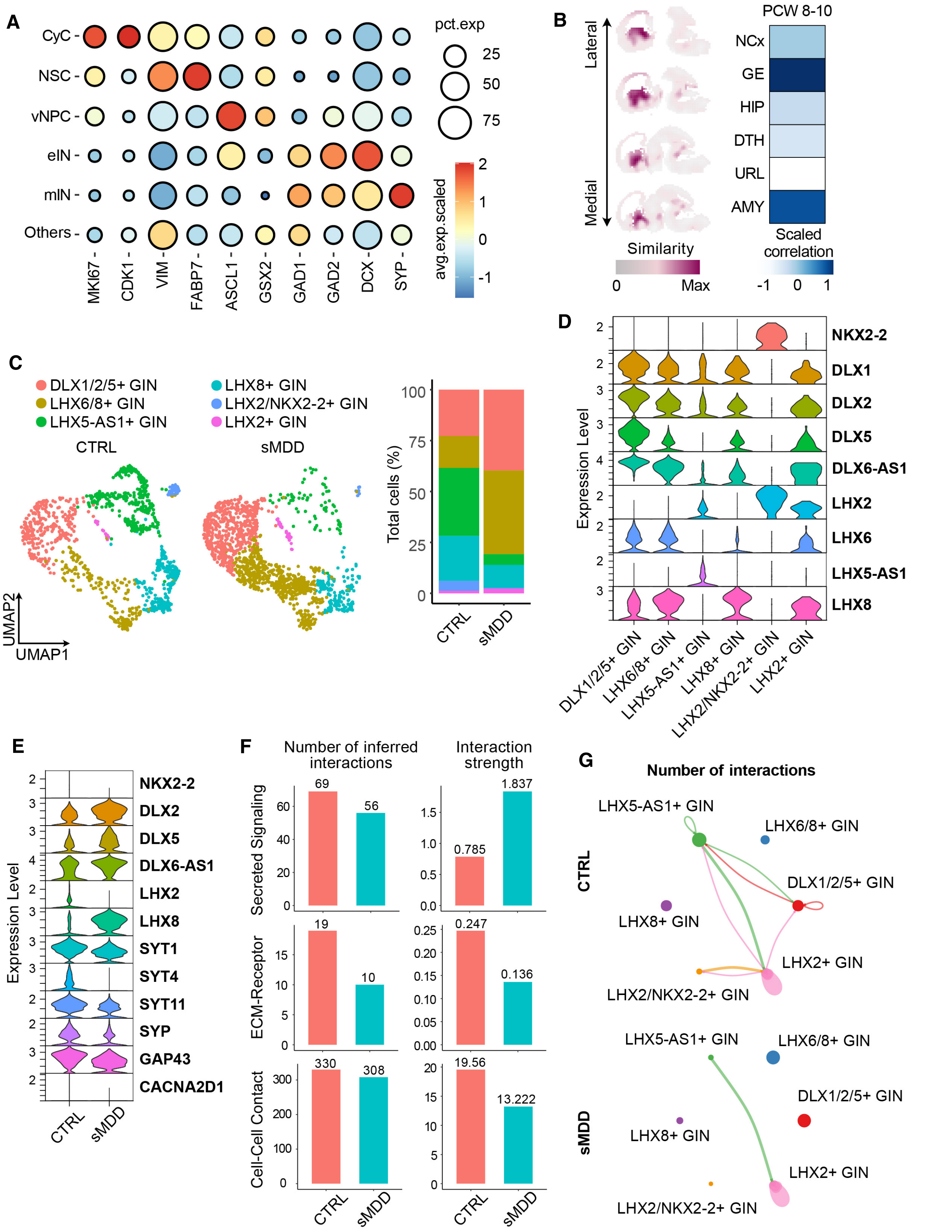

Figure EV3.

 ©2022 The Authors

Figure EV4. Validation of *HTR2C* downregulating in sMDD. *Related to* Fig 4.

A Volcano plot showing all differentially expressed genes between three CTRL and three sMDD samples in bulk RNA-seq.

B Network maps highlighting genes from DEGs with the most targeted molecules according to the Drug Bank database. Yellow dots represent genes, and the blue dots are drugs related to two genes.

C Relative expression levels of 5-HT2CR at days 35–40 from two CTRL cell lines and three sMDD cell lines using qPCR. $n = 3$ from each cell line. Nested t-test, $^{**}P = 0.0077$. Mean ratio ± SEM.

D, E Western blotting analysis and quantification of 5-HT2CR expression in GINs at days 35–40 from three CTRL cell lines and three sMDD cell lines. T-test, $^{***}P = 0.0007$ for CTRL versus sMDD. CTRL, $n = 8$ from three cell lines; sMDD, $n = 11$ from three cell lines. Mean ratio ± SEM.

F Schematic diagram of knockdown lentivirus sequence.

G Representative images of shRNA knockdown virus-infected neurons. Scale bar = 50 μm.

Source data are available online for this figure.

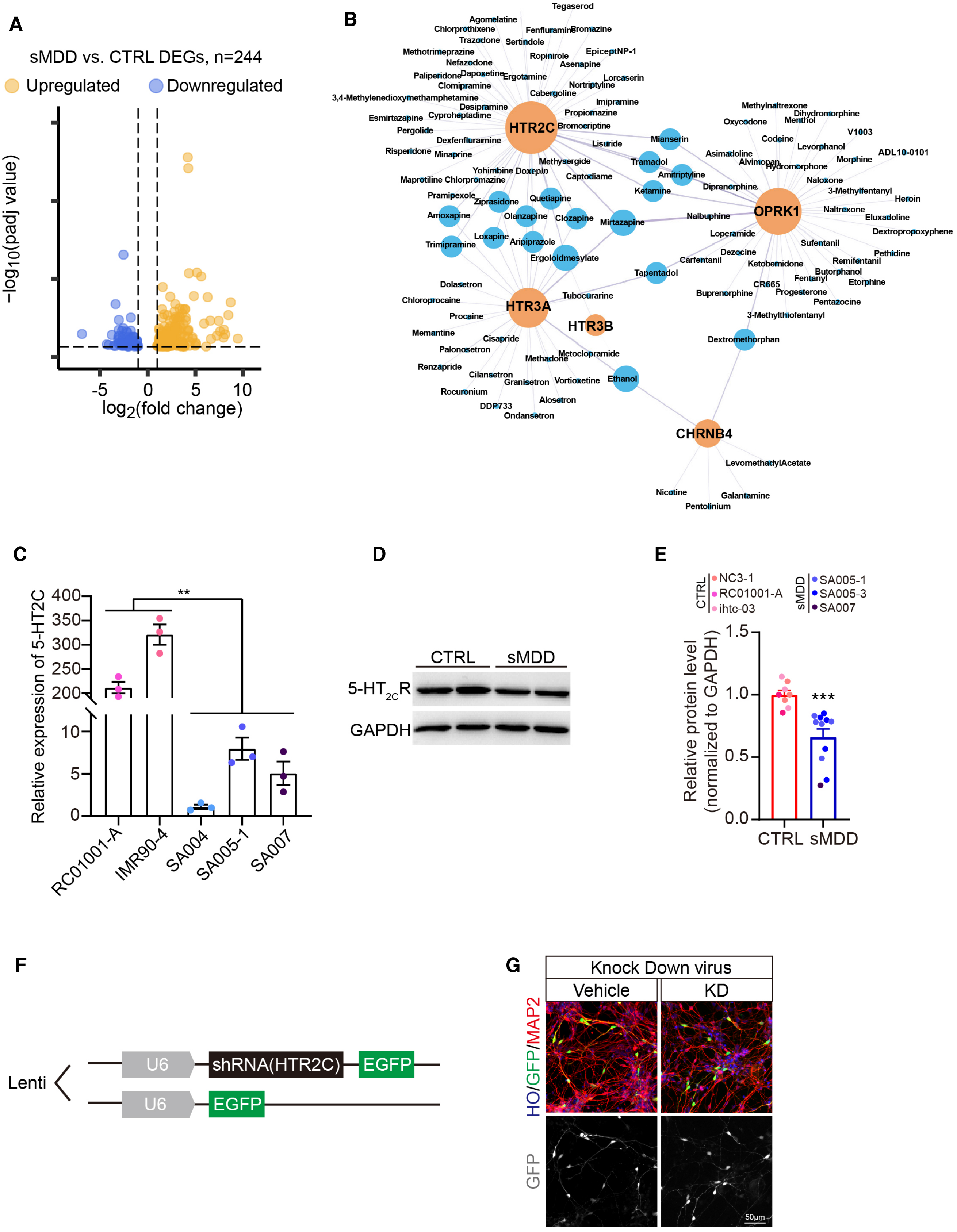

Figure EV4.

 ©2022 The Authors

Figure EV5. Restoration of neural function after overexpression of *HTR2C* and treatment of Trzd. *Related to* Fig 6.

- A Schematic diagram of overexpression lentivirus sequence.
- B Representative images of overexpression virus-infected neurons. Scale bar = 50 μm.
- C Stacked violin plot showing the expression of representative cell type makers across different GIN subclusters.
- D Mean counts of differently expressed genes (DEGs), which are calculated from downsampled data of different GIN subclusters. Mean counts of upregulated genes and downregulated genes are shown in red orange and turquoise respectively.
- E Quantification of peak [Ca^{2+}] (F_{max}–F_0)/F_0 shown per cell line (CTRL, n = 79 neurons; CTRL + Trzd, n = 70 neurons; sMDD, n = 54 neurons; sMDD + Trzd, n = 87 neurons). One-way ANONA, ****$P < 0.0001$ for CTRL versus sMDD, ****$P < 0.0001$ for sMDD versus sMDD + Trzd. Mean ratio ± SEM.
- F The representative trajectory of average intensity changes over time from CTRL, CTRL + Trzd, sMDD, and sMDD + Trzd ventral forebrain organoids in response to 67 mM KCL. CRTL, n = 29 neurons; CTRL + Trzd, n = 31 neurons; sMDD, n = 35 neurons; sMDD + Trzd, n = 43 neurons. Traces are from a representative experiment (the whole quantitative result is shown in Fig 6J). Mean ratio ± SEM.
- G Average half width of first AP generated in response to a 10-pA injection (CTRL, n = 35 neurons from three lines; CTRL + Trzd, n = 30 neurons from three lines; sMDD, n = 55 neurons from three lines; sMDD + Trzd, n = 44 neurons from three lines). One-way ANONA, ****$P < 0.0001$. Mean ratio ± SEM.
- H Amplitude first AP generated in response to a 10-pA injection (CTRL, n = 35 neurons from three lines; CTRL + Trzd, n = 30 neurons from three lines; sMDD, n = 55 neurons from three lines; sMDD + Trzd, n = 44 neurons from three lines). One-way ANONA, ****$P < 0.00001$. Mean ratio ± SEM.
- I Average fast potassium currents of GINs in sMDD and CTRL groups (CTRL, n = 43 neurons from three lines; CTRL + Trzd, n = 34 neurons; sMDD, n = 36 neurons from three lines; sMDD + Trzd, n = 34 neurons from three lines). Two-way ANONA, ***$P < 0.001$; ****$P < 0.0001$, sMDD versus sMDD + Trzd. Mean ratio ± SEM.

Source data are available online for this figure.

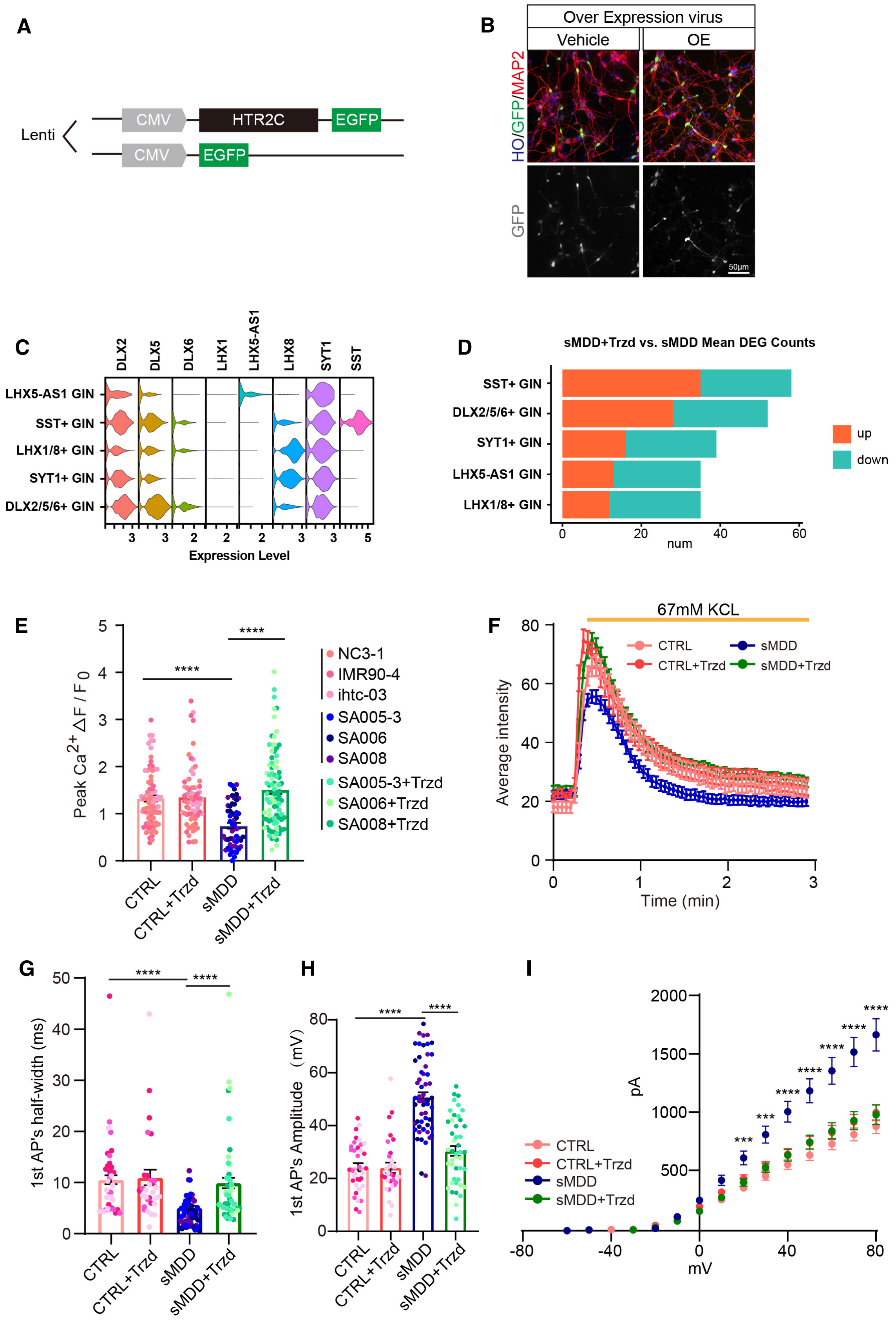

Figure EV5.

©2022 The Authors
