# Supplementary material for: Depressive patient‐derived GABA interneurons reveal abnormal neural activity associated with HTR2C
Source: EMBO Mol Med. 2022 Nov 14;15(1):e16364. doi: 10.15252/emmm.202216364 (PMC9832822; doi:10.15252/emmm.202216364)
Supplement: Supplementary file 2 — Table EV1 [file EMMM-15-e16364-s004.docx]

|  | Cell line | Sex | Age | Diagnosis |
| --- | --- | --- | --- | --- |
| CTRL | NC3-1 | female | 36 | None |
|  | IMR90-4 | female | 16w |  |
|  | RC01001-A | male | 36 |  |
|  | ihtc-03 | female | 28 |  |
|  | RC01001-C | male | 30 |  |
| sMDD | SA004 | male | 53 | Major depressive disorder  that committed suicide at least once |
|  | SA005-1 | female | 43 |  |
|  | SA005-3 | female | 43 |  |
|  | SA006 | male | 34 |  |
|  | SA007 | male | 33 |  |
|  | **SA008** | **male** | **53** |  |

**Table EV1**. iPSCs used in this study.
