# Supplementary material for: Depressive patient‐derived GABA interneurons reveal abnormal neural activity associated with HTR2C
Source: EMBO Mol Med. 2022 Nov 14;15(1):e16364. doi: 10.15252/emmm.202216364 (PMC9832822; doi:10.15252/emmm.202216364)
Supplement: Supplementary file 3 — Table EV2 [file EMMM-15-e16364-s006.docx]

| Antibodies | Isotype | Dilution | Source | Identifer |
| --- | --- | --- | --- | --- |
| GABA | Rabbit IgG | 1:1000 | Sigma-Aldrich | A2052 |
| β-III Tubulin | Mouse IgG | 1:2000 | Sigma-Aldrich | T8660 |
| NANOG | Goat IgG | 1:500 | R&D Systems | AF1997 |
| SOX2 | Goat IgG | 1:1000 | R&D Systems | AF2018 |
| GAD67 | Mouse IgG | 1:1000 | MERCK | MAB5406 |
| NKX2.1 | Rabbit IgG | 1:500 | MERCK | MAB5460 |
| 5-HT2CR | Rabbit IgG | 1:1000 | Abcam | Ab133570 |
| GAPDH | Mouse IgG | 1:5000 | Affinity | T0004 |

**Table EV2**. Antibodies used in this study.
