# Supplementary material for: Depressive patient‐derived GABA interneurons reveal abnormal neural activity associated with HTR2C
Source: EMBO Mol Med. 2022 Nov 14;15(1):e16364. doi: 10.15252/emmm.202216364 (PMC9832822; doi:10.15252/emmm.202216364)
Supplement: Supplementary file 4 — Table EV3 [file EMMM-15-e16364-s001.docx]

| Primer | Forward | Reverse |
| --- | --- | --- |
| *GAPDH* | TCGACAGTCAGCCGCATCTTCTTT | ACCAAATCCGTTGACTCCGACCTT |
| *HTR2C* | CTAATTGGCCTATTGGTTTGGCA | CCACCATCGGAGGTATTGAAAA |

**Table EV3.** Primers used in this study.
