# Supplementary figures and images for: Depressive patient‐derived GABA interneurons reveal abnormal neural activity associated with HTR2C
Source: EMBO Mol Med. 2022 Nov 14;15(1):e16364. doi: 10.15252/emmm.202216364 (PMC9832822; doi:10.15252/emmm.202216364)

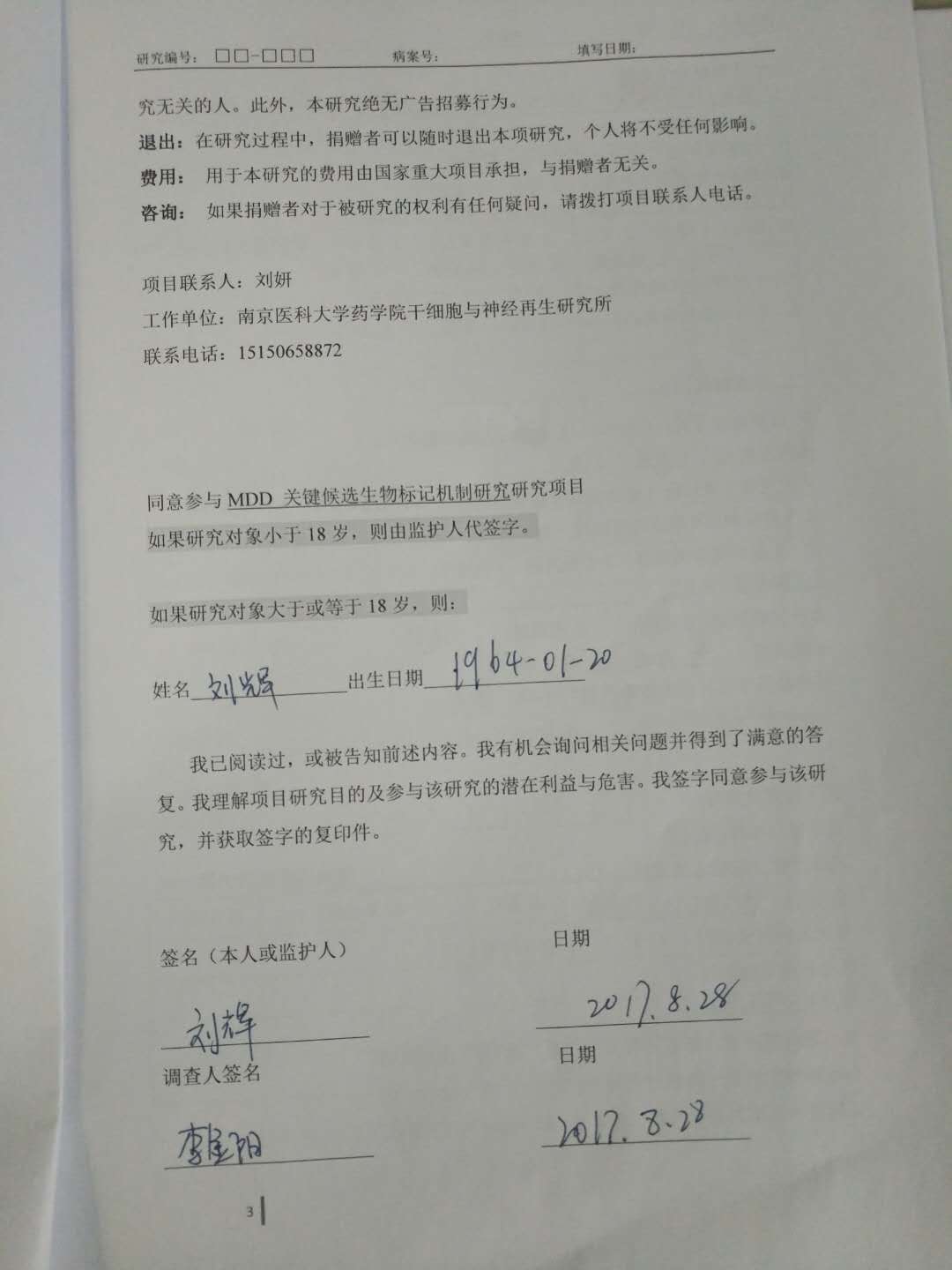

Supplement: Supplementary file 7 — Source Data for Figure 1 [file EMMM-15-e16364-s003.zip › Figure 1/Informed consent/σêÿΦ╛ë.jpg]

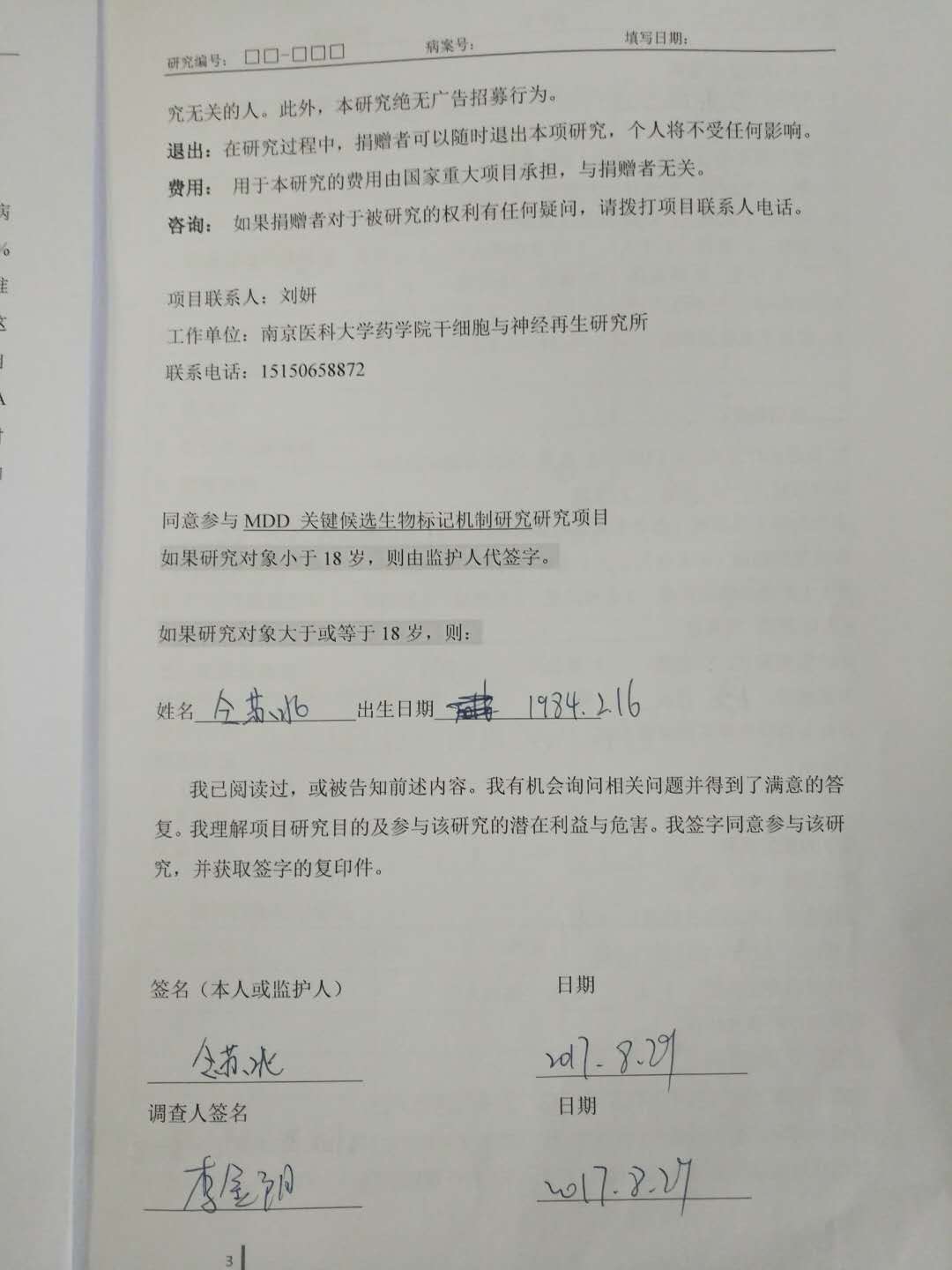

Supplement: Supplementary file 7 — Source Data for Figure 1 [file EMMM-15-e16364-s003.zip › Figure 1/Informed consent/Σ╗¥ΦïÅσîù.jpg]

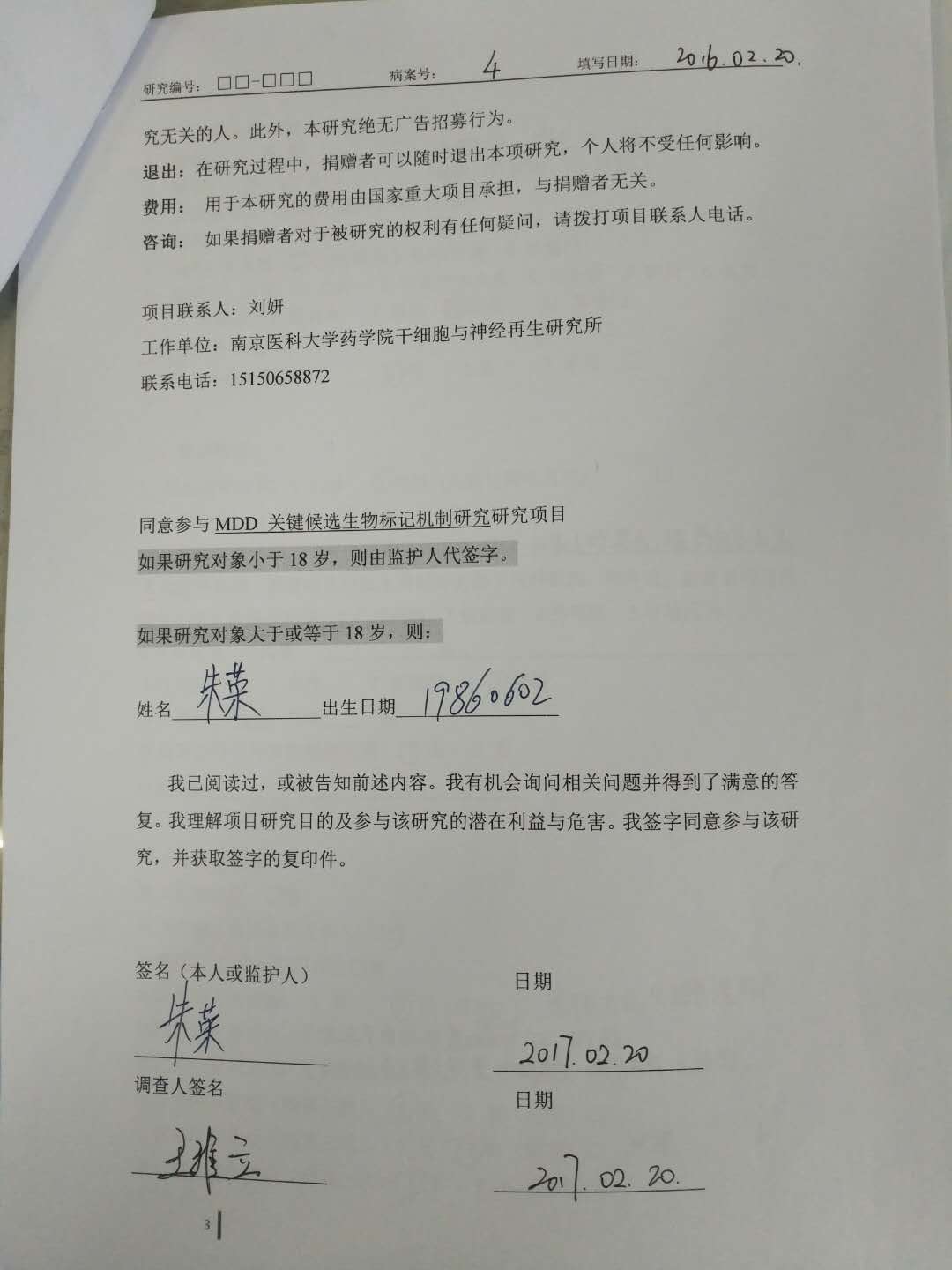

Supplement: Supplementary file 7 — Source Data for Figure 1 [file EMMM-15-e16364-s003.zip › Figure 1/Informed consent/μ£▒Φìú.jpg]

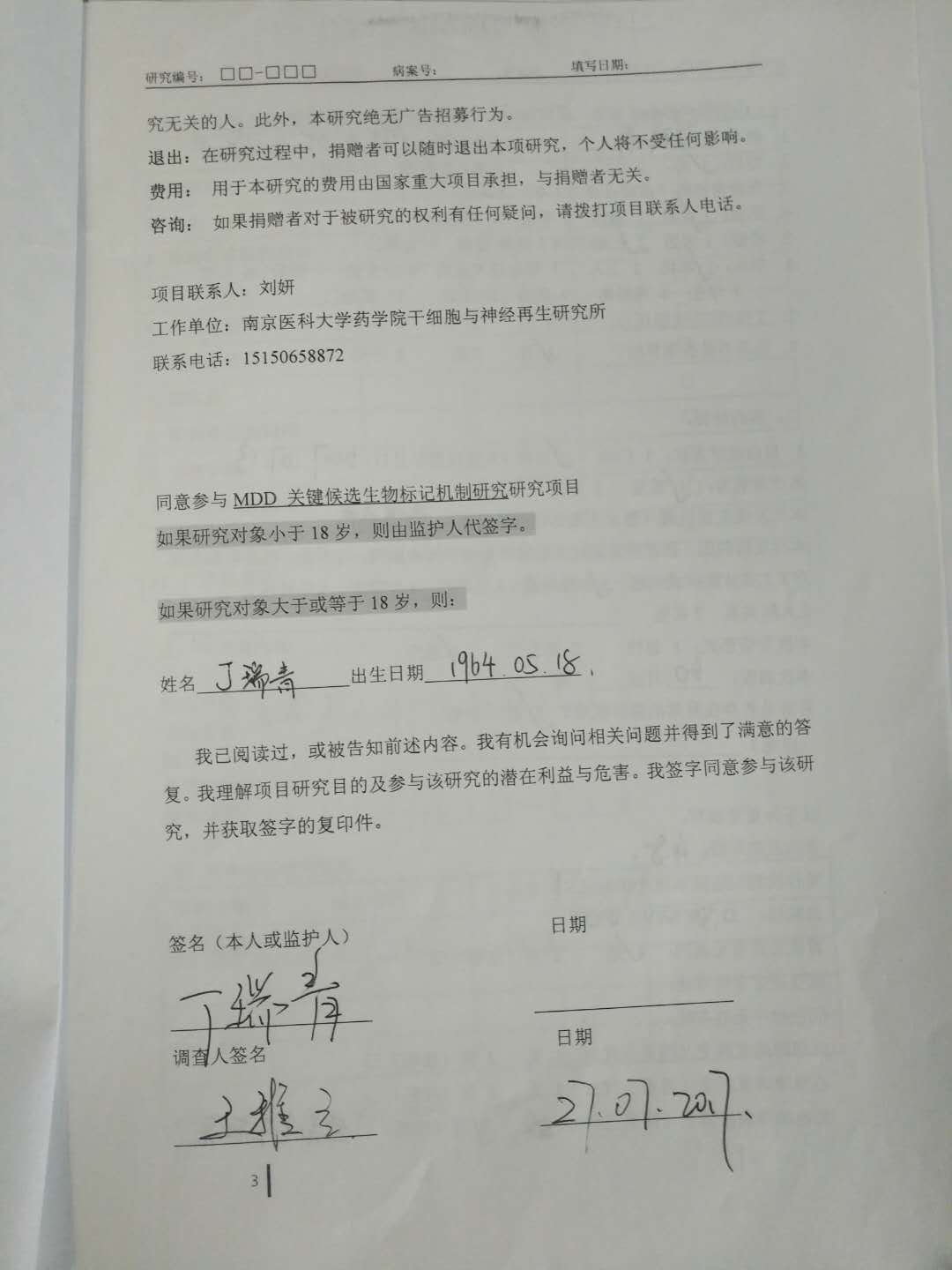

Supplement: Supplementary file 7 — Source Data for Figure 1 [file EMMM-15-e16364-s003.zip › Figure 1/Informed consent/Σ╕üτæ₧Θ¥Æ.jpg]

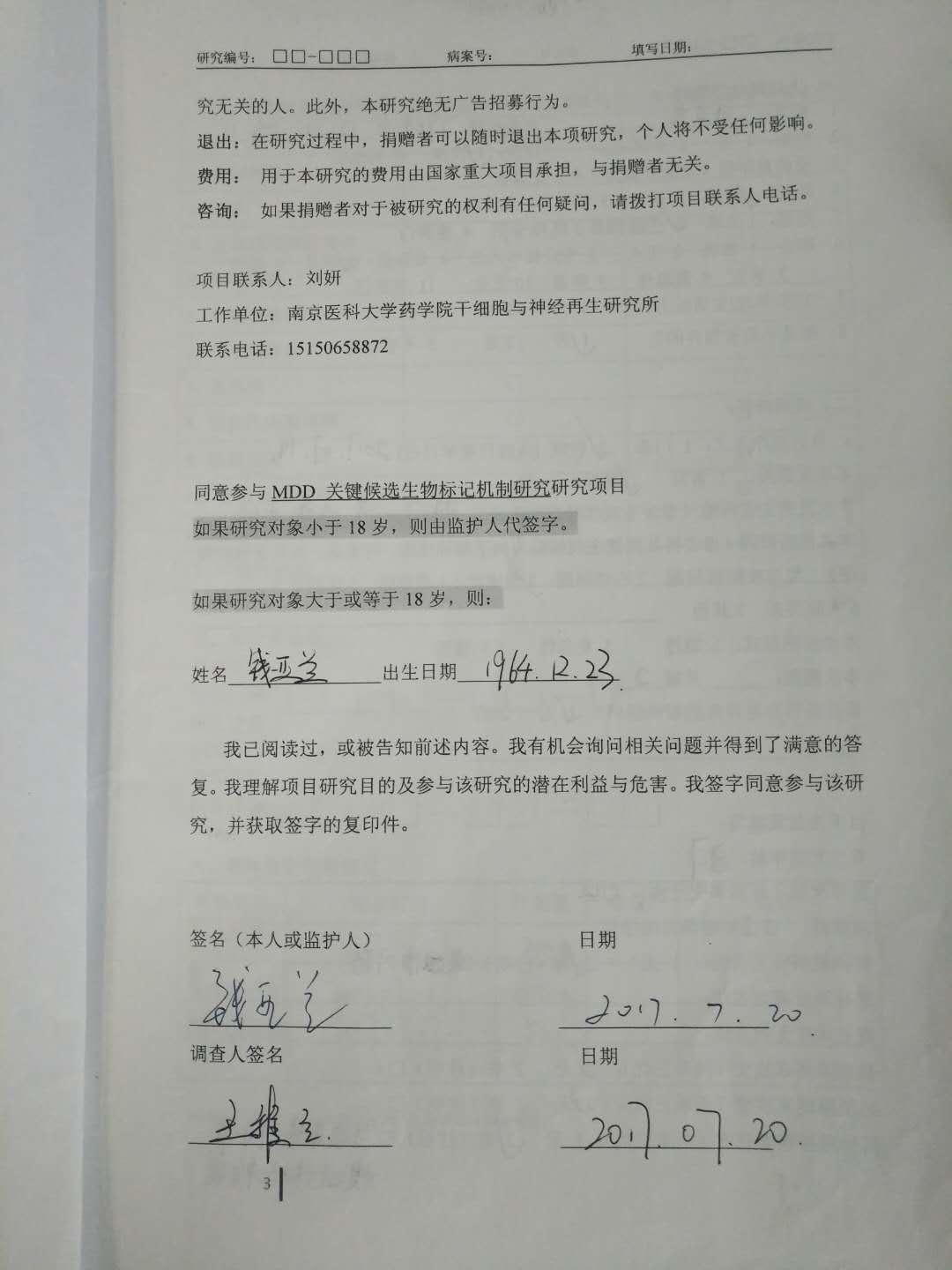

Supplement: Supplementary file 7 — Source Data for Figure 1 [file EMMM-15-e16364-s003.zip › Figure 1/Informed consent/ΘÆ▒Σ║Üσà░.jpg]

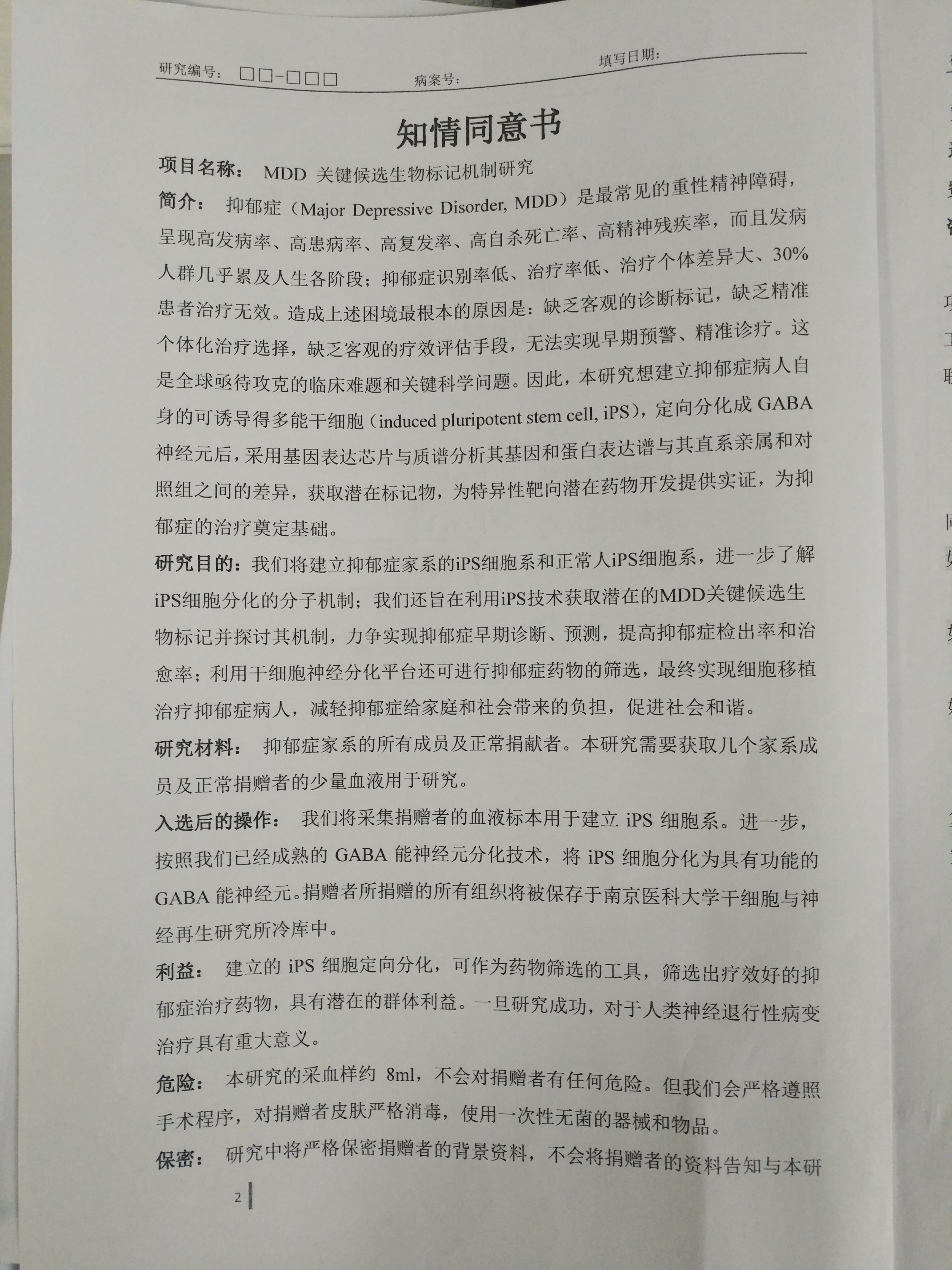

Supplement: Supplementary file 7 — Source Data for Figure 1 [file EMMM-15-e16364-s003.zip › Figure 1/Informed consent/τ1⁄41⁄4Σ╕ÇΘí╡.jpg]

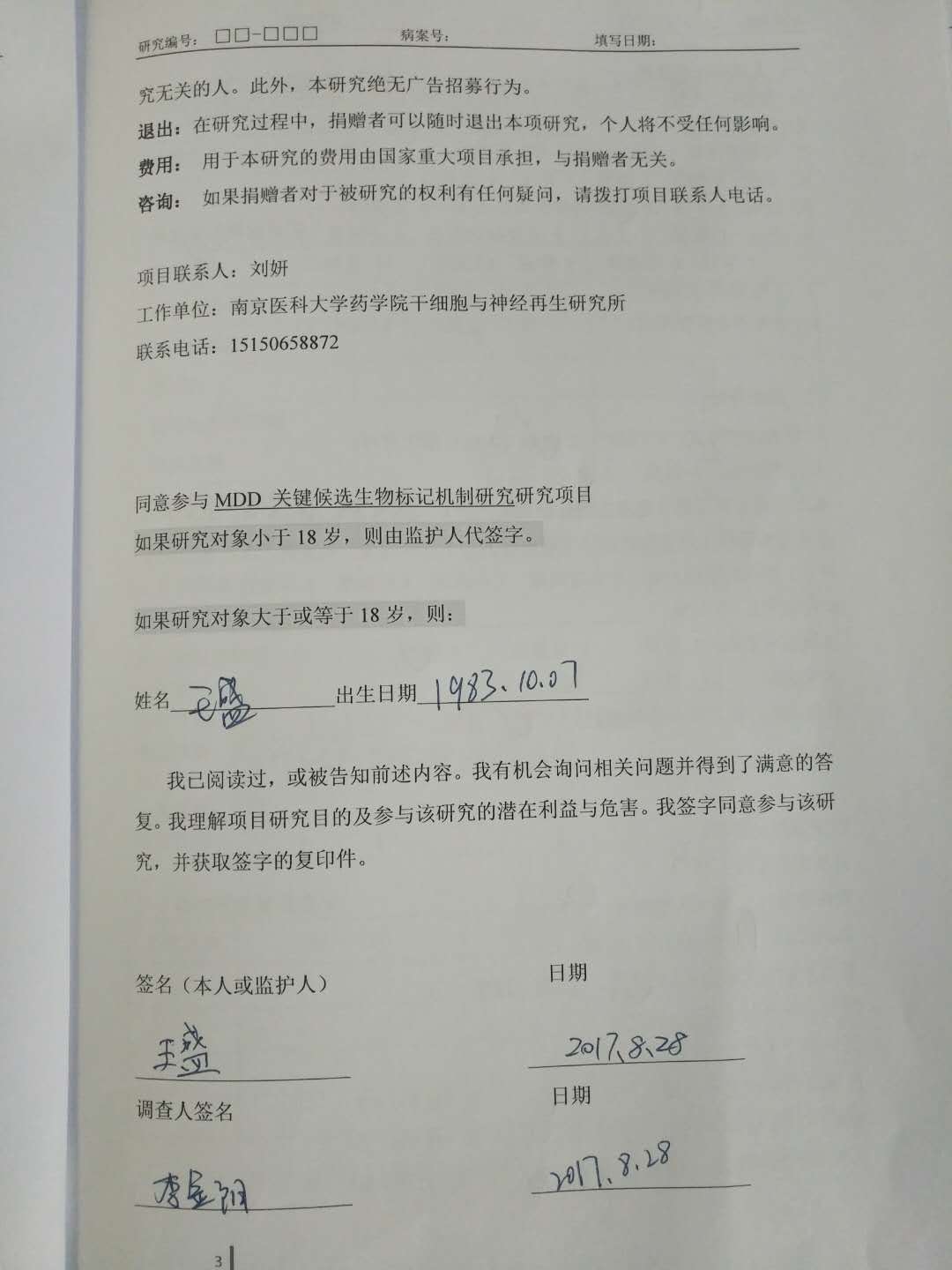

Supplement: Supplementary file 7 — Source Data for Figure 1 [file EMMM-15-e16364-s003.zip › Figure 1/Informed consent/τÄïτ¢¢.jpg]

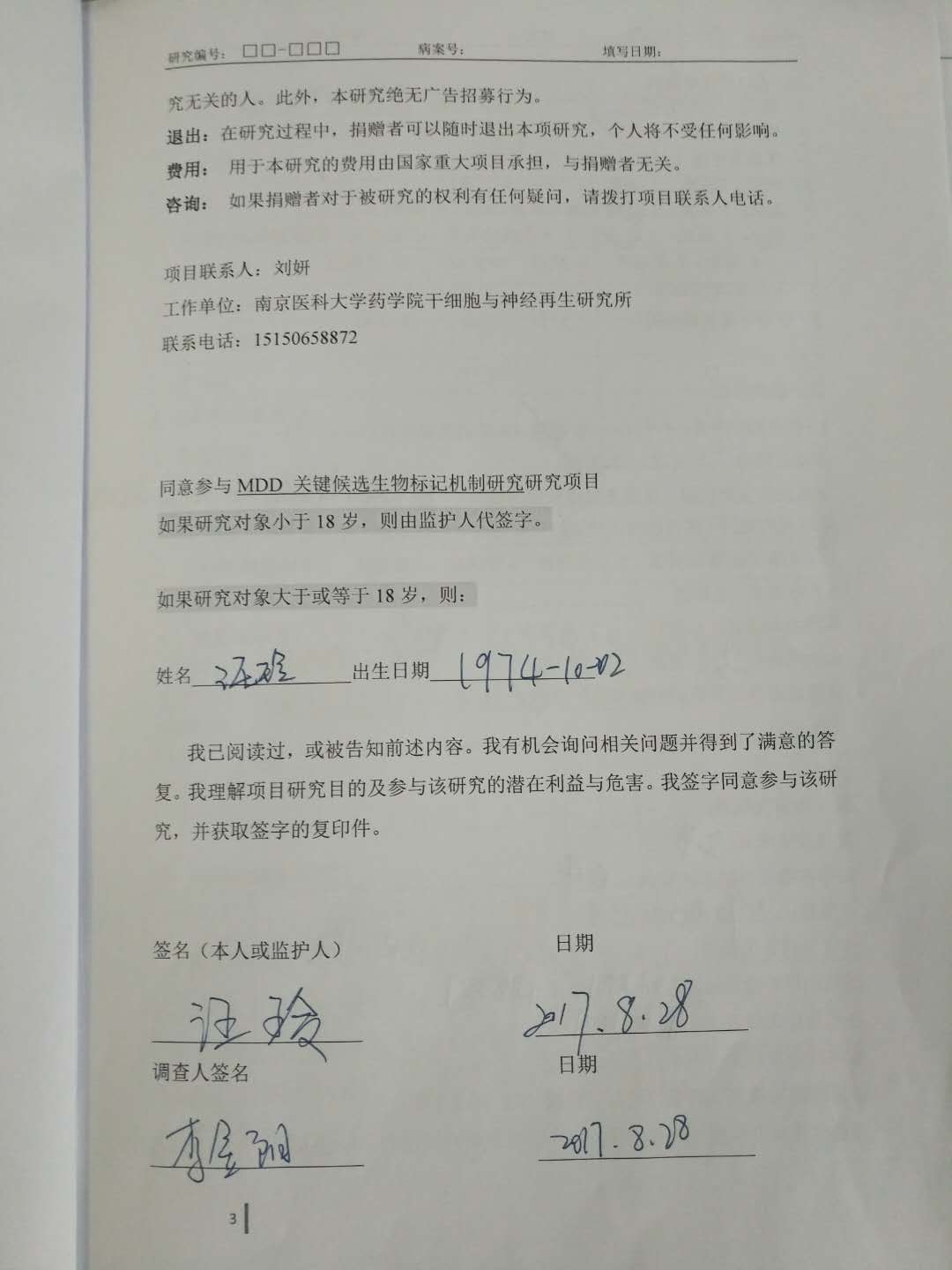

Supplement: Supplementary file 7 — Source Data for Figure 1 [file EMMM-15-e16364-s003.zip › Figure 1/Informed consent/μ▒¬τÄ▓.jpg]

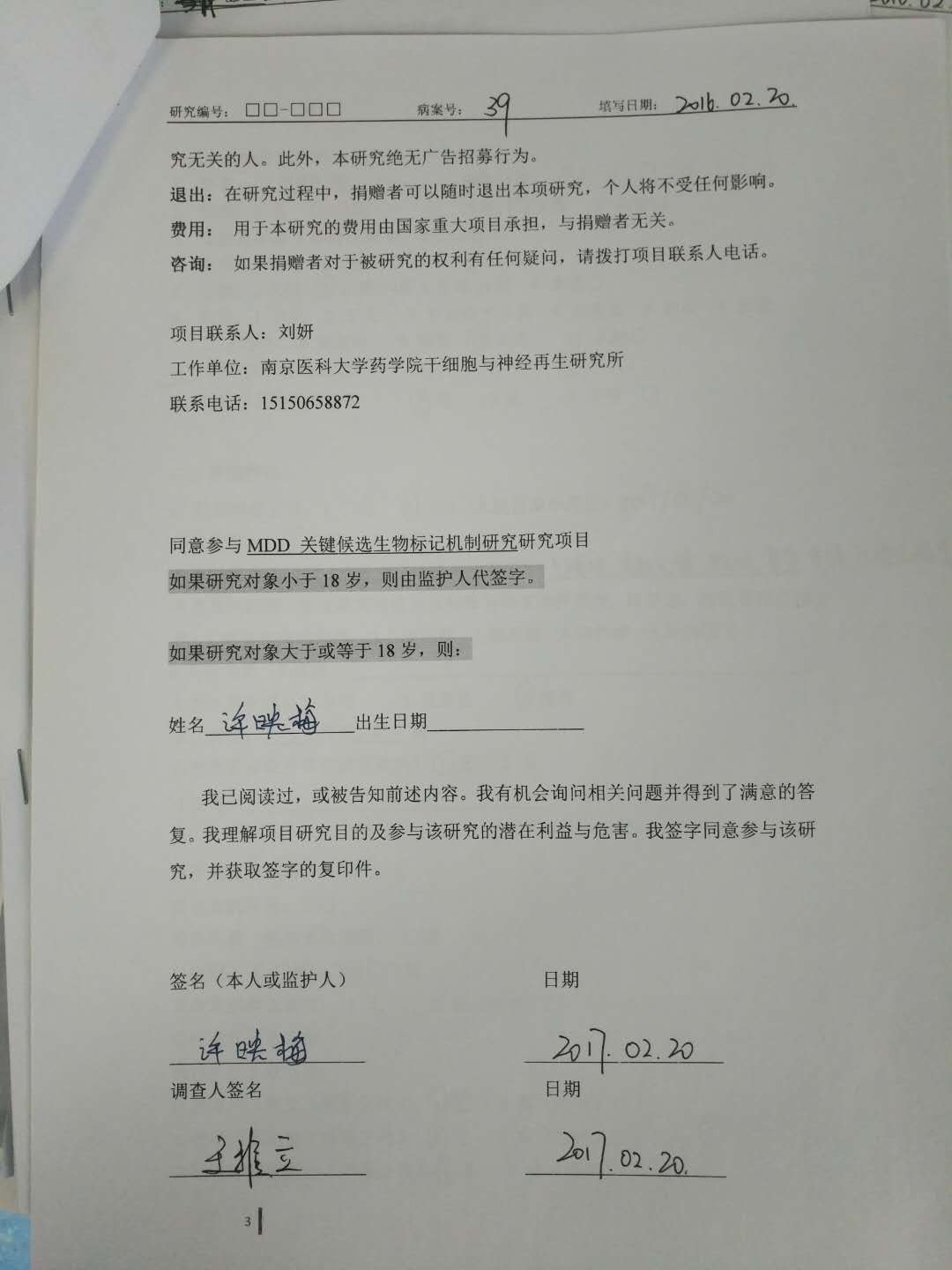

Supplement: Supplementary file 7 — Source Data for Figure 1 [file EMMM-15-e16364-s003.zip › Figure 1/Informed consent/Φ«╕μÿáμóà.jpg]

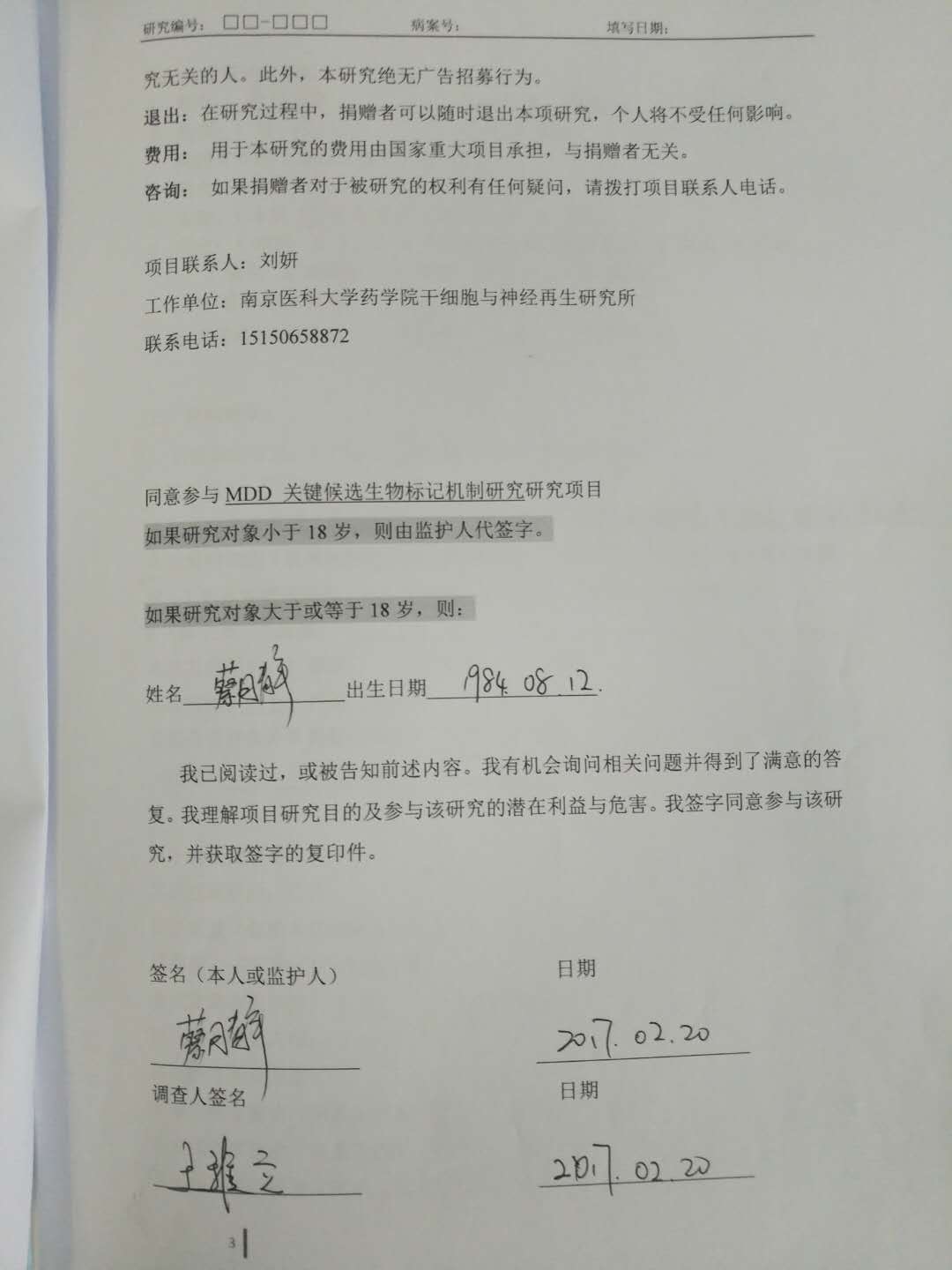

Supplement: Supplementary file 7 — Source Data for Figure 1 [file EMMM-15-e16364-s003.zip › Figure 1/Informed consent/Φöíμ£êΘ¥Ö.jpg]
