# Supplementary material for: Depressive patient‐derived GABA interneurons reveal abnormal neural activity associated with HTR2C
Source: EMBO Mol Med. 2022 Nov 14;15(1):e16364. doi: 10.15252/emmm.202216364 (PMC9832822; doi:10.15252/emmm.202216364)
Supplement: Supplementary file 13 [file EMMM-15-e16364-s009.pdf]

EMBO Molecular Medicine

Depressive patient-derived GABA interneurons reveal abnormal neural activity associated with HTR2C

Kaiqin Lu, Yuan Hong, Mengdan Tao, Luping Shen, Zhilong Zheng, Kaiheng Fang, Fang Yuan, Min Xu, Chun Wang, Dongya Zhu, Xing Guo, and Yan Liu
DOI: 10.15252/emmm.202216364

Corresponding author(s): Yan Liu (yanliu@njmu.edu.cn) Xing Guo (guox@njmu.edu.cn)

Review Timeline:

Submission Date:	24th May 22
Editorial Decision:	20th Jun 22
Revision Received:	12th Oct 22
Editorial Decision:	19th Oct 22
Revision Received:	20th Oct 22
Accepted:	21st Oct 22

Editor: Jingyi Hou

Transaction Report:

(Note: With the exception of the correction of typographical or spelling errors that could be a source of ambiguity, letters and reports are not edited. Depending on transfer agreements, referee reports obtained elsewhere may or may not be included in this compilation. Referee reports are anonymous unless the Referee chooses to sign their reports.)

20th Jun 2022

Dear Prof. Liu,

Thank you again for submitting your work to EMBO Molecular Medicine. We have now heard back from the three referees who agreed to evaluate your manuscript. As you will see from the reports below, the referees acknowledge the potential interest of the study. Still, they raise a series of concerns, which we would ask you to address in a revision of the manuscript.

The recommendations of the referees are rather clear, so there is no need to repeat the points listed below. In particular, Referee #1 pointed out that the electrophysiology experiments need to be performed in more mature iPSC-derived neurons, which must be carefully addressed. During our pre-decision cross-commenting process (in which the referees are given a chance to make additional comments, including on each other's reports), Referee #1 added: "If the authors can satisfactorily address the concerns raised by all three reviewers, the revised manuscript would add significant insights into the molecular and cellular basis of sMDD. As the claims are quite substantial and novel, the evidence provided must be strong enough to justify the claims".

All other issues raised by the referees need to be addressed as well. We would welcome the submission of a revised version within three months for further consideration. Please note that EMBO Molecular Medicine strongly supports a single round of revision. As acceptance or rejection of the manuscript will depend on another round of review, your responses should be as complete as possible.

EMBO Molecular Medicine has a "scooping protection" policy, whereby similar findings that are published by others during review or revision are not a criterion for rejection. Should you decide to submit a revised version, I do ask that you get in touch after three months if you have not completed it to update us on the status.

We are aware that many laboratories cannot function at full efficiency during the current COVID-19/SARS-CoV-2 pandemic and have therefore extended our "scooping protection policy" to cover the period required for a full revision to address the experimental issues. Please let me know should you need additional time and also if you see a paper with related content published elsewhere.

Please read below for important editorial formatting and consult our author's guidelines for proper formatting of your revised article for EMBO Molecular Medicine.

I look forward to receiving your revised manuscript.

Use this link to login to the manuscript system and submit your revision: https://embomolmed.msubmit.net/cgi-bin/main.plex

Kind regards,
Jingyi

Jingyi Hou
Editor
EMBO Molecular Medicine

*****

When submitting your revised manuscript, please carefully review the instructions that follow below. We perform an initial quality control of all revised manuscripts before re-review; failure to include requested items will delay the evaluation of your revision.

We require:

1) A .docx formatted version of the manuscript text (including legends for main figures, EV figures and tables). Please make sure that the changes are highlighted to be clearly visible.

2) Individual production quality figure files as .eps, .tif, .jpg (one file per figure). For guidance, download the 'Figure Guide PDF': (https://www.embopress.org/page/journal/17574684/authorguide#figureformat).

3) A .docx formatted letter INCLUDING the reviewers' reports and your detailed point-by-point responses to their comments. As part of the EMBO Press transparent editorial process, the point-by-point response is part of the Review Process File (RPF),

which will be published alongside your paper.

4) A complete author checklist, which you can download from our author guidelines (https://www.embopress.org/page/journal/17574684/authorguide#submissionofrevisions). Please insert information in the checklist that is also reflected in the manuscript. The completed author checklist will also be part of the RPF.

5) Please note that all corresponding authors are required to supply an ORCID ID for their name upon submission of a revised manuscript.

6) It is mandatory to include a 'Data Availability' section after the Materials and Methods. Before submitting your revision, primary datasets produced in this study need to be deposited in an appropriate public database, and the accession numbers and database listed under 'Data Availability'. Please remember to provide a reviewer password if the datasets are not yet public (see https://www.embopress.org/page/journal/17574684/authorguide#dataavailability).

In case you have no data that requires deposition in a public database, please state so in this section. Note that the Data Availability Section is restricted to new primary data that are part of this study.

7) For data quantification: please specify the name of the statistical test used to generate error bars and P values, the number (n) of independent experiments (specify technical or biological replicates) underlying each data point and the test used to calculate p-values in each figure legend. The figure legends should contain a basic description of n, P and the test applied. Graphs must include a description of the bars and the error bars (s.d., s.e.m.). See also 'Figure Legend' guidelines: https://www.embopress.org/page/journal/17574684/authorguide#figureformat

8) We would also encourage you to include the source data for figure panels that show essential data. Numerical data should be provided as individual .xls or .csv files (including a tab describing the data). For blots or microscopy, uncropped images should be submitted (using a zip archive if multiple images need to be supplied for one panel). Additional information on source data and instruction on how to label the files are available at

.

9) Our journal encourages inclusion of *data citations in the reference list* to directly cite datasets that were re-used and obtained from public databases. Data citations in the article text are distinct from normal bibliographical citations and should directly link to the database records from which the data can be accessed. In the main text, data citations are formatted as follows: "Data ref: Smith et al, 2001" or "Data ref: NCBI Sequence Read Archive PRJNA342805, 2017". In the Reference list, data citations must be labeled with "[DATASET]". A data reference must provide the database name, accession number/identifiers and a resolvable link to the landing page from which the data can be accessed at the end of the reference. Further instructions are available at .

10) We replaced Supplementary Information with Expanded View (EV) Figures and Tables that are collapsible/expandable online. A maximum of 5 EV Figures can be typeset. EV Figures should be cited as 'Figure EV1, Figure EV2" etc... in the text and their respective legends should be included in the main text after the legends of regular figures.

- For the figures that you do NOT wish to display as Expanded View figures, they should be bundled together with their legends in a single PDF file called *Appendix*, which should start with a short Table of Content. Appendix figures should be referred to in the main text as: "Appendix Figure S1, Appendix Figure S2" etc.

- Additional Tables/Datasets should be labeled and referred to as Table EV1, Dataset EV1, etc. Legends have to be provided in a separate tab in case of .xls files. Alternatively, the legend can be supplied as a separate text file (README) and zipped together with the Table/Dataset file.

See detailed instructions here:

.

11) The paper explained: EMBO Molecular Medicine articles are accompanied by a summary of the articles to emphasize the major findings in the paper and their medical implications for the non-specialist reader. Please provide a draft summary of your article highlighting

- the medical issue you are addressing,

- the results obtained and

- their clinical impact.

This may be edited to ensure that readers understand the significance and context of the research. Please refer to any of our published articles for an example.

12) For more information: There is space at the end of each article to list relevant web links for further consultation by our readers. Could you identify some relevant ones and provide such information as well? Some examples are patient associations, relevant databases, OMIM/proteins/genes links, author's websites, etc...

13) Author contributions: the contribution of every author must be detailed in a separate section (before the acknowledgments).

14) A Conflict of Interest statement should be provided in the main text.

15) Every published paper now includes a 'Synopsis' to further enhance discoverability. Synopses are displayed on the journal webpage and are freely accessible to all readers. They include a short stand first (maximum of 300 characters, including space) as well as 2-5 one-sentences bullet points that summarizes the paper. Please write the bullet points to summarize the key NEW findings. They should be designed to be complementary to the abstract - i.e. not repeat the same text. We encourage inclusion of key acronyms and quantitative information (maximum of 30 words / bullet point). Please use the passive voice. Please attach these in a separate file or send them by email, we will incorporate them accordingly.

Please also suggest a striking image or visual abstract to illustrate your article as a PNG file 550 px wide x 300-600 px high.

EMBO Molecular Medicine has a "scooping protection" policy, whereby similar findings that are published by others during review or revision are not a criterion for rejection. Should you decide to submit a revised version, I do ask that you get in touch after three months if you have not completed it, to update us on the status.

***** Reviewer's comments *****

Referee #1 (Comments on Novelty/Model System for Author):

Need to do the experiments in mature iPSC-derived neurons with stable electrophysiology.

Referee #1 (Remarks for Author):

In this study, Lu et al. compared iPSC-derived forebrain GABAergic neurons from suicidal major depressive disorder (sMDD) patients and healthy controls. They found that voltage-dependent Na+ and K+ currents were significantly increased in patient-derived neurons. In addition, action potential amplitude was increased and half-width was decreased. Furthermore, KCl-evoked increase in intracellular Ca2+ was significantly decreased in patient neurons. Through bioinformatics analyses, the authors found that the expression of HTR2C was significantly decreased in patient neurons. Most intriguingly, the electrophysiological and Ca2+ imaging phenotypes were rescued by the overexpression of HTR2C in patient neurons. HTR2C knockdown in control neurons mimicked the phenotypes in patient neurons. Even more strikingly, application of 5HT2C receptor agonist Trazodone mimics the rescue effects of HTR2C overexpression.

Overall issues:
(A) While the dataset appears to be consistent, there are lots of key questions that raise the possibility of alternative explanations of the data. Most importantly, the authors need to provide staining of MAP2 and NeuN to show that the neurons are differentiated to the same density and maturity. It is pretty hard to do electrophysiology on 6-week (42-day) old neurons. No clear phase contrast image is provided to show how the neuronal cultures look like. The example in Fig. 2a showed that the density of neurons appeared to be quite low. There may be differential maturation of patient neurons vs. control neurons, which may be related to the morphological differences in Fig. 1.
(B) The manuscript is not clearly written. The title and the abstract do not articulate the potentially exciting discovery of the phenotypes in sMDD neurons and the rescue by HTR2C overexpression or Trazodone. There are many important typos and grammatically issues that need to be fixed in order for the manuscript to become a permanent record.

Specific issues:

(1) Fig. 1c, the ratio of GABA+/Tuj1+ cells might not reflect the situation accurately. Need to show GABA+/MAP2+ and GABA+/DAPI ratios. Otherwise, the data may not match the scRNAseq data of Fig. 4b, which does not appear to show that high percentage of mIN or eIN among neurons.
(2) How was Sholl analysis done if you did not sparsely label single neurons in Fig. 1? It is problematic to do morphological measurements when the neurons are still growing their processes at day 35. Please do Sholl analysis and also measure neurite length, branch number etc. in mature neurons at day 60 or later.

(3) Frankly, Fig. 1 reports a phenotype very distinct from the rest of the study. It does not need to be in this manuscript, unless the authors can connect the morphological phenotype to the other phenotypes. For example, does Trazodone make the neurites of sDD neurons less elaborate?
(4) Fig. 2, can the frequency of evoke action potentials be analyzed? Do the neurons show spontaneous action potentials?
(5) Fig. 2b,d: evoked action potentials in control neurons appeared to be very abnormal. In addition, Na+ currents were very small in control neurons. It seems that the neurons are simply not mature enough to produce action potentials in a reliable manner. Please do electrophysiology in mature neurons at day 70 or later.
(6) Related to (3), evoked action potentials appeared to be very unstable, particularly in control neurons. Thus, the authors compared the properties of the first AP. This is highly problematic. A train of AP should have the same amplitude. This basic property of neurons is simply not seen in the recordings, casting doubts on how good the neuronal differentiation was.
(7) Fig. 4c is confusing and not described in detail. Is "total" the sum of all other bars?
(8) Fig. 4d is unclear on what are being compared in the GO analysis, control vs. sMDD scRNAseq data in three different types of cells (GABAergic neurons, GABAergic progenitors and NSC)? It would be clearer if the data are segregated and focused on GABAergic neurons. The study does not address anything on GABAergic progenitors or NSCs. There is no need to show them. So is the format for Fig. 5h.
(9) Fig. 5j needs clarification. What does it mean that some CAMK genes are above the line and some are below the line?
(10) Resolution of SupFig. 1 is too low, particularly for Fig. S1c. It is hard to see the neurons. This is very important and allows the reviewers to judge how good the differentiation was.
(11) Does the bulk RNAseq data show a significant difference in the expression levels of HTR2C? This needs to be documented along Fig. S3c.
(12) Fig. 6: how did trazodone selectively change the electrophysiological properties and intracellular Ca2+ signals only in patient neurons, but not in control neurons? Does trazodone induce the expression of HTR2C only in patient, but not in control neurons? The trace in Fig. 6g and j for sMDD+Trzd appeared as if the neurons become immature. The sharp outward A-type K+ current in Fig. 6j was not seen in Fig. 2g.
(13) The study reports electrophysiological results from a very large number of neurons. It is highly challenging to record that many neurons. It would demonstrate the rigor of the study by putting the summary Excel file of the electrophysiological data in the supplemental information.
(14) All RNAseq data (sc and bulk) must be deposited to GEO so others can examine the data after publication. Please provide GEO access numbers of the deposited data.
(15) Is GIN for GABAergic interneuron? Need to define it.
(16) Page 3, line 93, there are six lines of iPSCs, but the text mentioned five. Is SA005-1 and SA005-3 from the same subject? Need to clarify.
(17) Should "committed suicide at least once" be "attempted suicide at least once". Death can only occur once.
(18) There are many other typos and grammatical issues that require careful proofreading.
(19) The discussion is too long and some parts of it are speculative or superfluous. Please consider spending more words on describing the results than on speculative discussions.

Referee #2 (Remarks for Author):

The manuscript "Depressive patient-derived GABA interneurons reveal abnormal neural activity associated with HTR2C," by Lu et al reports that GABAergic interneurons (GINs) derived from iPSCs of major depressive disorder patients with suicide behavior (sMDD) exhibit abnormal neuronal morphology and activity. Transcriptomic analyses at both single cell- and bulk- levels further reveal a decreased expression of serotoninergic receptor 2C (5-HT2C). This study goes on to show that restoring the expression of 5-HT2C using its agonist or genetic approach could rescue the deficits of neuronal activity in sMDD GINs. Overall, this is an excellent and well-written manuscript describing novel and important findings suitable for publication in EMBO Molecular Medicine, with minor revisions.

Major comments:

1. The authors observed an increased neurite branches and neural complexity in the sMDD GINs. However, based on the culture density shown in Fig. 1b, it is not clear how the morphology of individual neurons was analyzed. Furthermore, would these morphological phenotypes be developmental stage-dependent? A time-course study may provide more information.

2. The observation of hyperexcitability in sMDD GINs is very interesting. A more detailed characterization of the action potential kinetics, such as action potential threshold, decay time, and the fast afterhyperpolarization (fAHP), would further strengthen their conclusion. Moreover, are there changes in synaptic transmission, such as spontaneous and miniature inhibitory postsynaptic currents, in sMDD GINs?

3. The single cell RNAseq on sMDD GINs provides novel and meaningful mechanistic insights. More analyses on the RNAseq data would be important for understanding the cellular phenotypes they observed in sMDD GINs. For example, are there changes in cell composition? Is the developmental trajectory altered in sMDD GINs?

4. The nearly complete rescue of calcium signaling and electrophysiological properties in sMDD GINs by trazodone hydrochloride treatment are notable. However, the lack of effect in controls are very surprising and warrant consideration in the discussion.

Referee #3 (Remarks for Author):

In this study, Lu and colleagues generated induced pluripotent stem cells (iPSCs) from major depressive disorder patients with suicidal behavior (sMDD), and differentiate the patient-derived iPSCs to GABAergic interneurons and ventral organoids. Gene profiling analysis showed neural activity related pathways were altered in sMDD groups, as well as the decreased expression levels of serotoninergic receptor 2C subtype (5-HT2C), which could be restored by overexpression of HT2RC. Moreover, the authors also used the sMDD patient-derived GABAergic interneurons to test small molecules. Overall, these findings are intriguing and provide valuable model for studying the mechanism of sMDD, as well as drug discoveries. There are a few aspects need further addressed for additional merits.

1. HT2RC was identified from bulk-seq data. Are other serotoninergic receptor subtypes altered in gene profiling data? Such as 5HT1A?
2. The authors tested calcium signaling by using Fluo-4 AM, which would examine the signal from non-GABA neurons. It would be helpful to make a claim in discussion.
3. There are many subtypes in GABAergic interneurons, for example, calretinin+ interneuron is an important subtype involved in calcium signaling. Is the ratio of calretinin subtype affected in sMDD groups?
4. In figure 4e, the expression level of SST is decreased in sMDD groups. Somatostatin is a major GABA interneuron subtype in the brain. It might be helpful to identify whether somatostatin subtypes changed in sMDD groups.
5. The authors claim the percentage of GABAergic interneurons is around 90%. What are the rest type of cells? Are they glial cells?
6. In introduction, the authors could include more literatures to introduce psychiatry studies by using patient-derived iPSCs, especial brain organoids.
7. In results, the authors claim they injected virus to knock down or overexpression of HT2RC in organoids, detail methodology should be described in methods.
8. One reference (Mol Psychiatry 2019 Jun;24(6):795-807) in the manuscript showed decreased HT2RC levels in selective serotonin reuptake inhibitors (SSRI) resistant patient iPSC-derived neurons, but not in SSRI-responsive groups. It might be helpful to discuss the correlation between SSRI-resistant and suicidal behavior.

Reviewers' comments:

We sincerely thank the editor and all reviewers for their valuable feedback to improve the quality of our manuscripts.

Referee #1 (Remarks for Author):

In this study, Lu et al. compared iPSC-derived forebrain GABAergic neurons from suicidal major depressive disorder (sMDD) patients and healthy controls. They found that voltage-dependent Na+ and K+ currents were significantly increased in patient-derived neurons. In addition, action potential amplitude was increased and half-width was decreased. Furthermore, KCl-evoked increase in intracellular Ca2+ was significantly decreased in patient neurons. Through bioinformatics analyses, the authors found that the expression of HTR2C was significantly decreased in patient neurons. Most intriguingly, the electrophysiological and Ca2+ imaging phenotypes were rescued by the overexpression of HTR2C in patient neurons. HTR2C knockdown in control neurons mimicked the phenotypes in patient neurons. Even more strikingly, application of 5HT2C receptor agonist Trazodone mimics the rescue effects of HTR2C overexpression.

We appreciate the reviewer's positive comments on our current works.

There are a few aspects needs further consideration for additional merits.

Overall issues:
(A) While the dataset appears to be consistent, there are lots of key questions that raise the possibility of alternative explanations of the data. Most importantly, the authors need to provide staining of MAP2 and NeuN to show that the neurons are differentiated to the same density and maturity. It is pretty hard to do electrophysiology on 6-week (42-day) old neurons. No clear phase contrast image is provided to show how the neuronal cultures look like. The example in Fig. 2a showed that the density of neurons appeared to be quite low. There may be differential maturation of patient neurons vs. control neurons, which may be related to the morphological differences in Fig. 1.

We appreciate the reviewer's questions and valuable suggestions sincerely. We addressed all these questions and improved our results in this revision. In the following part of this revision, we have performed IHC and electrophysiology again to solve these questions individually. We hope the revised manuscript can better describe our findings.

In this revision, we have analyzed the proportion of GABAergic interneurons (GINs) over MAP2+ neurons and HO+ cells, respectively. We performed Sholl analysis in mature GINs at day 65 and compared GINs morphology such as the longest neurite length, primary branch number between different groups. Furthermore,

we performed electrophysiology in mature GINs and analyzed relative properties in sMDD and CTRL groups at day 70. Also, we reanalyzed the RNA sequencing data focusing results on GABAergic interneurons and added the cell composition analysis, and developmental trajectory analysis in this revision. Finally, we corrected typos and grammar mistakes and polished our manuscript to make our research more readable and accepted by readers.

(B) The manuscript is not clearly written. The title and the abstract do not articulate the potentially exciting discovery of the phenotypes in sMDD neurons and the rescue by HTR2C overexpression or Trazodone. There are many important typos and grammatically issues that need to be fixed in order for the manuscript to become a permanent record.
Thanks for the reviewer's important suggestion. In this revision, we have corrected our typos and grammar mistakes carefully and also polished the language. We believe the revised manuscript could be more readable for readers.

Specific issues:

(1) Fig. 1c, the ratio of GABA+/Tuj1+ cells might not reflect the situation accurately. Need to show GABA+/MAP2+ and GABA+/DAPI ratios. Otherwise, the data may not match the scRNAseq data of Fig. 4b, which does not appear to show that high percentage of mIN or eIN among neurons.
We appreciate the reviewer's valuable suggestions. In this revision, we performed immunofluorescence staining of GABA/MAP2 and GABA/HO on GABAergic interneurons at day 35 and added GABA+/MAP2+ ratios (ranging from 69.91% to 81.54%) and GABA+/HO+ ratios (ranging from 49.63% to 72.43%) to our results.

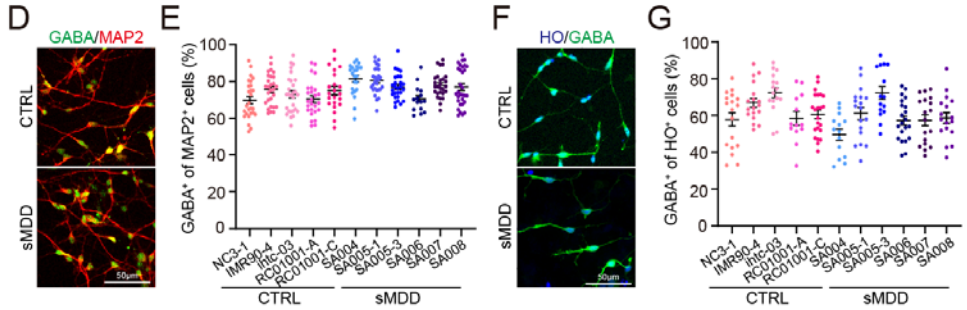

Figure EV1

D. Representative images of GABAergic interneurons from CTRL and sMDD cell lines staining GABA and MAP2. Scale bar = 50 μm.
E. The proportion of $GABA^+$ cells of $MAP2^+$ neurons from 5 CTRL cell lines and 6 sMDD cell lines derived GABAergic interneurons at day 35. n≥15.
F. Representative images of GABAergic interneurons from CTRL and sMDD cell lines staining HO and GABA. Scale bar = 50 μm.
G. The proportion of $GABA^+$ neurons of HO^+ cells from 5 CTRL cell lines and 6 sMDD cell lines derived GABAergic interneurons at day 35. n≥13.

(2) How was Sholl analysis done if you did not sparsely label single neurons in Fig. 1?

It is problematic to do morphological measurements when the neurons are still growing their processes at day 35. Please do Sholl analysis and also measure neurite length, branch number etc. in mature neurons at day 60 or later.

Thanks for the reviewer's questions. In our results, we performed Sholl analysis over low-density adherent GABAergic interneurons to distinguish each neuron at early stage. Therefore, the single neuron did not interact with other neurons at day 35.

According to the reviewer's suggestion, we did Sholl analysis and measured longest neurite length, and primary branch number at day 65 in this revision. The results were consistent with our finding at day 35.

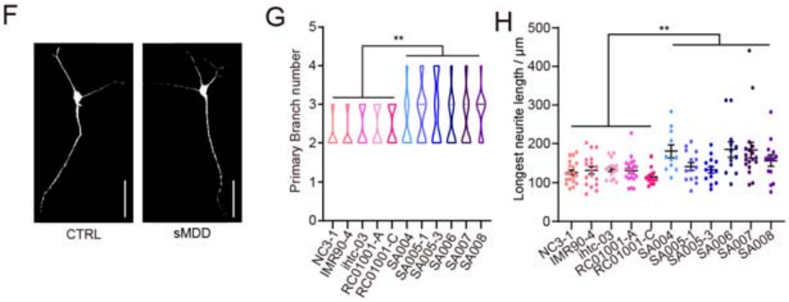

Figure 1

F. Representative images of GINs from CTRL and sMDD at day 65. Scale bar = 50 μm.

G. Quantification of primary branch numbers of GINs shown in 5 CTRL cell lines and 6 sMDD cell lines at day 65, n⩾11, Nested t-test, **p=0.0016 for CTRL versus sMDD.

H. Quantification of longest neurite length of GINs shown in 5 CTRL cell lines and 6 sMDD cell lines at day 65, n⩾11, Nested t-test, **p=0.0075 for CTRL versus sMDD.

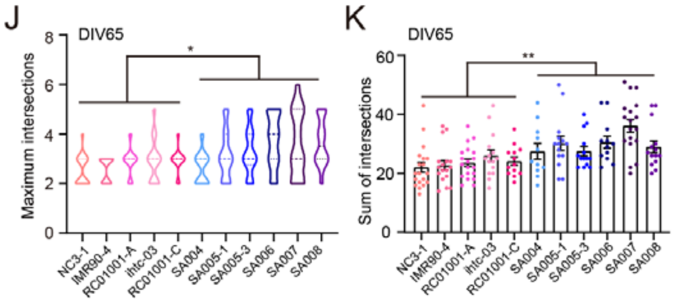

Figure EV1

J. Quantification of maximum of intersections by Sholl analysis shown in 5 CTRL cell lines and 6 sMDD cell lines at day 65, n=83 neurons from both CTRL groups and sMDD groups. Nested t-test, *p=0.0118 for CTRL versus sMDD.

K. Quantification of sum of intersections by Sholl analysis shown in 5 CTRL cell lines and 6 sMDD cell lines at day 65, n=83 neurons from both CTRL groups and sMDD groups. Nested t-test, **p=0.0027 for CTRL versus sMDD.

(3) Frankly, Fig. 1 reports a phenotype very distinct from the rest of the study. It does not need to be in this manuscript, unless the authors can connect the morphological phenotype to the other phenotypes. For example, does Trazodone make the neurites of sMDD neurons less elaborate?

Thanks for the reviewer's valuable suggestions. In this revision, we have added the result of restored elaboration of sMDD with the treatment of trazodone (Trzd).

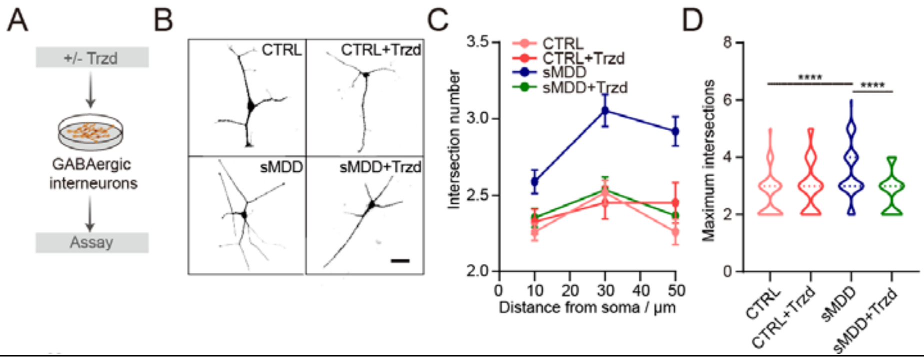

Figure 6

A. Representative images of single neuron from CTRL, CTRL+Trzd, sMDD and sMDD+Trzd GINs. Scale bar = 25μm.

B. Representative line chart of Sholl intersection number over distance from soma in CTRL, CTRL+Trzd, sMDD and sMDD+Trzd GINs at day 45.

C. Quantification of maximum intersections from CTRL, CTRL+Trzd, sMDD and sMDD+Trzd GINs via Sholl analysis. CTRL, n=81 neurons from 5 cell lines; CTRL+Trzd, n=81 neurons from 5 cell lines; sMDD, n=73 neurons from 6 cell lines; sMDD+Trzd, n=71 neurons from 6 cell lines. One-way ANONA, ****$p < 0.0001$ for CTRL versus sMDD and sMDD versus sMDD+Trzd.

D. Quantification of sum of intersections from CTRL, CTRL+Trzd, sMDD and sMDD+Trzd GINs via Sholl analysis. CTRL, n=81 neurons from 5 cell lines; CTRL+Trzd, n=81 neurons from 5 cell lines; sMDD, n=73 neurons from 6 cell lines; sMDD+Trzd, n=71 neurons from 6 cell lines. One-way ANONA, ****$p < 0.0001$ for CTRL versus sMDD; ***$p=0.0002$ for sMDD versus sMDD+Trzd.

(4) Fig. 2, can the frequency of evoke action potentials be analyzed? Do the neurons show spontaneous action potentials?

We appreciate the reviewer's valuable suggestions. In this revision, we have analyzed the frequency of evoked action potential, which were increased in sMDD groups (A).s We did not record positive spontaneous action potentials at day 45 successfully, while we recorded several spontaneous action potentials (10% over the recorded neurons) at day 70 in MDD group (B).

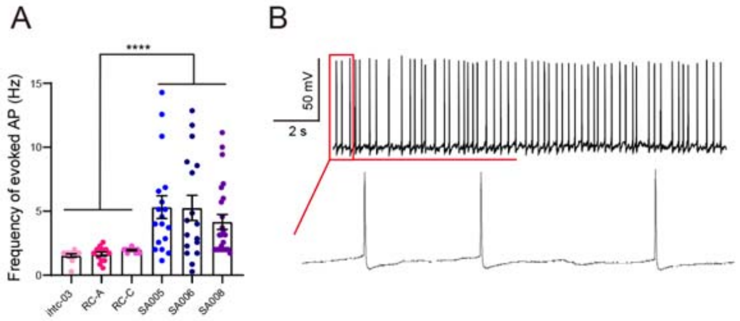

Figure a

A. Analysis of frequency of evoked AP (CTRL, n=35 neurons from 3 lines; sMDD, n=55 neurons from 3 lines).

B. Representative spontaneous action potential's traces from sMDD.

(5) Fig. 2b,d: evoked action potentials in control neurons appeared to be very abnormal. In addition, Na+ currents were very small in control neurons. It seems that

the neurons are simply not mature enough to produce action potentials in a reliable manner. Please do electrophysiology in mature neurons at day 70 or later.

We appreciate the important suggestion. In this revision, we have performed electrophysiology in mature neurons at day 70, which are consistent with the results at day 45.

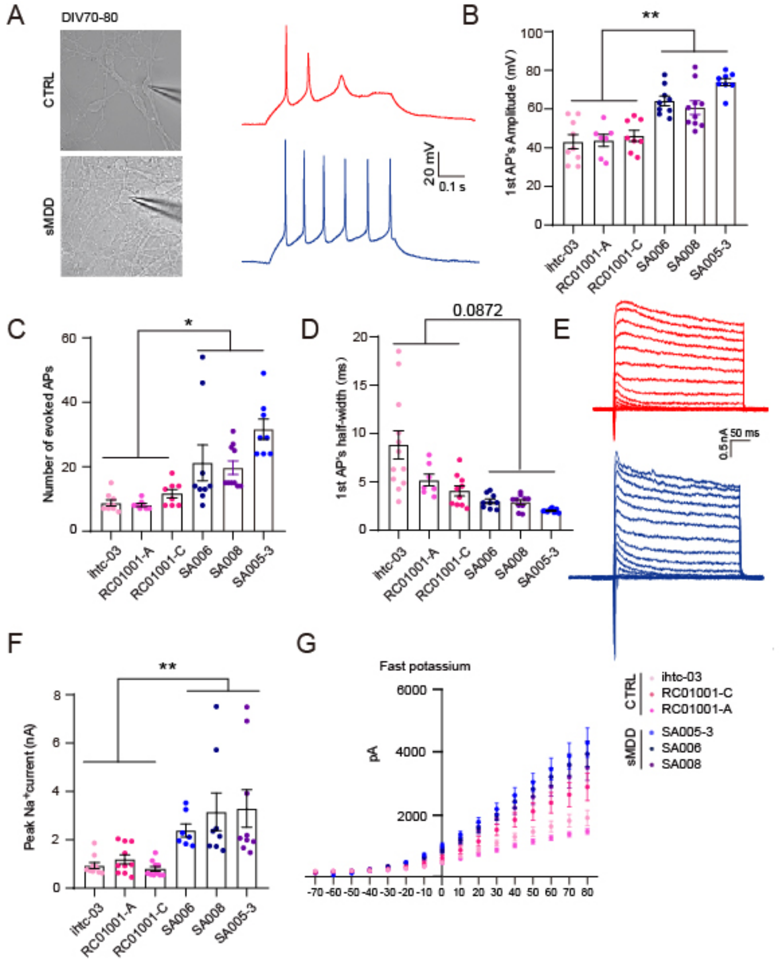

Figure EV2

A. Schematic diagram and representative images illustrating the method for whole-cell patch-clamp recording. And representative electrophysiological traces of AP at a holding potential of -70mV from GINs in sMDD and CTRL groups.

B. The amplitude of first AP generated in response to a 10-pA injection (CTRL, n=23 neurons from 3 lines; sMDD, n=25 neurons from 3 lines).

C. Average total number of APs evoked during 500ms stepwise depolarization (CTRL, n=23 neurons from 3 lines; sMDD, n=25 neurons from 3 lines).

D. The half width of first AP generated in response to a 10-pA injection (CTRL, n=23 neurons from 3 lines; sMDD, n=25 neurons from 3 lines).

E, F. Sample traces of Na+/K+ currents recorded from GINs in sMDD and CTRL groups. Average peak values of Na+ currents (CTRL, n=30 neurons from 3 lines; sMDD, n=24 neurons from 3 lines). Nested t test, $**p < 0.01$; $***p < 0.001$; $****p < 0.0001$.

G. Average fast potassium currents of GINs in sMDD and CTRL groups (CTRL, n=30 neurons from 3 lines; sMDD, n=24 neurons from 3 lines).

(6) Related to (3), evoked action potentials appeared to be very unstable, particularly in control neurons. Thus, the authors compared the properties of the first AP. This is highly problematic. A train of AP should have the same amplitude. This basic property of neurons is simply not seen in the recordings, casting doubts on how good the neuronal differentiation was.

We thank the reviewer raised important points. In our results, we analyzed the first AP evoked by current injection, mainly followed by the articles "*Mol Psychiatry. 2020 May;25(5):1094-1111; Biol Psychiatry. 2020 Jul 15;88(2):139-149*", which described the method for analyzing the first AP which induced by in vivo mouse neurons and in vitro iPSC-derived neurons, respectively.

Fig. 2 Sirtuin 1 (SIRT1) is required for normal neuronal excitability and excitatory synaptic transmission in layer V pyramidal neurons in the prelimbic medial prefrontal cortex (mPFC). **a1** SIRT1 immunoreactivity in the mPFC. PrL prelimbic, IL infralimbic. **a2** Schematic illustration of recordings from layer V pyramidal neurons in the prelimbic mPFC in acute brain slices. **a3–a12** Intrinsic neuronal excitability. **a3** Depolarizing current injections evoke trains of action potentials (APs) in PrL PFC pyramidal neurons. **a4** Number of APs elicited by depolarizing current injection. **a5** Rheobase current. **a6** Input resistance. **a7** Resting membrane potential. **a8** The first AP evoked by current injection. **a9** AP threshold. **a10** AP amplitude. **a11** AP half-width. **a12** Afterhyperpolarization (AHP). $n = 14$ neurons

Mol Psychiatry. 2020 May;25(5):1094-1111
(Page 10)

Spike shape analysis

The first evoked action potential was used for spike shape analysis (with the lowest injected current needed for eliciting an action potential). Spike threshold was the membrane potential at which the slope of the depolarizing membrane potential increased drastically, resulting in an action potential (the first maximum in the second derivative of the voltage vs. time). The 5-ms AHP amplitude was calculated as the difference between the threshold for spiking and the value of the membrane potential 5 ms after the potential returned to cross the threshold value at the end of the action potential. The spike amplitude was calculated as the difference between the maximum membrane potential during a spike and the threshold. Action potential width was calculated as the time it took the membrane potential to reach half the spike amplitude in the rising part of the spike to the descending part of the spike (Full Width at Half Maximum).

Biol Psychiatry. 2020 Jul 15;88(2):139-149
(Supplemental Material Page 2)

We totally agree with your point that the train of AP was the same amplitude when the neuron was in mature status. However, our iPSC-derived neurons are not as mature as the ones in mice brains. Indeed, the traces of evoked action potential in our article are similar to the trace in the following articles: "*Biol Psychiatry. 2020 Jul 15;88(2):139-149*" and "*Nature. 2015 Nov 5;527(7576):95-9*", which shows the evoked action potential of neurons derived from human iPSC.

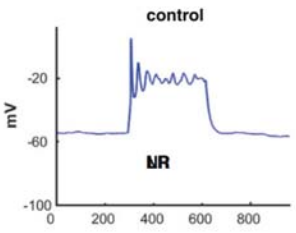

Biol Psychiatry. 2020 Jul 15;88(2):139-149
(Page 5)

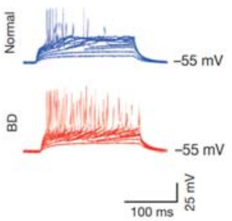

Nature. 2015 Nov 5;527(7576):95-9
(Page 3)

Therefore, we speculate the reason for the unstable evoked action potentials could be the neuron was still immature at D45. Indeed, we found the evoked action potentials were more stable at D70 in this revision.

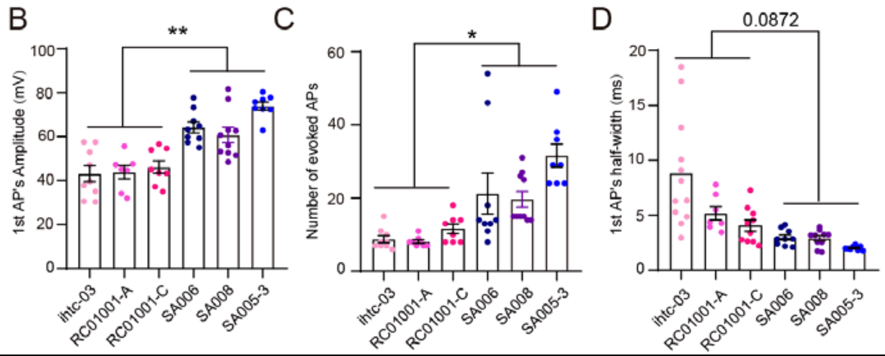

Figure EV2

B. The amplitude of first AP generated in response to a 10-pA injection at days 70-80 (CTRL, n=23 neurons from 3 lines; sMDD, n=25 neurons from 3 lines).

C. Average total number of APs evoked during 500ms stepwise depolarization at days 70-80 (CTRL, n=23 neurons from 3 lines; sMDD, n=25 neurons from 3 lines).

D. The half width of first AP generated in response to a 10-pA injection at days 70-80 (CTRL, n=23 neurons from 3 lines; sMDD, n=25 neurons from 3 lines).

Also, we have analyzed basic properties of GINs in this revised manuscript, and the results showed no significant difference between CTRL and sMDD (Table A).

	Ihtc-03	RC-A	RC-C	SA005-3	SA006	SA008
**RPM（mV）**	-77.69 ± 3.574	-63.71 ± 4.528	-67.73 ± 7.516	-49.03 ± 3.261	-54.43 ± 3.977	-56.6 ± 2.632
**Rin(GΩ)**	0.4524 ± 0.06942	0.4017 ± 0.07221	0.5464 ± 0.07107	0.4566 ± 0.04063	0.346 ± 0.0429	0.3712 ± 0.08877
**AP Threshold (mV)**	-32.74 ± 1.574	-31.81 ± 5.397	-20.57 ± 4.147	-35.12 ± 3.449	-29.9 ± 3.443	-34.6 ± 1.69
**Decay time(ms)**	30.5 ± 1.762	39.06 ± 4.757	36.78 ± 4.04	33.67 ± 2.759	22.48 ± 2.383	27.66 ± 2.242
**fAHP(mV)**	-7.328 ± -1.126	-4.089 ±- 0.6411	-8.983 ±- 1.608	-7.983 ± -1.133	-8.024 ± -1.293	-6.64 ± -0.7138

(7) Fig. 4c is confusing and not described in detail. Is "total" the sum of all other bars?

Thanks for the reviewer's question. In this Fig.4c, 'Total' represents all the genes associated with a given psychiatric disorder in the PsyGeNET database, while '100% association' represents the genes positively associated with the disorder and '100% no association' represents the genes negatively associated with the disorder. We also mention these descriptions in figure legends.

(8) Fig. 4d is unclear on what are being compared in the GO analysis, control vs. sMDD scRNAseq data in three different types of cells (GABAergic neurons, GABAergic progenitors and NSC)? It would be clearer if the data are segregated and focused on GABAergic neurons. The study does not address anything on GABAergic progenitors or NSCs. There is no need to show them. So is the format for Fig. 5h.

We appreciate the reviewer's valuable suggestions. For the original Fig 4D and Fig 5H, we aimed to annotate each GO term with a specific cell type so that the benefits of scRNA-seq could be realized. According to the reviewer's suggestions, we segregated all the data of GABAergic interneurons from the total scRNA-seq data. Then we performed GSEA analysis, especially on the GIN clusters. As a result, the original Fig 4D and Fig 5H were replaced by the following revised figures:

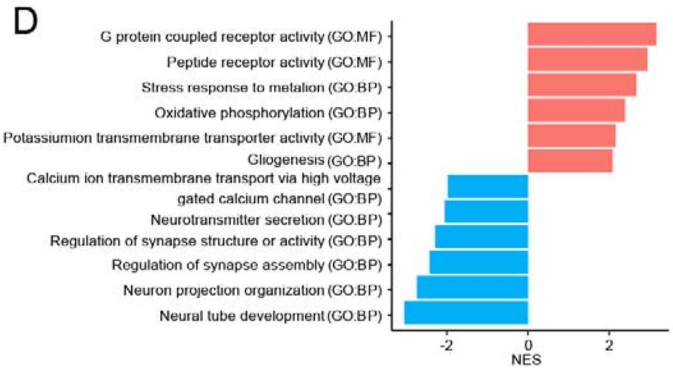

Figure 4

D. Gene set enrichment analysis (GSEA) for GIN clusters, which was based on Ontology gene sets of differentially expressed genes (DEGs). Presented GO terms are all significantly changed (adjusted p-value < 0.05).

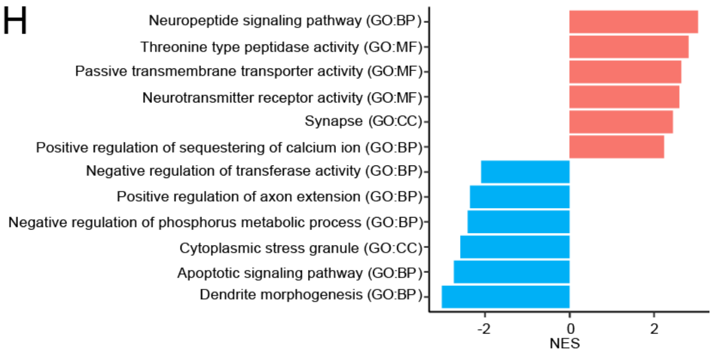

Figure 5

H. Gene set enrichment analysis (GSEA) results indicated altered GO terms after Trzd treatment (adjusted p-value < 0.05). Upregulated terms were colored with red, while downregulated terms were colored with blue.

(9) Fig. 5j needs clarification. What does it mean that some CAMK genes are above the line and some are below the line?

Thanks for the reviewer's questions. In Fig. 5J, we attempted to do correlation analysis on foldchanges of sMDD+Trzd vs sMDD and Control vs sMDD, to figure out whether Trzd could restore the calcium channel function of sMDD GINs. Actually, the line in this figure is a regression line, which displays the connection between scatter data. We use it to summarize the expression changes of CAMK genes between sMDD+Trzd vs sMDD and Control vs sMDD groups into a single-line model. Normally, the points float up and down around the line, since it indicates predicted connections, not actual values. We totally agree with the reviewer that positive correlation should meet the conditions of correlation coefficient R > 0 and p < 0.05. We apologize that we miss the p-value in previous figure. In this revision, we add the p-value in Fig. 5J.

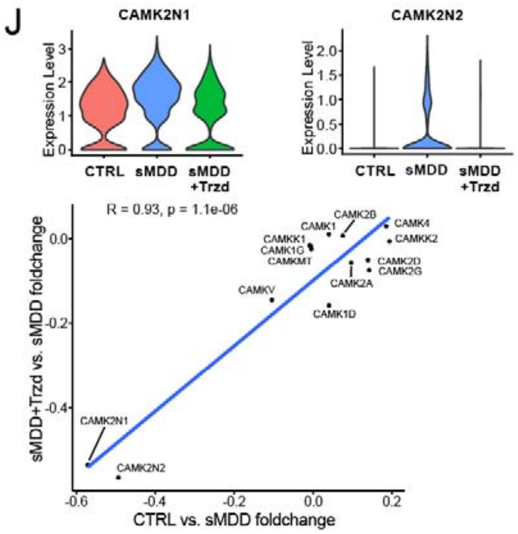

Figure 5
J. Top: Violin plots showing the expression level of *CAMK2N1* and *CAMK2N2* in three groups. Bottom: Scatter plot showing the correlations of foldchanges according to the expression of genes encoding CaMK family proteins between CTRL GINs and sMDD GINs and between sMDD with Trzd GINs and sMDD GINs. A linear regression line is added to the plot based on these two variables.

(10) Resolution of SupFig. 1 is too low, particularly for Fig. S1c. It is hard to see the neurons. This is very important and allows the reviewers to judge how good the differentiation was.

We apologize for the low resolution of images and thank for the reviewer's valuable suggestions. In this revision, we have improved the resolution of Figure EV1.

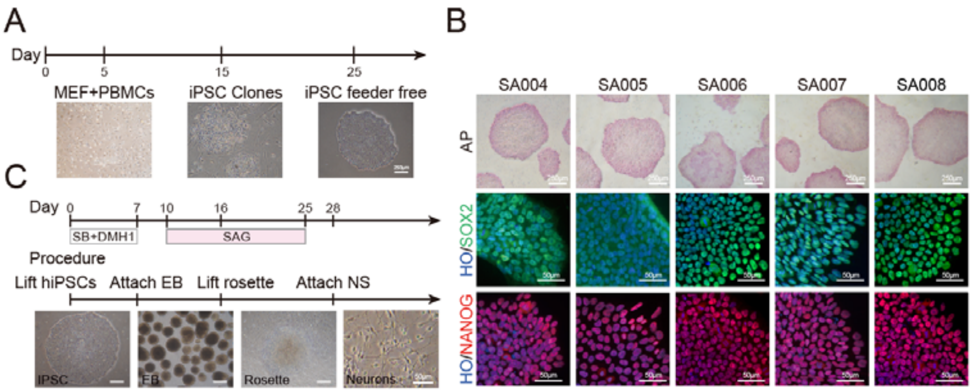

Figure EV1
A. Scheme illustrating the generation of iPSCs from CTRL and sMDD patients' peripheral blood mononuclear cell (PBMC). Scale bar = 250 μm. (MEF, Mouse Embryonic Fibroblast)
B. Alkaline phosphatase staining and SOX2, NANOG immunostaining in undifferentiated iPSCs from 5 sMDD patients. Scale bar is shown in the images.
C. Schematic diagram of iPSC differentiation to GINs. The former three images: scale bar = 250 μm. The last image: scale bar = 50 μm.

(11) Does the bulk RNAseq data show a significant difference in the expression levels of HTR2C? This needs to be documented along Fig. S3c.

Yes, we found that the expression level of HTR2C was significantly reduced in bulk RNAseq data. The detailed fold change has been shown below:

	baseMean	log2FoldChange	lfcSE	stat	pvalue	padj
HTR2C	193.57496	-2.61354762	0.597966	-4.370729	1.24E-05	0.0027233

Also, in Fig. 4E, we have shown the expression of HTR2C by heatmap, which is included in the Neuroactive ligand-receptor interaction and Calcium signaling pathway.

(12) Fig. 6: how did trazodone selectively change the electrophysiological properties and intracellular Ca2+ signals only in patient neurons, but not in control neurons? Does trazodone induce the expression of HTR2C only in patient, but not in control neurons? The trace in Fig. 6g and j for sMDD+Trzd appeared as if the neurons become immature. The sharp outward A-type K+ current in Fig. 6j was not seen in Fig. 2g.

We thank the reviewer raised a very important question. Calcium channels in the cell membrane are coupled with HTR2C, and calcium channels own a self-negative feedback regulation mechanism: too much calcium influx will cause calcium-dependent inactivation, which will lead to a reduction of calcium influx (*Amy Lee et al., Nature, 1999; 399(6732):155-9*). Therefore, trazodone did not change the intracellular Ca2+ signals in control neurons might be due to the self-negative feedback regulation mechanism.

The sharp outward A-type K+ current may be caused by reversal potential. When we inject different voltages, the chloride ions are driven to equilibrium by electric force, which will not affect the statistical results. In this revision, we have changed the representative trace of Fig. 6M.

M

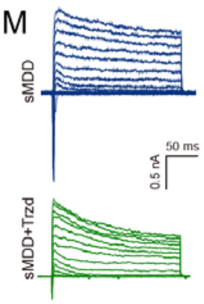

Figure 6

M. Sample traces of Na+/K+ currents recorded from GINs in sMDD and CTRL groups.

(13) The study reports electrophysiological results from a very large number of neurons. It is highly challenging to record that many neurons. It would demonstrate the rigor of the study by putting the summary Excel file of the electrophysiological data in the supplemental information.

We appreciate the reviewer's valuable suggestions. In this revision, we have organized the electrophysiological data of D45 and D70 as supplementary information uploaded online.

(14) All RNAseq data (sc and bulk) must be deposited to GEO so others can examine the data after publication. Please provide GEO access numbers of the deposited data.
Thanks for the reviewer's suggestions. We have uploaded all raw data and downstream files to GSE208438, including bulk RNAseq and scRNAseq. Meanwhile, we added GEO access numbers (GSE208438) in the method part of our article.

(15) Is GIN for GABAergic interneuron? Need to define it.
Yes. In this revision, we have defined it when first mentioned in Abstract and Introduction part.

(16) Page 3, line 93, there are six lines of iPSCs, but the text mentioned five. Is SA005-1 and SA005-3 from the same subject? Need to clarify.
We apologize for not clarifying this issue clearly. Indeed, these two cell lines SA005-1 and SA005-3 are from the same subject. In this revision, we have clarified this point in the first part of result.

(17) Should "committed suicide at least once" be "attempted suicide at least once". Death can only occur once.
We thank the great suggestion. We have changed the description "committed suicide at least once" into "attempted suicide at least once".

(18) There are many other typos and grammatical issues that require careful proofreading.
We apologize for these typos and grammatical issues. In this revision, we have corrected these problems carefully for better reading.

(19) The discussion is too long and some parts of it are speculative or superfluous. Please consider spending more words on describing the results than on speculative discussions.
Thanks for the reviewer's constructive suggestion. In this revision, we have spent more words describing the results instead of speculative discussion.

Referee #2 (Remarks for Author):

The manuscript "Depressive patient-derived GABA interneurons reveal abnormal neural activity associated with HTR2C," by Lu et al reports that GABAergic interneurons (GINs) derived from iPSCs of major depressive disorder patients with suicide behavior (sMDD) exhibit abnormal neuronal morphology and activity. Transcriptomic analyses at both single cell- and bulk- levels further reveal a decreased expression of serotoninergic receptor 2C (5-HT2C). This study goes on to show that restoring the expression of 5-HT2C using its agonist or genetic approach could rescue the deficits of neuronal activity in sMDD GINs. Overall, this is an excellent and well-written manuscript describing novel and important findings suitable for publication in EMBO Molecular Medicine, with minor revisions.

We appreciate the reviewer's positive comments on our work.

Major comments:

1. The authors observed an increased neurite branches and neural complexity in the sMDD GINs. However, based on the culture density shown in Fig. 1b, it is not clear how the morphology of individual neurons was analyzed. Furthermore, would these morphological phenotypes be developmental stage-dependent? A time-course study may provide more information.

Thanks for the reviewer's great questions. In our results, we performed Sholl analysis over low-density adherent GABAergic interneurons to distinguish each neuron at early stage. Therefore, the single neuron did not interact with any other neurons at day 35. In this revision, we further performed Sholl analysis and measured neurite length, and branch number at day 65, which were consistent with the day 35 results.

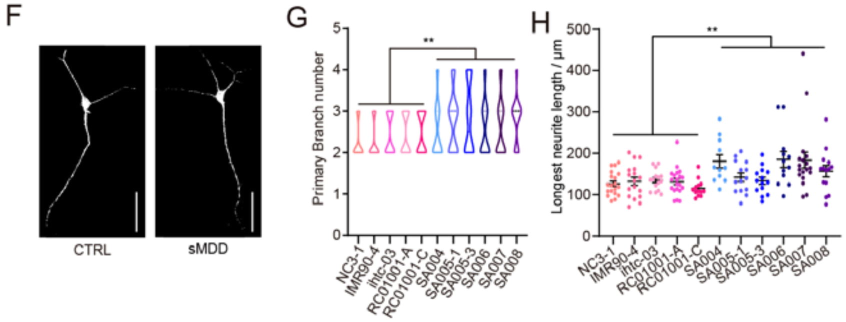

Figure 1
F. Representative images of GINs from CTRL and sMDD at day 65. Scale bar = 50 µm.
G. Quantification of primary branch numbers of GINs shown in 5 CTRL cell lines and 6 sMDD cell lines at day 65, n≥11, Nested t-test, **p=0.0016 for CTRL versus sMDD.
H. Quantification of longest neurite length of GINs shown in 5 CTRL cell lines and 6 sMDD cell lines at day 65, n≥11, Nested t-test, **p=0.0075 for CTRL versus sMDD.

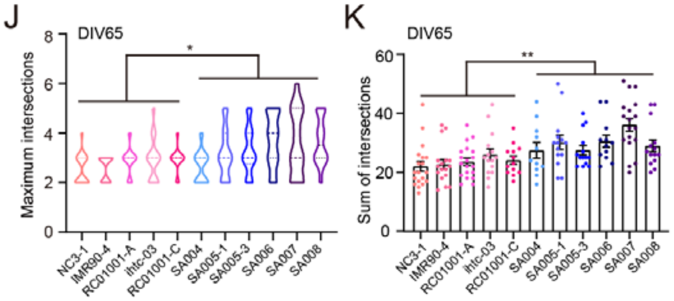

Figure EV1
J. Quantification of maximum of intersections by Sholl analysis shown in 5 CTRL cell lines and 6 sMDD cell lines at day 65, n=83 neurons from both CTRL groups and sMDD groups. Nested t-test, *p=0.0118 for CTRL versus sMDD.
K. Quantification of sum of intersections by Sholl analysis shown in 5 CTRL cell lines and 6 sMDD cell lines at day 65, n=83 neurons from both CTRL groups and sMDD groups. Nested t-test, **p=0.0027 for CTRL versus sMDD.

2. The observation of hyperexcitability in sMDD GINs is very interesting. A more

detailed characterization of the action potential kinetics, such as action potential threshold, decay time, and the fast afterhyperpolarization (fAHP), would further strengthen their conclusion. Moreover, are there changes in synaptic transmission, such as spontaneous and miniature inhibitory postsynaptic currents, in sMDD GINs?

We appreciate the reviewer's positive comments and valuable suggestions. In this revision, we have analyzed basic properties of neurons (Table A). We did not successfully record positive spontaneous action potentials at day 45, while we recorded several spontaneous action potentials (10% over the recorded neurons) at day 70 in MDD group, mainly because the iPSC-derived neurons were still immature.

	Ihtc-03	RC-A	RC-C	SA005-3	SA006	SA008
RPM (mV)	-77.69 ± 3.574	-63.71 ± 4.528	-67.73 ± 7.516	-49.03 ± 3.261	-54.43 ± 3.977	-56.6 ± 2.632
Rin(GΩ)	0.4524 ± 0.06942	0.4017 ± 0.07221	0.5464 ± 0.07107	0.4566 ± 0.04063	0.346 ± 0.0429	0.3712 ± 0.08877
AP Threshold (mV)	-32.74 ± 1.574	-31.81 ± 5.397	-20.57 ± 4.147	-35.12 ± 3.449	-29.9 ± 3.443	-34.6 ± 1.69
Decay time(ms)	30.5 ± 1.762	39.06 ± 4.757	36.78 ± 4.04	33.67 ± 2.759	22.48 ± 2.383	27.66 ± 2.242
fAHP(mV)	-7.328 ± -1.126	-4.089 ±- 0.6411	-8.983 ±- 1.608	-7.983 ± -1.133	-8.024 ± -1.293	-6.64 ± -0.7138

3. The single cell RNAseq on sMDD GINs provides novel and meaningful mechanistic insights. More analyses on the RNAseq data would be important for understanding the cellular phenotypes they observed in sMDD GINs. For example, are there changes in cell composition? Is the developmental trajectory altered in sMDD GINs?

We appreciate the reviewer's constructive comments. In this revision, we performed clustering procedure on all GINs and related results were shown in Figure EV3, which indicated the changes in neuron subtype composition and cell-to-cell interaction. To further study the molecular function of GIN clusters, we performed GO MF (Molecular Function) enrichment analysis on each subcluster and annotated them with a function term within the top5 results. The annotation means the more active function of a subcluster compared to the others. The results indicated that the number of GINs, in which sodium channel regulator activity is more active, is increased in the sMDD group. Furthermore, the number of GINs associated with calcium function, like Cadherin binding and Voltage-gated calcium channel activity, is greatly decreased in sMDD GINs. These results are consistent with our previous conclusions.

To investigate the trajectory of functional GINs, we also performed pseudotime analysis on GIN subclusters using monocle package and split the results by group. The results showed more GINs related to active sodium channel function between State 3 and State 2, but fewer calcium channel function active GINs after State 1 in the sMDD group, which are consistent with our previous findings.

The detailed results are shown below:

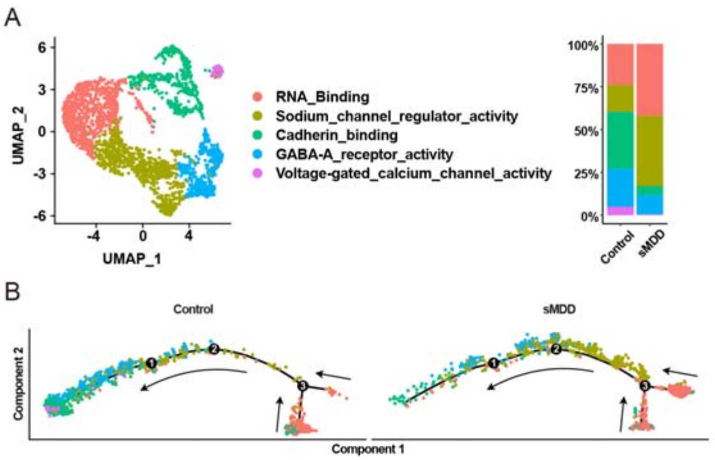

Figure b

A. Left: UMAP plot showing different subclusters of all GINs, which are annotated with enriched GO terms related to molecular function. Right: Proportion of cells belonging to each GIN subcluster in CTRL and sMDD samples.
B. Developmental trajectories of GIN subclusters in CTRL and sMDD samples.

4. The nearly complete rescue of calcium signaling and electrophysiological properties in sMDD GINs by trazodone hydrochloride treatment are notable. However, the lack of effect in controls are very surprising and warrant consideration in the discussion.

We thank the reviewer's valuable suggestions. Calcium channels in the cell membrane are coupled with HTR2C, and calcium channels own a self-negative feedback regulation mechanism: too much calcium influx will cause calcium-dependent inactivation, which will lead to a reduction of calcium influx (*Amy Lee et al., Nature, 1999; 399(6732):155-9*). Therefore, trazodone did not change the intracellular Ca2+ signals in control neurons might be due to the self-negative feedback regulation mechanism. In this revision, we have added the relative description as following shows: "*The 5-HT$_{2C}$R agonist Trzd had restored decreased calcium signaling and abnormal electrophysiological properties in sMDD groups but not in CTRL groups. The possible reason is that the self-negative feedback regulation of calcium channels in CTRL groups controls the balance of calcium influx*".

Referee #3 (Remarks for Author):

In this study, Lu and colleagues generated induced pluripotent stem cells (iPSCs) from major depressive disorder patients with suicidal behavior (sMDD), and differentiate the patient-derived iPSCs to GABAergic interneurons and ventral organoids. Gene profiling analysis showed neural activity related pathways were altered in sMDD groups, as well as the decreased expression levels of serotoninergic receptor 2C subtype (5-HT2C), which could be restored by overexpression of HT2RC. Moreover, the authors also used the sMDD patient-derived GABAergic interneurons to test small molecules. Overall, these findings are intriguing and provide valuable model for studying the mechanism of sMDD, as well as drug discoveries. There are a few aspects need further addressed for additional merits.

We appreciate the reviewer's positive comments on our work.

1. HT2RC was identified from bulk-seq data. Are other serotoninergic receptor subtypes altered in gene profiling data? Such as 5HT1A?

Thanks for the reviewer's question. The *HTR1A* gene, which encodes 5HT1A protein, was up-regulated in sMDD samples but with no significant difference (Table B).

The detailed results are shown below:

	baseMean	log2FoldChange	lfcSE	stat	pvalue	padj
HTR2C	193.57496	-2.61354762	0.597966	-4.370729	1.24E-05	0.0027233
HTR1A	5.1603883	1.72640455	1.2877158	1.3406721	0.1800269	NA

2. The authors tested calcium signaling by using Fluo-4 AM, which would examine the signal from non-GABA neurons. It would be helpful to make a claim in discussion.

Thanks for the reviewer's valuable suggestion. In our study, the differentiation ratio of GABAergic interneurons (GINs) was about 80% in total neurons, which indicates that most neurons we detected were GABA neurons. We add this issue in discussion as follows: "*We chose Fluo-4 AM to examine the calcium signaling of GINs whose differentiation ratio was about 80% in total neurons. Thus, the most neurons we detected were GINs.*"

3. There are many subtypes in GABAergic interneurons, for example, calretinin+ interneuron is an important subtype involved in calcium signaling. Is the ratio of calretinin subtype affected in sMDD groups?

Thanks for the reviewer's constructive question. In this revision, we found that the subtype of GABAergic interneurons calretinin (CR) was decreased in sMDD groups. We assessed CR expression by co-staining for TUJ1 at days 35, 40 and 50, respectively. We found no significant difference between the sMDD and CTRL groups at day 35. However, at day 40, the number of CR neurons in the sMDD group (11.8% ± 0.4%) was significantly fewer than that in the CTRL groups (17.4% ± 0.7%). Furthermore, the decreased population of CR neurons was obtained at day 50 (sMDD:16.0% ± 0.7%; CTRL:28.3% ± 1.2%).

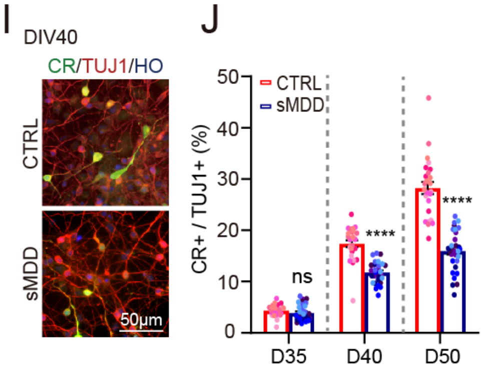

Figure 1

I. Representative images of GINs subtype calretinin (CR) expression. Scale bar = 50μm.

J. The proportion of CR^+ cells of $TUJ1^+$ neurons from both CTRL and sMDD iPSCs derived GINs at days 35, 40 and 50, respectively. (Red bar from 5 CTRL cell lines, blue bar from 6 sMDD cell lines). n=5 in each cell line. Two-way ANOVA for timepoints, ns: no significant at day 35, ****p < 0.0001 at days 40 and 50. Mean ratio ± SEM.

4. In figure 4e, the expression level of SST is decreased in sMDD groups. Somatostatin is a major GABA interneuron subtype in the brain. It might be helpful to identify whether somatostatin subtypes changed in sMDD groups.

Thanks for the reviewer's great suggestion. In this revised manuscript, we performed immunostaining for SST in GABAergic interneurons (GINs) at day 65. We observed the SST expression in sMDD groups was lower than that in CTRL groups.

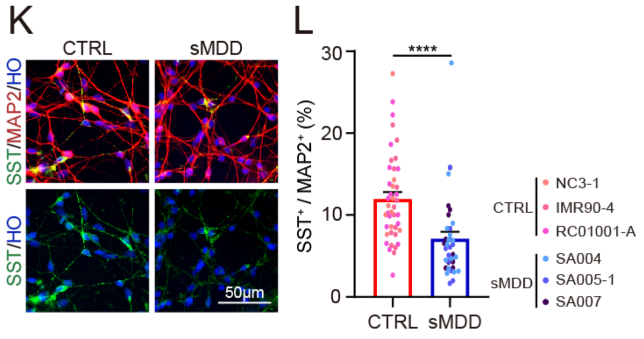

Figure 1
K. Representative images of GINs subtype somatostatin (SST) expression co-staining with mature neuron marker MAP2 in CTRL and sMDD GINs at day 65. Scale bar = 50μm.
L. The proportion of SST+ neurons of MAP2+ cells from CTRL and sMDD iPSCs derived GINs at day 65. n=43 in CTRL groups and n=37 in sMDD groups. T-test, ****p < 0.0001.

5. The authors claim the percentage of GABAergic interneurons is around 90%. What are the rest type of cells? Are they glial cells?

Thanks for the reviewer's great question. To explore the rest types of cells, we did immunostaining on astrocytes marker GFAP. We found that GFAP expression was less than 2% in total cells. Furthermore, in our previously published article, Huo and colleagues showed that the remaining cells in our GABA differentiation system were positive for dopaminergic neurons marker TH (<5%) and cholinergic neurons marker ChAT (<1%) (*Huo et al., Stem Cell Reports, 2018, 10(4):1251-1266*).

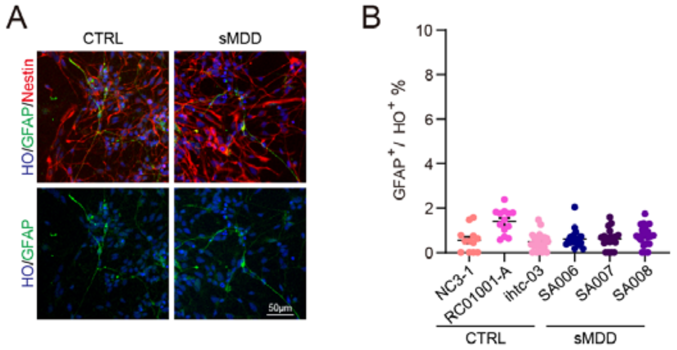

Figure c
A. Representative images of GFAP expression in iPSCs derived GABAergic interneurons at day 35. Scale bar = 50 μm.
B. The proportion of GFAP+ cells from 3 CTRL cell lines and 3 sMDD cell lines at day 35. n=49 in CTRL groups and n=62 in sMDD groups.

6. In introduction, the authors could include more literatures to introduce psychiatry

studies by using patient-derived iPSCs, especial brain organoids.

Thanks for the reviewer's valuable suggestion. In this revision, we have included more literature to introduce psychiatry studies by using patient-derived iPSCs, especially brain organoids in Introduction:

"Induced pluripotent stem cells (iPSCs) derived from neurological patients offer a potential model for studying pathogenesis and drug targets. Differentiation of iPSCs from bipolar disorder (BD) patients into hippocampal dentate gyrus-like neurons showed mitochondrial abnormalities and abnormal neuronal excitation in BD patients (Mertens J et al., Nature, 2015;527(7576):95-99). Furthermore, two studies exhibited abnormal disease pathophysiology with iPSCs derived forebrain neurons and organoids in psychiatric disorders and Fragile X syndrome (Wen Z et al., Nature, 2014;515(7527):414-418; Yunhee Kang et al., Nat Neurosci, 2021;24(10):1377-1391). Two recent studies reported longer neurites and serotonin-induced hyperactivity downstream of upregulated excitatory serotonergic receptors, respectively in SSRI-resistant MDD iPSC-derived serotoninergic neurons and default differentiated forebrain neurons (Vadodaria KC et al., Mol Psychiatry, 2019, 24(6):808-818; Vadodaria KC et al., Mol Psychiatry, 2019, 24(6):795-807). These iPSC-derived cells provided human cellular disease models for mechanism studies and drug discoveries. However, a human iPSC model for sMDD has not been reported."

7. In results, the authors claim they injected virus to knock down or overexpression of HT2RC in organoids, detail methodology should be described in methods.

Thanks for the reviewer's kind reminding. In the revised manuscript, we have added the detailed description of injecting virus into organoids in the methods part"

"***Injection of virus into organoids***

Use a 1ml pipette tip or a notch pipette to transfer organoids into a medium dish, and add 50ul of NIM medium to each organoid to prevent drying. Place virus on ice immediately after removing from -80°C freezer. Place the organoids under a microscope (Nikon SMZ800N). First, use a 4× microscope to find the location of the organoids, adjust the objective magnification according to the size of the organoids, and then use a micro syringe to start the injection. Each organoid was injected twice, 0.5 ul virus each time. After finishing the injection, add 50 ul of virus dilution solution (preparation of virus dilution solution: 500 ul NIM + 1:50 B27 + 1:2000 Polybrene + 1:100 PS + 1:100 virus stock solution). After incubation for 24 hours, the cells were transferred to T12.5 cell culture flasks, supplemented with 3 ml of NIM medium."

8. One reference (Mol Psychiatry 2019 Jun;24(6):795-807) in the manuscript showed decreased HT2RC levels in selective serotonin reuptake inhibitors (SSRI) resistant patient iPSC-derived neurons, but not in SSRI-responsive groups. It might be helpful to discuss the correlation between SSRI-resistant and suicidal behavior.

Thanks for the reviewer's valuable suggestion. In the revised manuscript, we have added the description of correlation between SSRI-resistant and suicidal behaviour in Discussion:

"According to a Poisson meta-analysis, the rates of attempted and committed suicide incidences were usually high in treatment-resistant depression (Bergfeld IO et al., J Affect Disord, 2018, 235:362-367)."

19th Oct 2022

Dear Prof. Liu,

Thank you for sending us your revised manuscript. We have now received the feedback from the two referees who agreed to re-review your manuscript. The referees are satisfied with the modifications and think the study is now suitable for publication.

Before we can formally accept your manuscript, we would ask you to address the following editorial-level issues:

1. Remove the blue color font.

2. Please upload the main and EV figures as high-resolution figure files as .eps, .tif, .jpg (one file per figure).

3. Data availability: please make sure the datasets are publicly accessible upon the acceptance of the manuscript.

4. Remove the heading "extended view tables" from the 3 EV table files.

5. Ethics

- In Materials and Methods, include a statement that informed consent was obtained from all subjects and that the experiments conformed to the principles set out in the WMA Declaration of Helsinki and the Department of Health and Human Services Belmont Report.

- Please update the related information in Author Checklist.

6. I have slightly modified and shortened the synopsis text (see attached). Please let me know if fine as is or if you would like to introduce further modifications.

7. Our data editors have seen the manuscript and made some comments and suggestions that need to be addressed (see attached file). Please send back a revised version (in track change mode), as we will need to go through the changes.

Please submit your revised manuscript within two weeks. I look forward to seeing a revised form of your manuscript as soon as possible.

Sincerely,
Jingyi

Jingyi Hou
Editor
EMBO Molecular Medicine

*** Instructions to submit your revised manuscript ***

*** PLEASE NOTE *** As part of the EMBO Publications transparent editorial process initiative (see our Editorial at https://www.embopress.org/doi/pdf/10.1002/emmm.201000094), EMBO Molecular Medicine will publish online a Review Process File to accompany accepted manuscripts.

In the event of acceptance, this file will be published in conjunction with your paper and will include the anonymous referee reports, your point-by-point response and all pertinent correspondence relating to the manuscript. If you do NOT want this file to be published, please inform the editorial office at contact@embomolmed.org.

To submit your manuscript, please follow this link:

https://embomolmed.msubmit.net/cgi-bin/main.plex

When submitting your revised manuscript, please include:

1) a .docx formatted version of the manuscript text (including Figure legends and tables)

2) Separate figure files*

3) supplemental information as Expanded View and/or Appendix. Please carefully check the authors guidelines for formatting Expanded view and Appendix figures and tables at https://www.embopress.org/page/journal/17574684/authorguide#expandedview

4) a letter INCLUDING the reviewer's reports and your detailed responses to their comments (as Word file).

5) The paper explained: EMBO Molecular Medicine articles are accompanied by a summary of the articles to emphasize the major findings in the paper and their medical implications for the non-specialist reader. Please provide a draft summary of your article highlighting
- the medical issue you are addressing,
- the results obtained and
- their clinical impact.
This may be edited to ensure that readers understand the significance and context of the research.
Please refer to any of our published articles for an example.

6) For more information: There is space at the end of each article to list relevant web links for further consultation by our readers. Could you identify some relevant ones and provide such information as well? Some examples are patient associations, relevant databases, OMIM/proteins/genes links, author's websites, etc...

7) Author contributions: the contribution of every author must be detailed in a separate section.

8) EMBO Molecular Medicine now requires a complete author checklist (https://www.embopress.org/page/journal/17574684/authorguide) to be submitted with all revised manuscripts. Please use the checklist as guideline for the sort of information we need WITHIN the manuscript. The checklist should only be filled with page numbers were the information can be found. This is particularly important for animal reporting, antibody dilutions (missing) and exact values and n that should be indicted instead of a range.

9) Every published paper now includes a 'Synopsis' to further enhance discoverability. Synopses are displayed on the journal webpage and are freely accessible to all readers. They include a short stand first (maximum of 300 characters, including space) as well as 2-5 one sentence bullet points that summarise the paper. Please write the bullet points to summarise the key NEW findings. They should be designed to be complementary to the abstract - i.e. not repeat the same text. We encourage inclusion of key acronyms and quantitative information (maximum of 30 words / bullet point). Please use the passive voice. Please attach these in a separate file or send them by email, we will incorporate them accordingly.

You are also welcome to suggest a striking image or visual abstract to illustrate your article. If you do please provide a jpeg file 550 px-wide x 400-px high.

10) A Conflict of Interest statement should be provided in the main text

11) Please note that we now mandate that all corresponding authors list an ORCID digital identifier. This takes <90 seconds to complete. We encourage all authors to supply an ORCID identifier, which will be linked to their name for unambiguous name identification.

Currently, our records indicate that the ORCID for your account is 0000-0003-2918-5398.

Please click the link below to modify this ORCID:
Link Not Available

12) The system will prompt you to fill in your funding and payment information. This will allow Wiley to send you a quote for the article processing charge (APC) in case of acceptance. This quote takes into account any reduction or fee waivers that you may be eligible for. Authors do not need to pay any fees before their manuscript is accepted and transferred to our publisher.

*Additional important information regarding Figures

Each figure should be given in a separate file and should have the following resolution:
Graphs 800-1,200 DPI
Photos 400-800 DPI

Colour (only CMYK) 300-400 DPI"

Figures are not edited by the production team. All lettering should be the same size and style; figure panels should be indicated by capital letters (A, B, C etc). Gridlines are not allowed except for log plots. Figures should be numbered in the order of their appearance in the text with Arabic numerals. Each Figure must have a separate legend and a caption is needed for each panel.

*Additional important information regarding figures and illustrations can be found at https://bit.ly/EMBOPressFigurePreparationGuideline. See also figure legend preparation guidelines: https://www.embopress.org/page/journal/17574684/authorguide#figureformat

The system will prompt you to fill in your funding and payment information. This will allow Wiley to send you a quote for the article processing charge (APC) in case of acceptance. This quote takes into account any reduction or fee waivers that you may be eligible for. Authors do not need to pay any fees before their manuscript is accepted and transferred to our publisher.

***** Reviewer's comments *****

Referee #2 (Remarks for Author):

The response to the first round of reviews is satisfactory. I support its publication in EMBO Molecular Medicine.

21st Oct 2022

Dear Prof. Liu,

We are pleased to inform you that your manuscript is accepted for publication and is now being sent to our publisher to be included in the next available issue of EMBO Molecular Medicine.

We would like to remind you that as part of the EMBO Publications transparent editorial process initiative, EMBO Molecular Medicine will publish a Review Process File online to accompany accepted manuscripts. If you do NOT want the file to be published or would like to exclude figures, please immediately inform the editorial office via e-mail.

Please read below for additional IMPORTANT information regarding your article, its publication and the production process.

Congratulations on your interesting work,

Jingyi

Jingyi Hou
Editor
EMBO Molecular Medicine

Follow us on Twitter @EmboMolMed
Sign up for eTOCs at embopress.org/alertsfeeds

*** *** *** IMPORTANT INFORMATION *** *** ***

SPEED OF PUBLICATION
The journal aims for rapid publication of papers, using using the advance online publication "Early View" to expedite the process: A properly copy-edited and formatted version will be published as "Early View" after the proofs have been corrected. Please help the Editors and publisher avoid delays by providing e-mail address(es), telephone and fax numbers at which author(s) can be contacted.

Should you be planning a Press Release on your article, please get in contact with embomolmed@wiley.com as early as possible, in order to coordinate publication and release dates.

LICENSE AND PAYMENT:

All articles published in EMBO Molecular Medicine are fully open access: immediately and freely available to read, download and share.

EMBO Molecular Medicine charges an article processing charge (APC) to cover the publication costs. You, as the corresponding author for this manuscript, should have already received a quote with the article processing fee separately. Please let us know in case this quote has not been received.

Once your article is at Wiley for editorial production you will receive an email from Wiley's Author Services system, which will ask you to log in and will present you with the publication license form for completion. Within the same system the publication fee can be paid by credit card, an invoice, pro forma invoice or purchase order can be requested.

Payment of the publication charge and the signed Open Access Agreement form must be received before the article can be published online.

PROOFS

You will receive the proofs by e-mail approximately 2 weeks after all relevant files have been sent o our Production Office. Please return them within 48 hours and if there should be any problems, please contact the production office at embopressproduction@wiley.com.

Please inform us if there is likely to be any difficulty in reaching you at the above address at that time. Failure to meet our

deadlines may result in a delay of publication.

All further communications concerning your paper proofs should quote reference number EMM-2022-16364-V3 and be directed to the production office at embopressproduction@wiley.com.

Thank you,

Jingyi Hou
Editor
EMBO Molecular Medicine

EMBO Press Author Checklist

Corresponding Author Name: Liu Yan, Guo Xing	
Journal Submitted to: EMBO Molecular Medicine	
Manuscript Number: EMM-2022-16364	

USEFUL LINKS FOR COMPLETING THIS FORM
The EMBO Journal - Author Guidelines
EMBO Reports - Author Guidelines
Molecular Systems Biology - Author Guidelines
EMBO Molecular Medicine - Author Guidelines

Reporting Checklist for Life Science Articles (updated January 2022)
This checklist is adapted from Materials Design Analysis Reporting (MDAR) Checklist for Authors. MDAR establishes a minimum set of requirements in transparent reporting in the life sciences (see Statement of Task: 10.31222/osf.io/9sm4x). Please follow the journal's guidelines in preparing your manuscript.
Please note that a copy of this checklist will be published alongside your article.

Abridged guidelines for figures
1. Data
The data shown in figures should satisfy the following conditions:
- the data were obtained and processed according to the field's best practice and are presented to reflect the results of the experiments in an accurate and unbiased manner.
- ideally, figure panels should include only measurements that are directly comparable to each other and obtained with the same assay.
- plots include clearly labeled error bars for independent experiments and sample sizes. Unless justified, error bars should not be shown for technical replicates.
- if n<5, the individual data points from each experiment should be plotted. Any statistical test employed should be justified.
- Source Data should be included to report the data underlying figures according to the guidelines set out in the authorship guidelines on Data Presentation.

2. Captions
Each figure caption should contain the following information, for each panel where they are relevant:
- a specification of the experimental system investigated (eg cell line, species name).
- the assay(s) and method(s) used to carry out the reported observations and measurements.
- an explicit mention of the biological and chemical entity(ies) that are being measured.
- an explicit mention of the biological and chemical entity(ies) that are altered/varied/perturbed in a controlled manner.
- the exact sample size (n) for each experimental group/condition, given as a number, not a range;
- a description of the sample collection allowing the reader to understand whether the samples represent technical or biological replicates (including how many animals, litters, cultures, etc.).
- a statement of how many times the experiment shown was independently replicated in the laboratory.
- definitions of statistical methods and measures:
  - common tests, such as t-test (please specify whether paired vs. unpaired), simple χ2 tests, Wilcoxon and Mann-Whitney tests, can be unambiguously identified by name only, but more complex techniques should be described in the methods section;
  - are tests one-sided or two-sided?
  - are there adjustments for multiple comparisons?
  - exact statistical test results, e.g., P values = x but not P values < x;
  - definition of 'center values' as median or average;
  - definition of error bars as s.d. or s.e.m.

Please complete ALL of the questions below.
Select "Not Applicable" only when the requested information is not relevant for your study.

Materials

Newly Created Materials	Information included in the manuscript?	In which section is the information available? (Reagents and Tools Table, Materials and Methods, Figures, Data Availability Section)
New materials and reagents need to be available; do any restrictions apply?	Not Applicable	

Antibodies	Information included in the manuscript?	In which section is the information available? (Reagents and Tools Table, Materials and Methods, Figures, Data Availability Section)
For **antibodies** provide the following information:<br>- Commercial antibodies: RRID (if possible) or supplier name, catalogue number and or/clone number<br>- Non-commercial: RRID or citation	Yes	Extended View Tables

DNA and RNA sequences	Information included in the manuscript?	In which section is the information available? (Reagents and Tools Table, Materials and Methods, Figures, Data Availability Section)
**Short novel DNA or RNA including primers, probes**: provide the sequences.	Yes	Extended View Tables

Cell materials	Information included in the manuscript?	In which section is the information available? (Reagents and Tools Table, Materials and Methods, Figures, Data Availability Section)
**Cell lines:** Provide species information, strain. Provide accession number in repository **OR** supplier name, catalog number, clone number, and/**OR** RRID.	Yes	Extended View Tables
**Primary cultures:** Provide species, strain, sex of origin, genetic modification status.	Not Applicable	
Report if the cell lines were recently **authenticated** (e.g., by STR profiling) and tested for mycoplasma contamination.	Yes	**Source data**

Experimental animals	Information included in the manuscript?	In which section is the information available? (Reagents and Tools Table, Materials and Methods, Figures, Data Availability Section)
**Laboratory animals or Model organisms:** Provide species, strain, sex, age, genetic modification status. Provide accession number in repository **OR** supplier name, catalog number, clone number, **OR** RRID.	Not Applicable	
**Animal observed in or captured from the field:** Provide species, sex, and age where possible.	Not Applicable	
Please detail **housing and husbandry conditions**.	Not Applicable	

Plants and microbes	Information included in the manuscript?	In which section is the information available? (Reagents and Tools Table, Materials and Methods, Figures, Data Availability Section)
**Plants:** provide species and strain, ecotype and cultivar where relevant, unique accession number if available, and source (including location for collected wild specimens).	Not Applicable	
**Microbes:** provide species and strain, unique accession number if available, and source.	Not Applicable	

Human research participants	Information included in the manuscript?	In which section is the information available? (Reagents and Tools Table, Materials and Methods, Figures, Data Availability Section)
If collected and within the bounds of privacy constraints report on age, sex and gender or ethnicity for all study participants.	Not Applicable	

Core facilities	Information included in the manuscript?	In which section is the information available? (Reagents and Tools Table, Materials and Methods, Figures, Data Availability Section)
If your work benefited from core facilities, was their service mentioned in the acknowledgments section?	Not Applicable	

Design

Study protocol	Information included in the manuscript?	In which section is the information available? (Reagents and Tools Table, Materials and Methods, Figures, Data Availability Section)
If study protocol has been **pre-registered, provide DOI in the manuscript**. For clinical trials, provide the trial registration number **OR** cite DOI.	Not Applicable	
Report the **clinical trial registration number** (at ClinicalTrials.gov or equivalent), where applicable.	Not Applicable	

Laboratory protocol	Information included in the manuscript?	In which section is the information available? (Reagents and Tools Table, Materials and Methods, Figures, Data Availability Section)
Provide DOI OR other citation details if **external detailed step-by-step protocols** are available.	Not Applicable	

Experimental study design and statistics	Information included in the manuscript?	In which section is the information available? (Reagents and Tools Table, Materials and Methods, Figures, Data Availability Section)
Include a statement about **sample size** estimate even if no statistical methods were used.	Not Applicable	
Were any steps taken to minimize the effects of subjective bias when allocating animals/samples to treatment (e.g. **randomization procedure**)? If yes, have they been described?	Not Applicable	
Include a statement about **blinding** even if no blinding was done.	Not Applicable	
Describe **inclusion/exclusion criteria** if samples or animals were excluded from the analysis. Were the criteria pre-established?<br><br>If sample or data points were omitted from analysis, report if this was due to attrition or intentional exclusion and provide justification.	Not Applicable	
For every figure, are **statistical tests** justified as appropriate? Do the data meet the assumptions of the tests (e.g., normal distribution)? Describe any methods used to assess it. Is there an estimate of variation within each group of data? Is the variance similar between the groups that are being statistically compared?	Not Applicable	

Sample definition and in-laboratory replication	Information included in the manuscript?	In which section is the information available? (Reagents and Tools Table, Materials and Methods, Figures, Data Availability Section)
In the figure legends: state number of times the experiment was **replicated** in laboratory.	Yes	Figure legends
In the figure legends: define whether data describe **technical or biological replicates**.	Not Applicable	

Ethics

Ethics	Information included in the manuscript?	In which section is the information available? (Reagents and Tools Table, Materials and Methods, Figures, Data Availability Section)
Studies involving **human participants**: State details of **authority granting ethics approval** (IRB or equivalent committee(s), provide reference number for approval.	Yes	Materials and Methods
Studies involving **human participants**: Include a statement confirming that **informed consent** was obtained from all subjects and that the experiments conformed to the principles set out in the WMA Declaration of Helsinki and the Department of Health and Human Services Belmont Report.	Yes	Materials and Methods
Studies involving **human participants:** For publication of **patient photos**, include a statement confirming that consent to publish was obtained.	Yes	Materials and Methods
Studies involving experimental **animals**: State details of **authority granting ethics approva**l (IRB or equivalent committee(s), provide reference number for approval. Include a statement of compliance with ethical regulations.	Not Applicable	
Studies involving **specimen and field samples:** State if relevant **permits** obtained, provide details of authority approving study; if none were required, explain why.	Not Applicable	

Dual Use Research of Concern (DURC)	Information included in the manuscript?	In which section is the information available? (Reagents and Tools Table, Materials and Methods, Figures, Data Availability Section)
Could your study fall under dual use research restrictions? Please check biosecurity documents and list of **select agents and toxins** (CDC): https://www.selectagents.gov/sat/list.htm	Not Applicable	
If you used a select agent, is the security level of the lab appropriate and reported in the manuscript?	Not Applicable	
If a study is subject to dual use research of concern regulations, is the name of the **authority granting approval and reference number** for the regulatory approval provided in the manuscript?	Yes	Acknowledgements

Reporting

The MDAR framework recommends adoption of discipline-specific guidelines, established and endorsed through community initiatives. Journals have their own policy about requiring specific guidelines and recommendations to complement MDAR.

Adherence to community standards	Information included in the manuscript?	In which section is the information available? (Reagents and Tools Table, Materials and Methods, Figures, Data Availability Section)
State if relevant guidelines or checklists (e.g., **ICMJE, MIBBI, ARRIVE, PRISMA**) have been followed or provided.	Not Applicable	
For **tumor marker prognostic studies,** we recommend that you follow the **REMARK** reporting guidelines (see link list at top right). See author guidelines, under 'Reporting Guidelines'. Please confirm you have followed these guidelines.	Not Applicable	
For **phase II and III randomized controlled trials**, please refer to the **CONSORT** flow diagram (see link list at top right) and submit the CONSORT checklist (see link list at top right) with your submission. See author guidelines, under 'Reporting Guidelines'. Please confirm you have submitted this list.	Not Applicable	

Data Availability

Data availability	Information included in the manuscript?	In which section is the information available? (Reagents and Tools Table, Materials and Methods, Figures, Data Availability Section)
Have **primary datasets** been deposited according to the journal's guidelines (see 'Data Deposition' section) and the respective accession numbers provided in the Data Availability Section?	Yes	Data Availability Section
Were **human clinical and genomic datasets** deposited in a public access-controlled repository in accordance to ethical obligations to the patients and to the applicable consent agreement?	Yes	Gene Expression Omnibus (GEO) under accession GSE208438
Are **computational models** that are central and integral to a study available without restrictions in a machine-readable form? Were the relevant accession numbers or links provided?	Not Applicable	
If publicly available data were reused, provide the respective **data citations in the reference list**.	Not Applicable	
