# Supplementary material for: Depressive patient‐derived GABA interneurons reveal abnormal neural activity associated with HTR2C
Source: EMBO Mol Med. 2022 Nov 14;15(1):e16364. doi: 10.15252/emmm.202216364 (PMC9832822; doi:10.15252/emmm.202216364)
Supplement: Supplementary file 5 — Source Data for Expanded View [file EMMM-15-e16364-s012.zip › Figure EV1.zip!/Figure EV1/Mycoplasma contamination testing of cell lines.pdf]

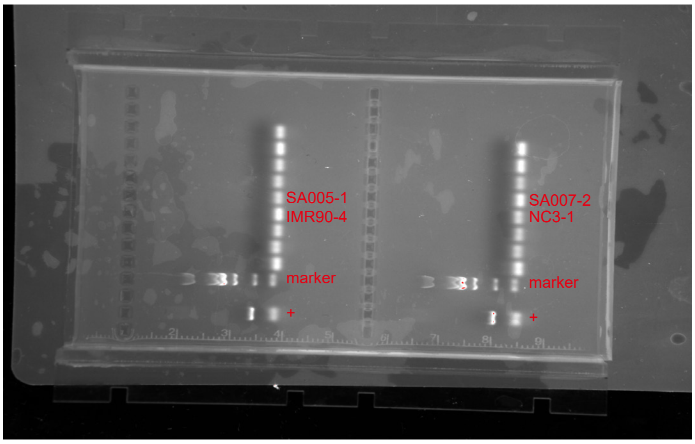
SA005-1
IMR90-4
marker
+
SA007-2
NC3-1
marker
+

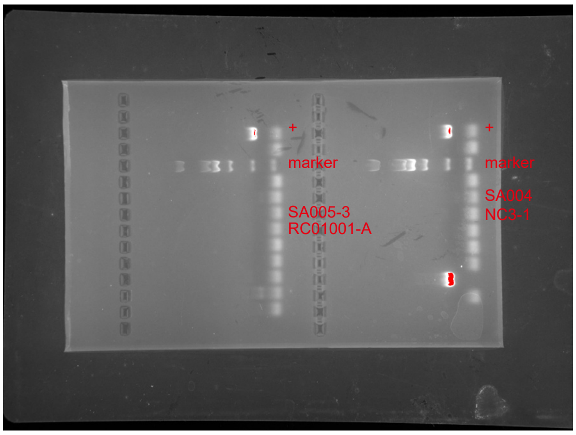
+
marker
SA005-3
RC01001-A
+
marker
SA004
NC3-1

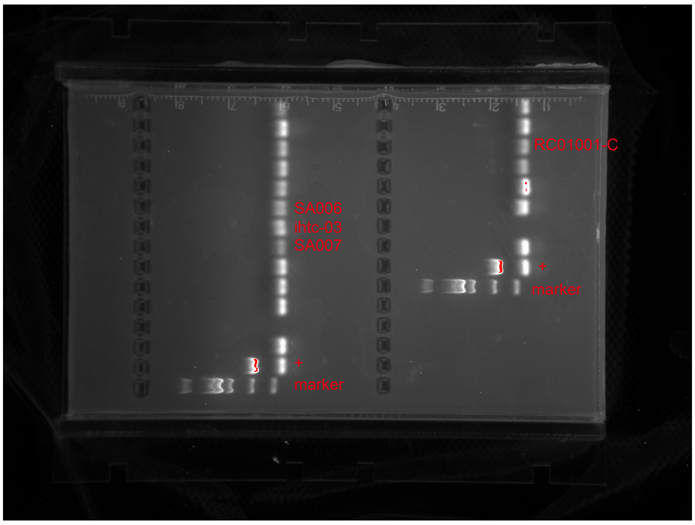
SA006
ihtc-03
SA007
+
marker
RC01001-C
+
marker

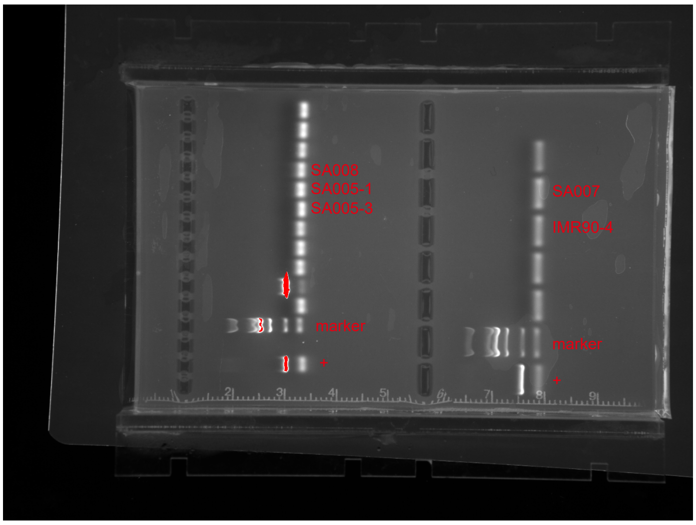
SA008
SA005-1
SA005-3
marker
+
SA007
IMR90-4
marker
+
